# CCL19 has potential to be a potential prognostic biomarker and a modulator of tumor immune microenvironment (TIME) of breast cancer: a comprehensive analysis based on TCGA database

**DOI:** 10.18632/aging.204081

**Published:** 2022-05-12

**Authors:** Jinyan Wang, Dongmei Qin, Lingling Ye, Li Wan, Fen Wang, Yan Yang, Yajun Ma, Hui Yang, Zhaohui Yang, Meili Chen, Wen Jiang, Quan’an Zhang

**Affiliations:** 1 Department of Oncology, Nanjing Jiangning Hospital, The Affiliated Jiangning Hospital of Nanjing Medical University, Nanjing, China; 2 Department of Pathology, Nanjing Jiangning Hospital, The Affiliated Jiangning Hospital of Nanjing Medical University, Nanjing, China; 3 Department of Oncology, The Second Hospital of Nanjing, Nanjing, China

**Keywords:** tumor microenvironment (TME), tumor immune microenvironment (TIME), CCL19, tumor-infiltrating immune cells (TICs), breast cancer

## Abstract

The development of cancer was determined by not only the intrinsic properties of cancer cells, but also the communication between cancer cells and tumor microenvironment (TME). We applied ESTIMATE and CIBERSORT algorithms to calculate the immune/stromal component and tumor-infiltrating immune cells (TICs) in TME of BC. The results showed that immune component in TME predicted patients’ survival and associated with progression of BC. Differentially expressed genes (DEGs) were primarily enriched in immune-related activities. Finally, CCL19 was acquired which shared the leading nodes in PPI network and was associated with patients’ survival. High expression of CCL19 predicted better prognosis and participated in progression of BC. Genes in CCL19 up-regulated group were enriched in immune-related activities and these functions might depend on the communications between CCL19 and multiple TICs in TIME. In conclusion, CCL19 functioned as a potential prognostic biomarker and a modulator of TIME in BC through communicating with various TICs.

## INTRODUCTION

Breast cancer (BC) is the most common cancer in women across the world, with an estimated 42,000 cancer-related deaths in the United States in 2020, and is the second leading cause of cancer-associated mortality [1]. Though, cytotoxic chemotherapy, including paclitaxel and carboplatin, has been widely used in the treatment of BC, the outcome of BC patients is not yet satisfying, especially for patients with triple-negative breast cancer (TNBC) [2]. Therefore, it is urgent to further explore the underlying mechanisms of carcinogenesis and therapeutics in BC.

Accumulating evidence has demonstrated that the tumor microenvironment (TME) played a significant role in the occurrence and progression of BC [3]. TME is made up of various cell types, such as fibroblasts, adipocyte, immune cells, inflammatory cells and blood and lymphatic vascular networks [4]. The communications between tumor cells and the surrounding TME obviously affect the genesis, progression and drug resistance of BC [5]. Recently, the concept of the tumor immune microenvironment (TIME) has attracted increasing interests, which is composed of tumor-infiltrating immune cells (TICs), including natural killer (NK) cells, T cells (CD8, CD4), Treg, dendritic cells (DCs), myeloid-derived suppressor cells (MDSCs), tumor-associated macrophages (TAMs) and so on [6]. Additionally, tumor cells exhibit the ability to inhibit the TIME and evade immune surveillance [7]. Meanwhile, TICs or immune-related genes in TIME act to predict the prognosis and therapeutic efficacy of cancer patients [8–10]. For instance, BC was previously recognized as a non-immunogenic tumor. Whereas, with the deepening of researches, it was found that tumor-infiltrating lymphocytes (TILs) were significantly associated with better prognosis in patients with early stage triple-negative and HER2-positive BC [11]. This relevance brought light to the immune-based therapeutics, and led to the application of immune checkpoint blockers for BC patients. Therefore, it is meaningful to deeply understand the correlation between TIME and prognosis of BC, and identify the novel biomarkers and potential precise therapeutic targets of BC.

Luckily, transcriptome profiling based on functional genomics analysis has shed light on the role of TME in BC. In our study, we assessed the ratio of immune/stromal component and the TIC proportion in TME of BC samples from The Cancer Genome Atlas (TCGA) database by using ESTIMATE and CIBERSORT algorithms. And then, we embarked from the differentially expressed genes (DEGs) obtained by comparison between immune component and stromal component in BC samples and clarified that C-C motif chemokine 19 (CCL19) functioned as a potential prognostic biomarker and a modulator of TIME in BC through communicating with various TICs in TIME.

## RESULTS

### Analysis procedure

**Figure 1:**
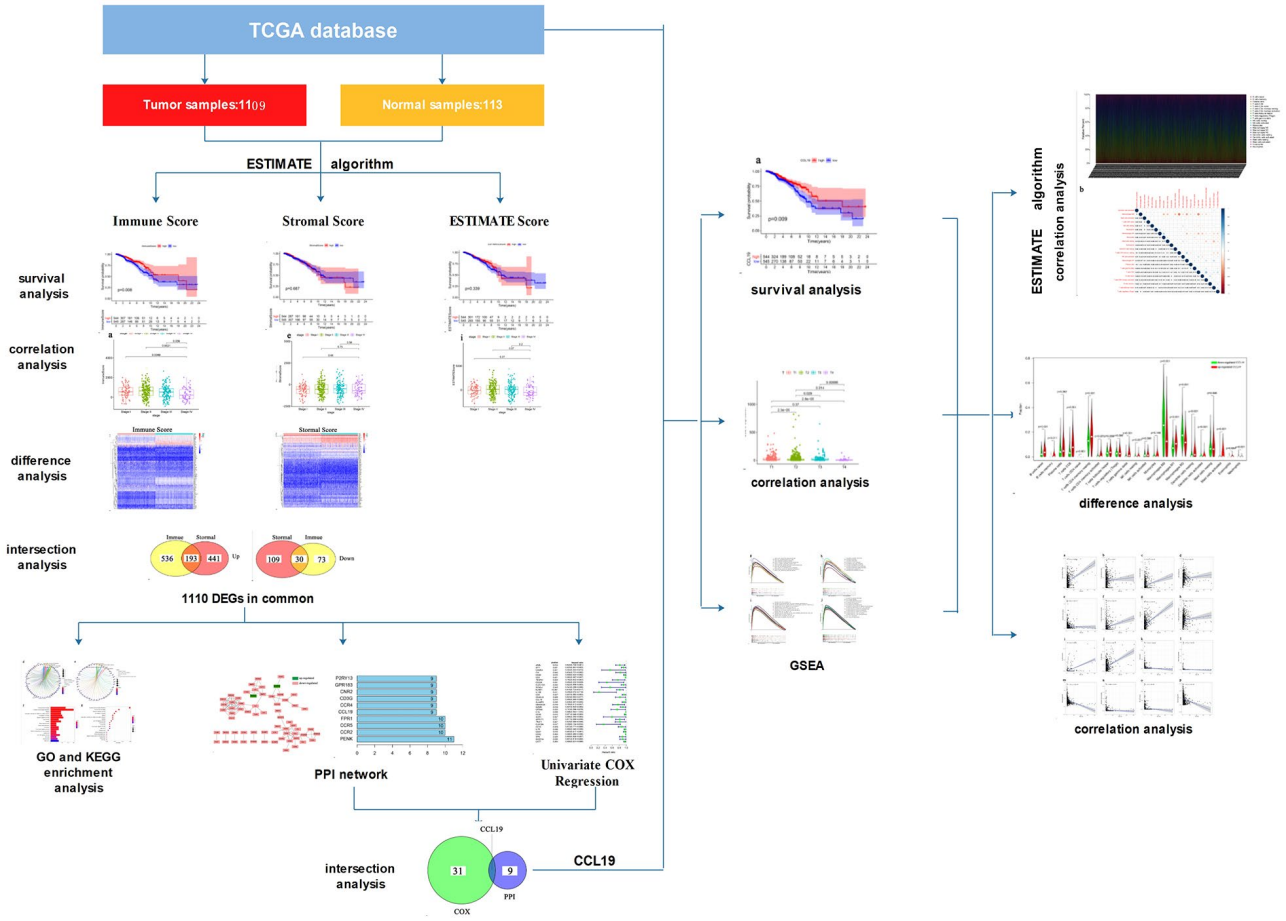
The analysis procedure of this study.

Firstly, we downloaded the transcriptome profiling and corresponding clinical materials of 1109 breast tumor samples and 113 normal breast samples from the TCGA database. Secondly, we evaluated the proportion of immune/stromal component in TME using ESTIMATE algorithm and the relationship between Immune/Stromal Score and survival rates or clinic–pathological characteristics of BC patients. Thirdly, a total of 223 DEGs were obtained based on the immune score and stromal score. GO and KEGG enrichment analysis and PPI network were performed to learn the specific biological functions of these DEGs. Univariate COX regression was used to assess the effects of these DEGs on BC patients’ survival. Finally, CCL19 came to our eyes. And we focused on the association between the expression of CCL19 and the survival or clinic–pathological characteristics of BC patients. In addition, GSEA was carried out to find the biological functions of CCL19. CIBERSORT algorithm was applied to evaluate the TIC profile and correlation analysis was used to evaluate the relationship between the expression of CCL19 and the proportion of TICs in TIME. The analysis procedure was shown in Figure 1.

### Immune component in TME was associated the survival of BC patients

**Figure 2:**
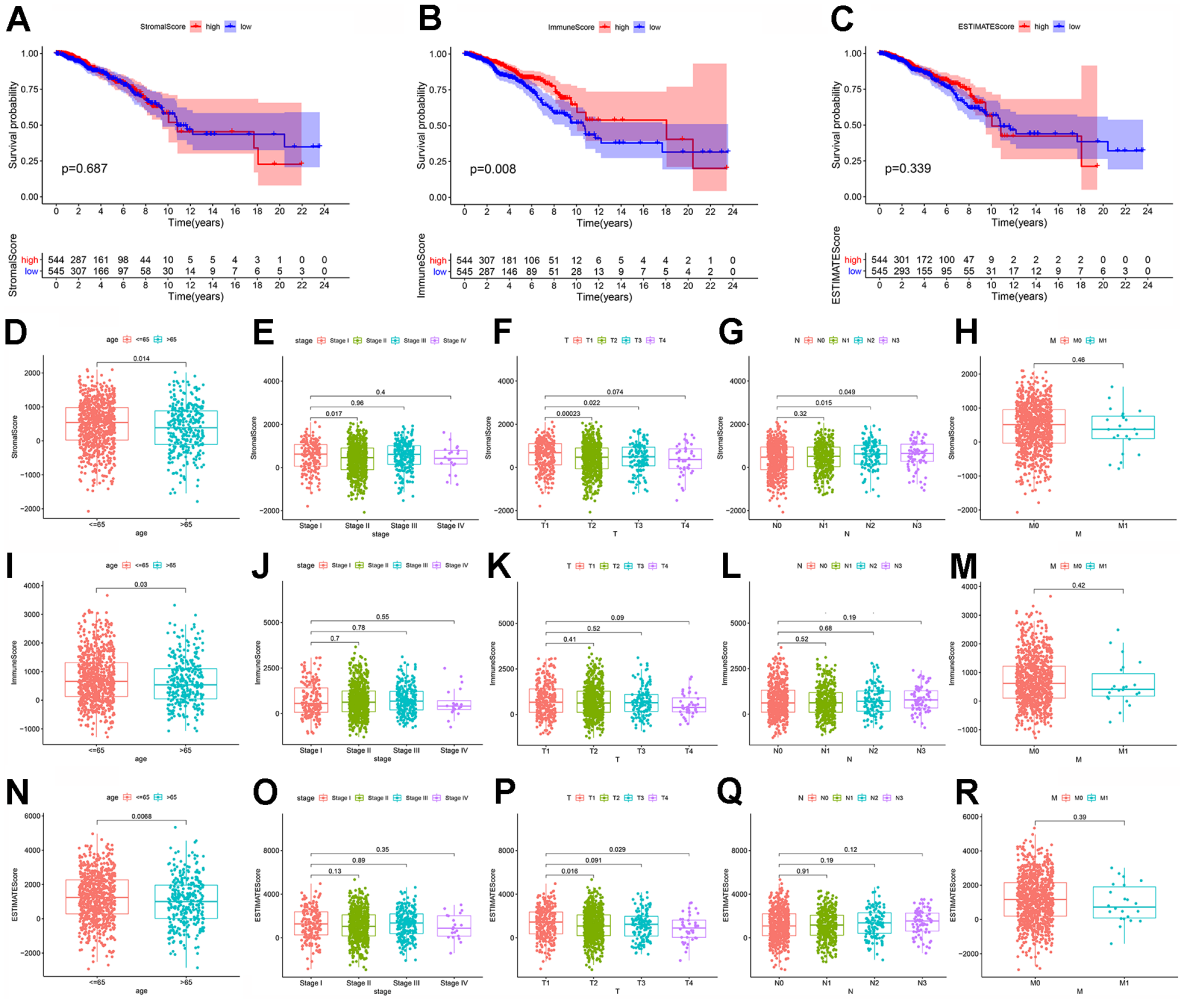
**The association of Immune/Stromal/ESTIMATE score and the survival or clinic-pathological characteristics of breast cancer patients.** (**A**–**C**) Kaplan–Meier survival analysis was applied to evaluate the associations between Immune/Stromal/ESTIMATE Score and the survival of breast cancer patients. Patients were divided into two groups, high score group and low score group, compared with the median. P-value and the number of samples in each group were displayed in the diagram; (**D**–**H**) The associations of Stromal Score with age, stage and TNM classification of breast cancer patients; (**I**–**M**) The associations of Immune Score with age, stage and TNM classification of breast cancer patients; (**N**–**R**) The associations of ESTIMATE Score with age, stage and TNM classification of breast cancer patients.

In order to explore the underlying relationship between the ratio of immune or stromal component in TME and the survival rate of BC patients, we first established the immune/stromal component evaluation system. The higher score reflected the larger amount of the immune or stromal component in TME. And then survival analysis was carried out by Kaplan–Meier plot and log-rank tests. The results showed that Immune Score was significantly associated with the survival rate of BC patients (Figure 2B; p=0.008), which meant that the higher Immune Score predicted the better survival. Yet, neither Stromal nor ESTIMATE Score was significantly related with the survival rate of BC patients (Figure 2A, 2C; p=0.687, 0.339). The above results implied that the immune component in TME might be more suitable for indicating the prognosis of BC patients.

### Both immune and stromal component were significantly associated with the specific clinic-pathological features of BC patients

To further determine the associations between scores and clinic-pathological features of BC patients, we analyzed the corresponding clinical materials (Supplementary Table 1) of BC patients, including the age, stages and TNM classification. First, we found that Stromal, Immune and ESTIMATE Score were significantly associated with the age of BC patients, which might indicate that the component of TME changed with age (Figure 2D, 2I, 2N; p=0.014, 0.03, 0.0068). For other clinic-pathological features, we found that there was significant difference in the Stromal Score in stage I compared with stage II (Figure 2E, p=0.017). There was also significant difference in the Stromal Score in stage T1 compared with stage T2 or T3, in stage N0 compared with stage N2 (Figure 2F, 2G, p=0.00023, 0.022, 0.015). However, for Immune Score, we were unable to find any difference in stages or TNM classifications (Figure 2J–2M). In addition, for ESTIMATE Score, there was also significant difference between T1 and T2 or T4 (Figure 2P, p=0.016, 0.029). Nonetheless, none of Immune, Stromal or ESTIMATE Score was related with the distant metastasis of BC (Figure 2H, 2M, 2F; p>0.05). From above, we concluded that the proportion of immune and stromal component might be associated with the early progression of BC, instead of the distant metastasis.

### DEGs were significantly related with immune-related activities

**Figure 3:**
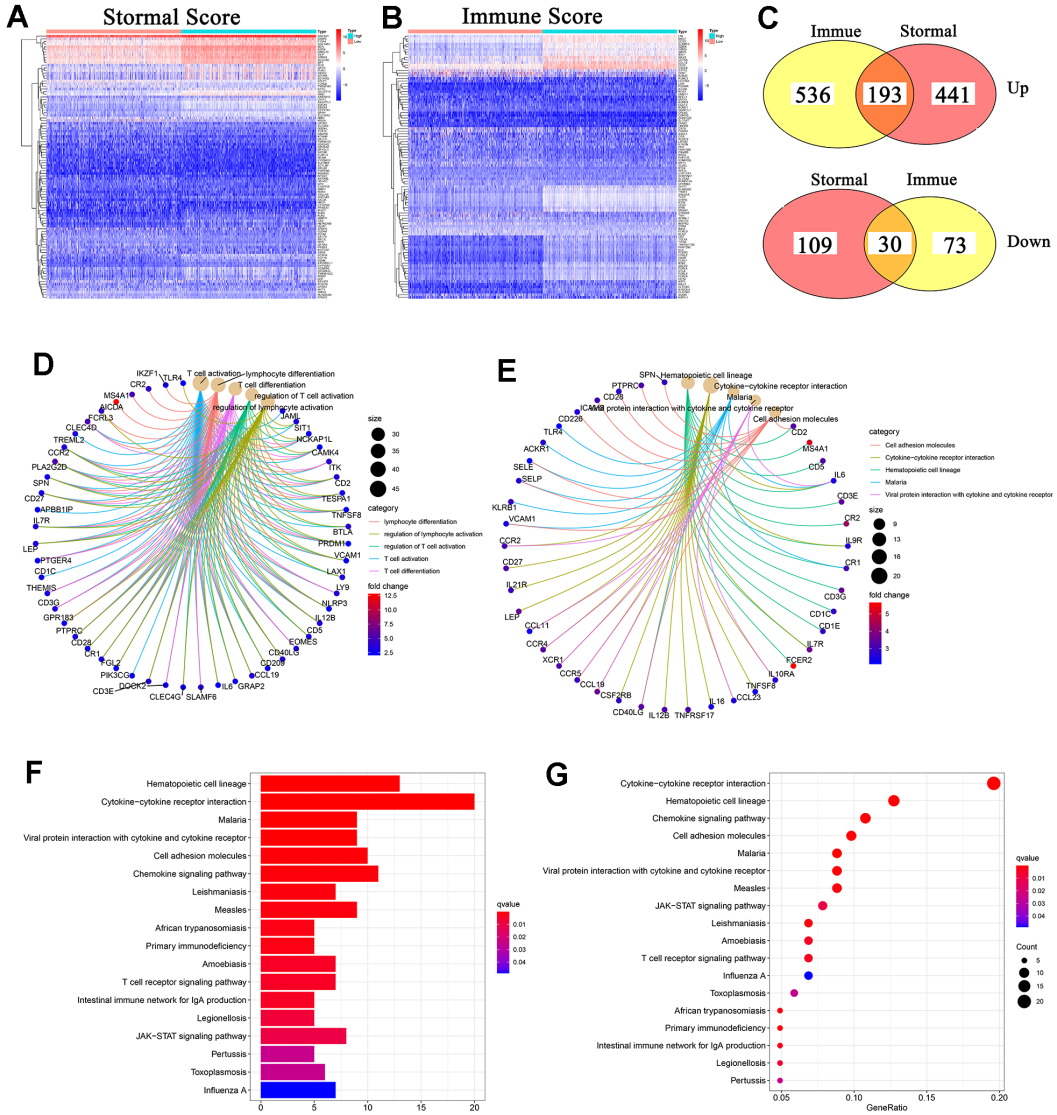
**Heatmaps, venn plots, GO and KEGG enrichment analyses.** (**A**, **B**) Heatmaps of DEGs obtained by comparison between high Stromal/Immune Score samples with low Stromal/Immune Score samples. p<0.05 and log2^FC^ >1 were set up to search the DEGs; (**C**) Venn plots displayed the commonly up-regulated or down-regulated DEGs shared by Immune Score and Stromal Score; (**D**) GO enrichment analysis of 223 DEGs, p<0.05 was considered to be statistically significant; (**E**–**G**) KEGG enrichment analysis of 223 DEGs, p<0.05 was considered to be statistically significant; BP: biological process; CC: cell component; MF: molecular function.

In order to obtain the DEGs profile in TME, we performed the difference analysis between the high Stromal/Immune Score group and the low Stromal/Immune Score group. Heat map was plotted to show the gene profile in TME (Figure 3A, 3B). In detail, there were a total of 773 DEGs obtained from the Stromal Score group (high Stromal Score vs. low Stromal Score), among which 634 DEGs were up-regulated and 139 DEGs were down-regulated (Figure 3A and Supplementary Table 2). Additionally, there were a total of 832 DEGs obtained from the Immune Score group (high Immune Score vs. low Immune Score), among which 729 DEGs were up-regulated and 103 DEGs were down-regulated (Figure 3B and Supplementary Table 3). Next, we conducted the intersection analysis to acquire the commonly up-regulated or down-regulated DEGs in Immune and Stromal Score groups. The Venn diagram showed that 193 DEGs were up-regulated and 30 DEGs were down-regulated in both Immune Score group and Stromal Score group (Figure 3C). Thus, these 223 DEGs (Supplementary Table 4) might take a great part in modulating the status of TME. As a result, GO and KEGG enrichment analyses were further performed to assess the detailed functions of these 223 DEGs. Go enrichment analysis indicated that 223 DEGs greatly participated in lymphocyte activation, T cell activation and T cell differentiation (Figure 3D). Similarly, KEGG enrichment analysis revealed that 223 DEGs were significantly associated with specific immune-related activities, such as cell adhesion molecules, cytokine-cytokine receptor interaction and chemokine signaling pathway (Figure 3E–3G). From above, these 223 DEGs seemed to be closely related with immune-related activities and might be a primary feature of TME in BC.

### PPI network and univariate COX regression analysis of DEGs

**Figure 4:**
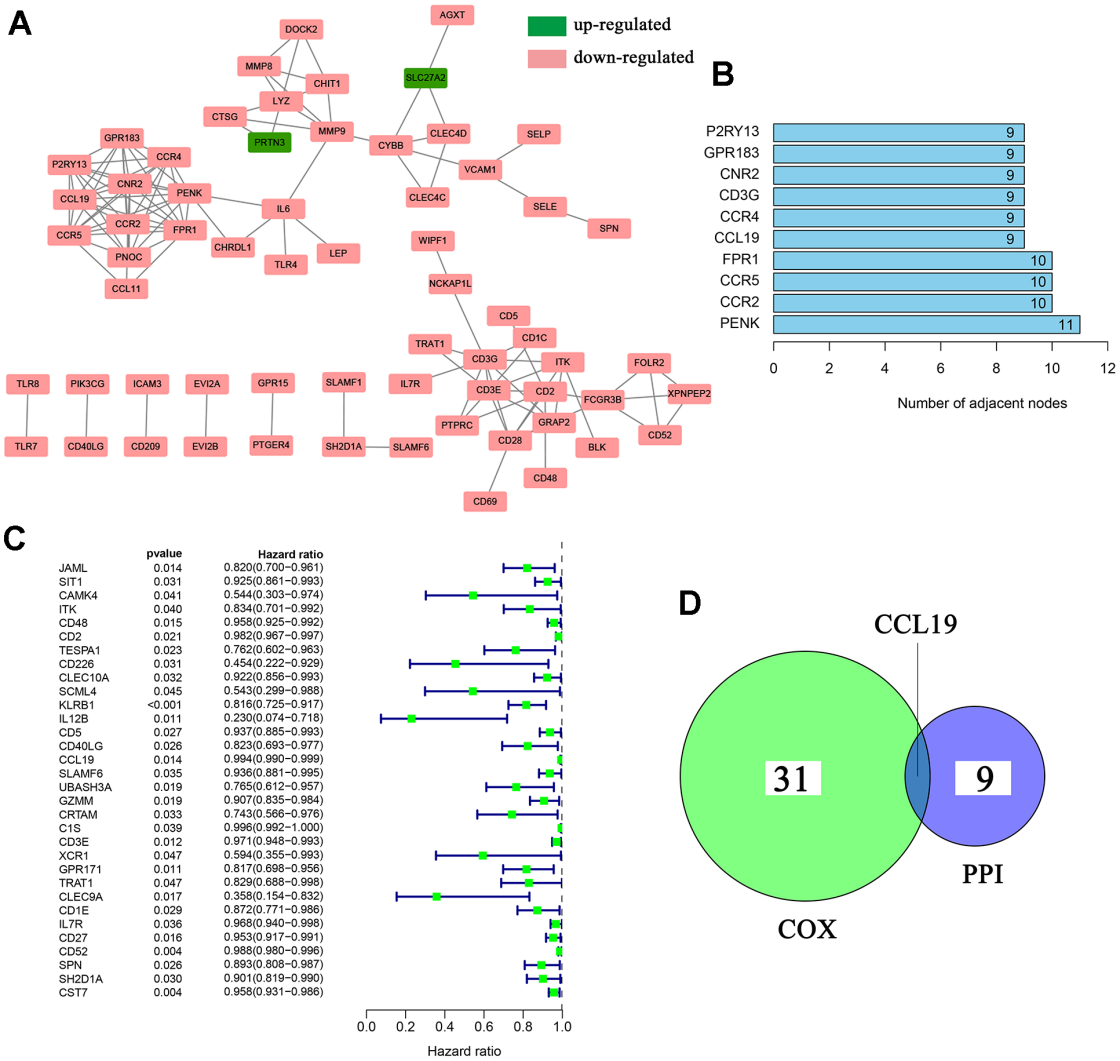
**PPI network construction and univariate COX regression analysis.** (**A**) PPI network was constructed with interaction confidence value >0.95; (**B**) The top 10 DEGs which shared the leading nodes in PPI network; (**C**) Univariate COX regression analysis of 223 DEGs. p<0.05 was considered to be statistically significant; (**D**) Venn plot displayed the DEGs which shared the top 10 leading nodes in PPI network and were significant related with patients’ survival. p<0.05 was considered to be statistically significant.

To learn more about the potential interaction mechanisms of these 223 DEGs, PPI network was first constructed with the help of STRING database and Cytoscape software. The specific interaction network of DEGs was showed in Figure 4A. The top 10 DEGs, which shared the leading nodes, were ranked in Figure 4B. We next conducted the univariate COX regression analysis to acquire the DEGs, which were significantly associated with BC patients’ survival. As a result, 32 DEGs were found to be closely related with the hazard ratio (HR) of BC patients’ survival (Figure 4C, p<0.05; Supplementary Table 5). Finally, the intersection analysis was used to find out the DEGs, which shared the leading nodes in PPI and were significantly associated with patients’ survival (Figure 4D). Luckily, C-C motif chemokine 19 (CCL19) emerged.

### CCL19 was significantly associated with the survival and specific clinic-pathological features of BC patients

**Figure 5:**
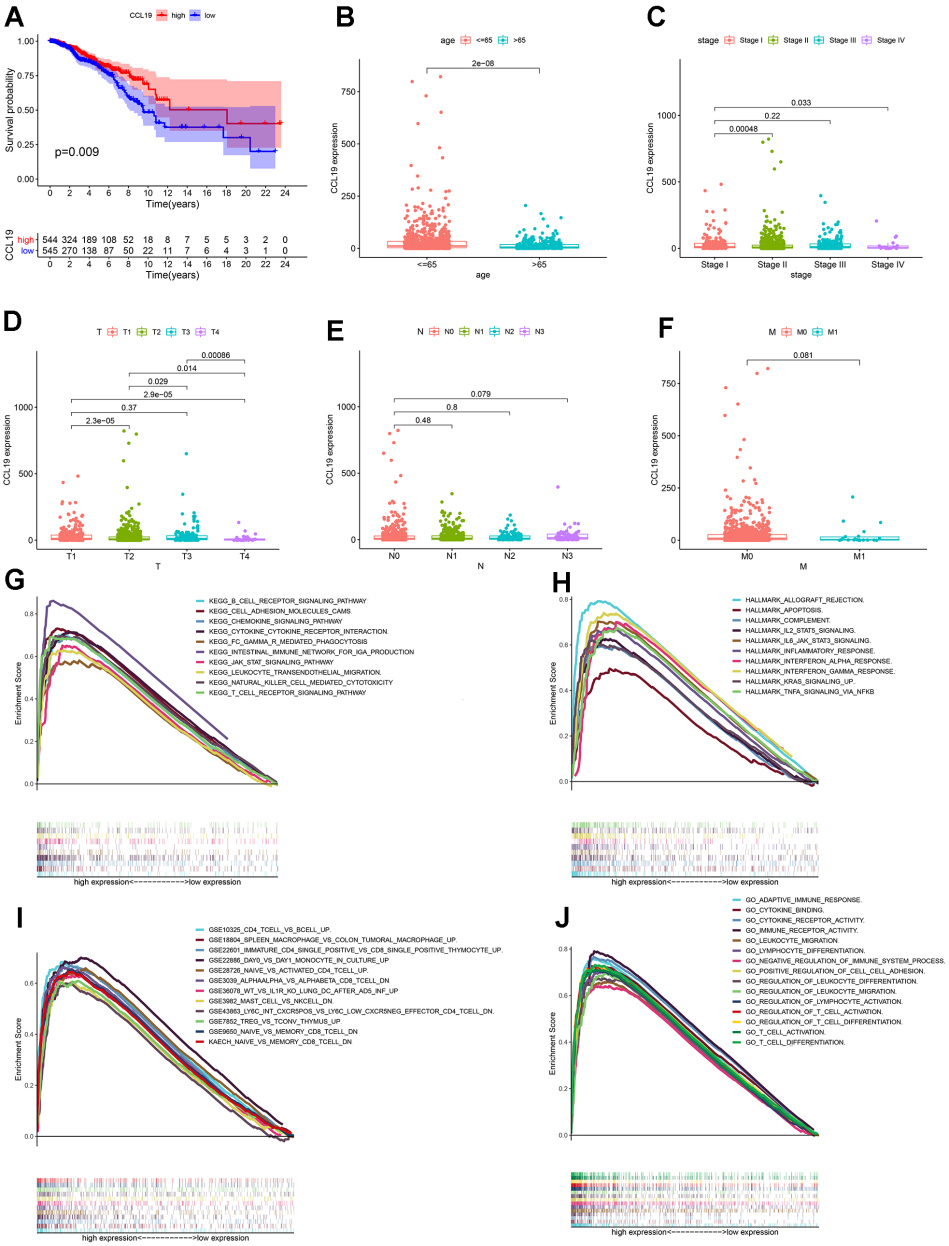
**The survival analysis, differential analysis and GSEA of CCL19.** (**A**) The survival analysis between the expression of CCL19 and the survival of breast cancer patients; patients were divided into two groups, high CCL19 expression group and low CCL19 expression group, compared with the median; (**B**–**F**) The associations between the expression of CCL19 with age, stage, and TNM classification of breast cancer patients; p value was displayed in the diagram; (**G**–**J**) GSEA for genes in high CCL19 expression group in different gene sets, p<0.05 and FDR<0.05 were considered to be statistically significant.

CCL19, a ligand of the chemokine receptor C–C chemokine receptor type 7 (CCR7), is often expressed in the T-cell zones, including lymph nodes and thymus [12]. Recently, researches revealed that the overexpression of CCL19 in serum was associated with the progression of melanoma [13, 14]. In addition, CCL19 functioned as an immune-modulator for colon cancer therapy by closely communicating with DCs, T cells and B cells, thus regulating the adaptive immune responses [15]. However, there are few researches focusing on the role of CCL19 in BC. In the present study, we collected the tissues of carcinoma *in situ* and infiltrating breast carcinoma, and examined the expression of CCL19 through IHC. The result showed that CCL19 was highly expressed in tumor than in mesenchyme (Supplementary Figure 1A, 1B). The survival analysis suggested that the expression of CCL19 was positively related with the survival of BC patients (Figure 5A, p=0.009). As for the clinic-pathological features, the expression of CCL19 was significantly associated with ages, stages, T classifications and N classifications of BC patients (Figure 5B–5E, p<0.05). However, it was not connected with M classification (Figure 5F, p>0.05), which was in accordance with the difference analysis between Score and clinic-pathological features (Figure 2H, 2M, 2F; p>0.05). From above, we might conclude that CCL19 was likely to be a prognostic factor and participate in the early progression of BC.

### CCL19 greatly participated in immune-related activities

Given that CCL19 was significantly associated with the survival and specific clinic-pathological features of BC patients, we further carried out GSEA to explore the underlying mechanisms of CCL19. First, BC samples were divided into two groups according to the expression of CCL19, including CCL19 up-regulated group and CCL19 down-regulated group, compared with the median. For C2 collection, the KEGG gene sets database, genes in CCL19 up-regulated group were obviously enriched in immune-related activities, including B cell receptor signaling pathway, cell adhesion molecules cams, chemokine signaling pathway and so on (Figure 5G). For hallmark gene sets, genes in CCL19 up-regulated group were primarily enriched in allograft rejection, complement, IL2 stat5 signaling and so on (Figure 5H). For C7 collection defined by MSigDB, the immunologic gene sets, a variety of immune functional gene sets were enriched in the high CCL19 expression group (Figure 5I). At last, for C5 collection, the gene ontology sets, the genes in CCL19 up-regulated group were also enriched in immune-related activities, such as adaptive immune response, cytokine binding, cytokine receptor activity and so on (Figure 5J). Thus, we concluded that CCL19 greatly participated in the immune-related activities in BC patients and might be a modulator of TME.

### CCL19 was associated with TICs in TIME

**Figure 6:**
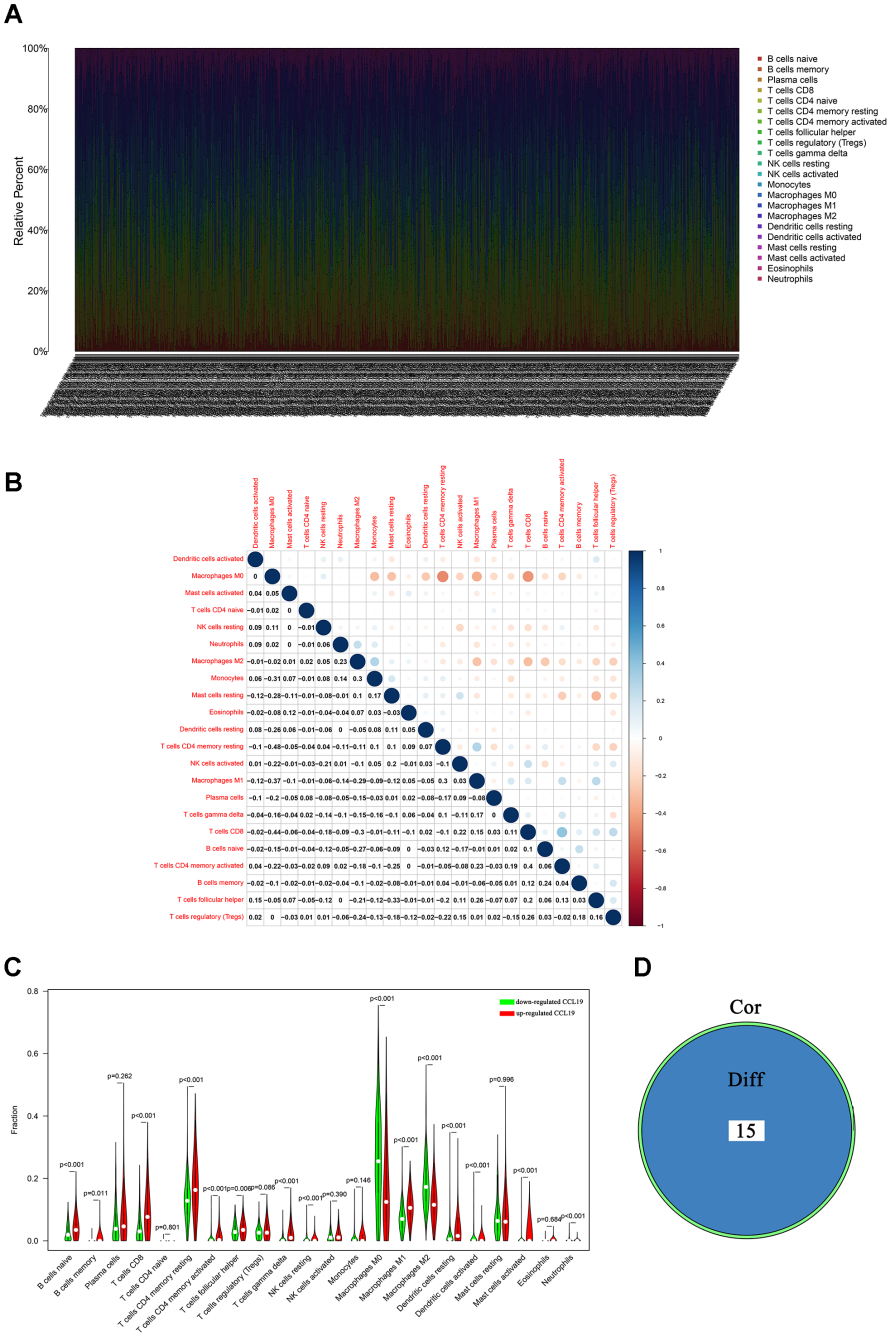
**TIC profile and differential analysis.** (**A**) Barplot showed the proportion of 22 TICs in breast cancer samples; (**B**) The diagram showed the associations between 22 TICs; each spot indicated the p value of the association between two TICs; (**C**) Violin plot showed the correlation between 22 TICs and the expression of CCL19; p<0.05 was considered to be statistically significant; (**D**) Venn plot indicated there were 15 TICs shared by the difference test and correlation test showed in violin plots (**C**) and scatter plots (Figure 7), respectively.

**Figure 7:**
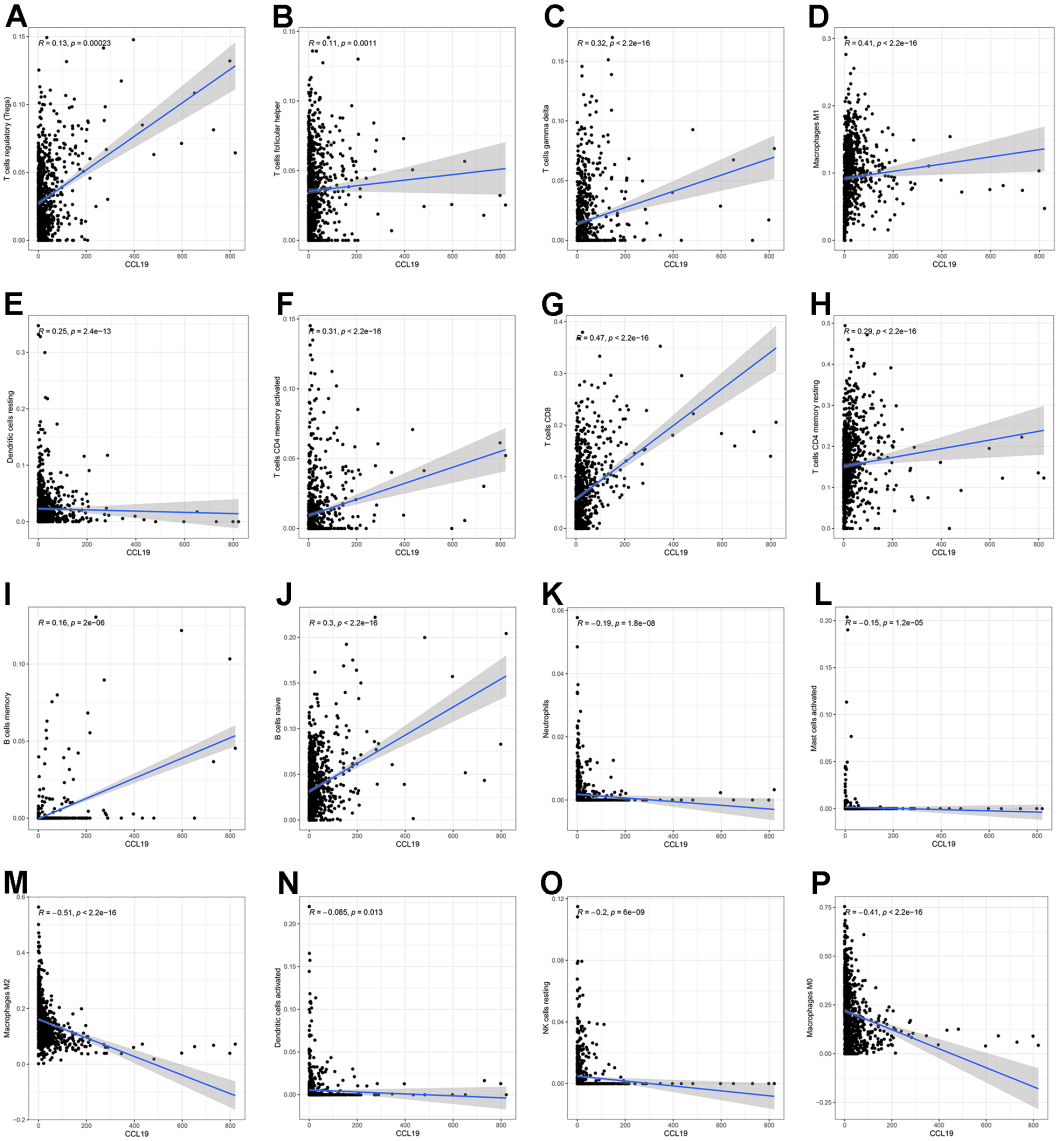
**Scatter plots indicated the correlation between 16 TICs and the expression of CCL19, p<0.05 was considered to be statistically significant.** (**A**) The correlation between Tregs and the expression of CCL19; (**B**) The correlation between T cells follicular helper and the expression of CCL19; (**C**) The correlation between T cells gamma delta and the expression of CCL19; (**D**) The correlation between Macrophages M1 and the expression of CCL19; (**E**) The correlation between Dendritic cells resting and the expression of CCL19; (**F**) The correlation between T cells CD4 memory activated and the expression of CCL19; (**G**) The correlation between T cells CD8 and the expression of CCL19; (**H**) The correlation between T cells CD4 memory resting and the expression of CCL19; (**I**) The correlation between B cells memory and the expression of CCL19; (**J**) The correlation between B cells naive and the expression of CCL19; (**K**) The correlation between Neutrophils and the expression of CCL19; (**L**) The correlation between Mast cells activated and the expression of CCL19; (**M**) The correlation between Macrophages M2 and the expression of CCL19; (**N**) The correlation between Dendritic cells activated and the expression of CCL19; (**O**) The correlation between NK cells resting and the expression of CCL19; (**P**) The correlation between Macrophages M0 and the expression of CCL19.

Since we had found that CCL19 greatly participated in immune-related activities and might play an important role in the regulation of TME, we speculated that there were underlying connections between CCL19 and TIME. CIBERSORT algorithm was first applied to evaluate the proportion of TICs in BC samples (Figure 6A). The associations between TICs were displayed in Figure 6B. The difference analysis was performed to verify the associations between the expression of CCL19 and 22 TICs. The violin plot suggested that 15 TICs were significantly associated with the expression of CCL19 in BC samples (Figure 6C and Supplementary Table 6). Next, the correlation test showed that 10 TICs were positively related with the expression of CCL19 (Figure 7A–7J and Supplementary Table 6), and 6 TICs were negatively related with the expression of CCL19 (Figure 7K–7P and Supplementary Table 6). Finally, the intersection analysis between the difference test and correlation test revealed that 15 TICs were significantly associated with the expression level of CCL19 (Figure 6D and Supplementary Table 6). As a consequence, CCL19 was significantly associated with multiple TICs in TIME and might play an important role in modulating the TIME of BC through communicating with various TICs.

## DISCUSSION

In our study, we focused on the TME-related genes that were significantly associated with the survival and the clinic-pathological features of BC. Firstly, we downloaded the transcriptome profiling and the clinical data of 1109 breast tumor samples and 113 normal breast samples from the TCGA database. Secondly, we applied the ESTIMATE algorithm to calculate the ratio of immune/stromal component in TME and found out that immune/stromal component in TME was closely related with the survival rate and clinic–pathological characteristics of BC patients. Thirdly, in order to further explore the gene expressing profiling regarding the TME, 223 DEGs were obtained based on the Immune Score and Stromal Score. GO and KEGG enrichment analysis indicated that a total of 223 DEGs were primarily enriched in immune-related activities, such as lymphocyte activation, T cell activation, T cell differentiation, cytokine-cytokine receptor interaction, chemokine signaling pathway and so on. Finally, CCL19 was acquired by intersection analysis between PPI network and univariate COX regression analysis. CCL19 shared the leading nodes in PPI network and was significantly associated with the HR of BC patients’ survival. And then survival analysis and differential analysis revealed that the overexpression of CCL19 predicted the better survival and clinic–pathological characteristics of BC patients. Additionally, GSEA revealed that genes in CCL19 up-regulated group were mainly enriched in immune-related activities, such as B cell receptor signaling pathway, chemokine signaling pathway, allograft rejection, cytokine receptor activity and so on. Furthermore, CCL19 was closely associated with a variety of TICs in TIME and might play an important role in modulating TIME through communicating with multiple TICs.

CCL19, a ligand of CCR7, was overexpressed in the T-cell zones and functioned as an essential modulator of immune responses through affecting the migration of DCs and T cells into the secondary lymphatic tissues and thereby influencing cell homing and adaptive immunity [16–20]. Recently, H. W. Cheng, et al. [21] declared that CCL19-expressing fibroblastic stromal cells (FSCs) accelerated the intratumoral accumulation of CD8+ T cells and inhibited the tumor growth in non-small cell lung cancer. CCL19, along with CCR7, acted as clinically prognostic factors in lung adenocarcinoma [22]. In addition, CCL19-expressing chimeric antigen receptor (CAR) T cells promoted the infiltration of DCs and T cells into tumor tissues and exerted synergistic anti-tumor activity with recipient immune cells [23]. In other solid tumors, such as ovarian and colon cancers, CCL19-expressing embryonic endothelial progenitor cells and mesenchymal stem cells functioned to activate the local TIME and led to a good therapeutic effect [24, 25]. On the other hand, immune cells, such as DCs, can also release CCL19 during the migration, to maintain immune surveillance and tissue homeostasis [26, 27]. However, there are few researches exploring the role of CCL19 in BC and the correlation between the expression of CCL19 and other TICs. H. Hwang, et al. [28] indicated that TNBC cells advanced the migration of DCs toward CCL19 through releasing soluble factors and activating JNK/c-Jun signaling pathway.

In our study, we applied multiple analytical methods to evaluate the functions of DEGs regarding stromal and immune component in TME of BC samples from TCGA and finally found out the most significant gene, CCL19. Further analysis between the expression of CCL19 and the survival of patients or TICs in TIME suggested that CCL19 was not only significantly associated with the survival and clinic–pathological characteristics of BC patients, but also participated in the regulation of TIME partly through closely communicating with multiple TICs, including macrophages, T cells, B cells, NK cells, DCs, mast cells and neutrophils. Previous studies indicated that CCL19 greatly participated in cancer cell proliferation, angiogenesis, migration, invasion and immune cell infiltration [23] in multiple cancers, including gastric cancer [29], colorectal cancer [30], non-small cell lung cancer [31, 32], lymphoma [33] and melanoma [13]. These biological functions of CCL19 were involved in the activation of CCR7/absent in melanoma 2 (AIM2) signaling pathway [29], interaction with miRNAs [30], the migration and maturation of DC [34] and migration of NK cells [35]. Our studies provided a new point of view that CCL19 might not only be connected with the migration and maturation of DCs or NK cells, but also associated with macrophages, T cells, B cells, mast cells and neutrophils. And further researches were needed to clarify the specific associations between CCL19 and multiple TICs in BC.

## CONCLUSIONS

We applied ESTIMATE algorithm to assess the immune and stromal component in TME of BC samples downloaded from TCGA database and identified TME-associated DEGs through multiple analytical methods. The results indicated that CCL19 was a potential prognostic biomarker for BC patients. More interestingly, CCL19 might function as a modulator of BC TIME and is significantly connected with various TICs. Therefore, further investigation should be carried out to clarify the accuracy of the combined analysis of CCL19 expression, explore the role of CCL19 in TIME of BC and focus more on the correlation between CCL19 and these immune cells instead of just aiming at DCs.

## MATERIALS AND METHODS

### Data

Transcriptome profiling and the corresponding clinical materials of BC samples and normal breast samples were downloaded from TCGA database (https://portal.gdc.cancer.gov/). According to the statistics, there were 1109 tumor samples and 113 normal samples in total.

### The calculation of immune score, stromal score, and ESTIMATE score

The ratio of immune or stromal component in TME of each tumor sample was analyzed in R language (version 3.6.3) with the help of ESTIMATE algorithm. Immune Score represented the proportion of immune ingredient in TME. Stromal Score represented the proportion of stromal ingredient in TME. ESTIMATE Score represented both the immune and stromal ingredient in TME.

### Survival analysis

Survival analysis was carried out with the help of survival and survminer packages in R. Kaplan–Meier plot and log-rank tests were performed to assess the associations between Immune/Stromal/ESTIMATE Score and the survival rate of BC patients. Survival analysis was also conducted to evaluate the relationship between the expression of CCL19 and the survival rate of BC patients. Univariate COX regression analysis was carried out by package survival in R to assess the influence of CCL19 on the survival of BC patients. P < 0.05 was considered to be statistically significant.

### Differential analysis between immune/stromal/ESTIMATE score and clinic-pathological features

The differential analysis was carried out to evaluate the relationships between Immune/Stromal/ESTIMATE Score and clinic-pathological features, including stages and TNM classification. The differential analysis was also conducted to assess the association between the expression of CCL19 and clinic-pathological features. Wilcoxon rank sum and Kruskal–Wallis rank sum test were used for comparison.

### Confirmation of DEGs regarding immune or stromal score

1109 tumor samples were divided into the high-score group and the low-score group compared with the median and based on the above calculation of Immune Score and Stromal Score. Package limma in R was used for data analysis. A fold change (FC, log2 ^(high-score /low-score)^) > 1 and false discovery rate (FDR) <0.05 were set up to search the DEGs. Heatmaps and venn diagram of DEGs were performed by using pheatmap package and VennDiagram package in R, respectively.

### Gene Ontology (GO) and Kyoto Encyclopedia of Genes and Genomes (KEGG) enrichment analysis

ClusterProfiler, enrichplot, and ggplot2 packages in R were used for the GO and KEGG enrichment analysis of 223 DEGs. P< 0.05 was considered to be statistically significant.

### Protein–protein interaction (PPI) network and gene set enrichment analysis (GSEA)

PPI network of 223 DEGs was downloaded from the Search Tool for Retrieval of Interacting Genes/Proteins (STRING) database (version 11.0) and further reconstructed in Cytoscape (3.6.1). The confidence of interactive relationship of nodes was greater than 0.95. GSEA (4.1.0) was used to evaluate the functional profile of CCL19. P< 0.05 was considered to be statistically significant.

### Tumor-infiltrating immune cell (TIC) profile

CIBERSORT algorithm was applied to evaluate the TIC profile in breast tumor samples.

### Immunohistochemistry (IHC)

Slides (4μm) of formalin-fixed paraffin-embedded tissue sections were incubated with CCL19 (1:300; Immunoway) antibody. Immunohistochemistry was carried out with LSAB2 Kit (Dako, Carpinteria, CA), color development with 3-3diaminobenzidine, and counterstaining with hematoxylin.

### Availability of data and material

The datasets generated and analyzed in this research are available in TCGA database (https://portal.gdc.cancer.gov).

## Supplementary Material

Supplementary Figure 1

Supplementary Table 1

Supplementary Table 2

Supplementary Table 3

Supplementary Table 4

Supplementary Tables 5 and 6
