# Supplementary material for: CCL19 has potential to be a potential prognostic biomarker and a modulator of tumor immune microenvironment (TIME) of breast cancer: a comprehensive analysis based on TCGA database
Source: Aging (Albany NY). 2022 May 12;14(9):4158–75. doi: 10.18632/aging.204081 (PMC9134962; doi:10.18632/aging.204081)
Supplement: Supplementary Figure 1 [file aging-14-204081-s001.pdf]

SUPPLEMENTARY FIGURE

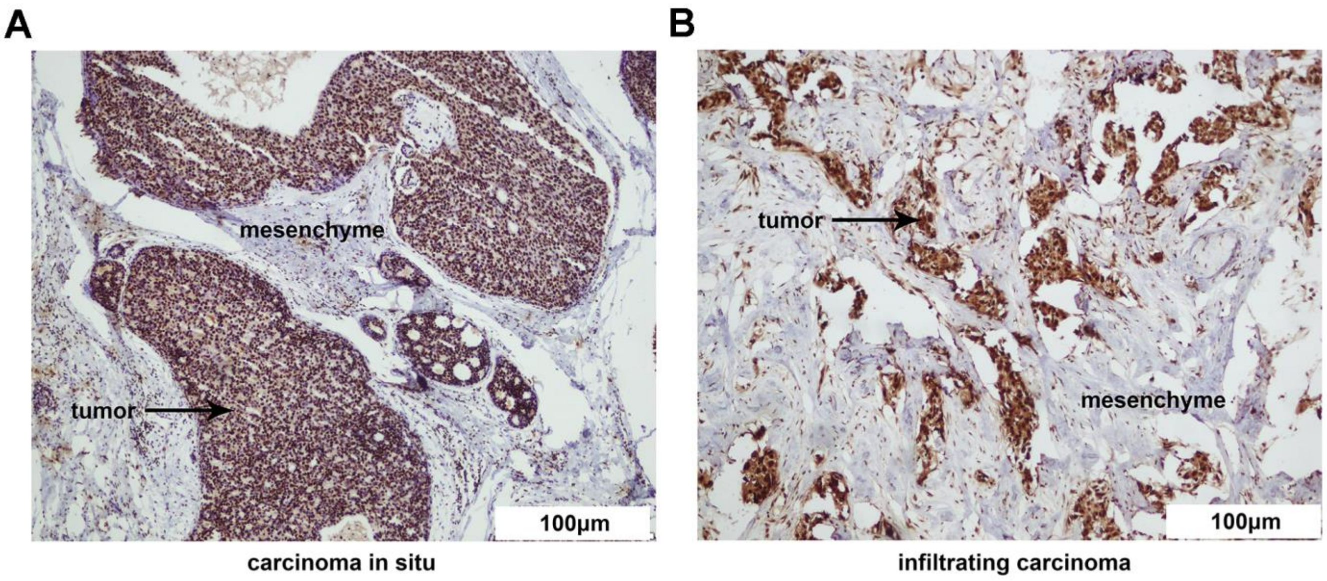

Supplementary Figure 1. The expression of CCL19 in breast cancer tissues. (**A**) The expression of CCL19 in carcinoma in situ of breast cancer. (**B**) The expression of CCL19 in carcinoma in infiltrating breast carcinoma.
