# Supplementary material for: CCL19 has potential to be a potential prognostic biomarker and a modulator of tumor immune microenvironment (TIME) of breast cancer: a comprehensive analysis based on TCGA database
Source: Aging (Albany NY). 2022 May 12;14(9):4158–75. doi: 10.18632/aging.204081 (PMC9134962; doi:10.18632/aging.204081)
Supplement: Supplementary Table 1 [file aging-14-204081-s002.pdf]

SUPPLEMENTARY TABLE

Supplementary Table 1. The clinic–pathological characteristics of breast cancer samples from TCGA database.

Clinic–pathological characteristics	Classification	Total	Percentage (%)
Age	<=65	776	70.74
	>65	321	29.26
Gender	Male	12	1.09
	Female	1085	98.91
Stage	I	183	16.68
	II	621	56.61
	III	249	22.70
	IV	20	1.82
	Unknow	24	2.19
T Classification	T1	281	25.62
	T2	635	57.89
	T3	138	12.58
	T4	40	3.65
	Unknow	3	0.27
N Classification	N0	516	47.04
	N1	364	33.18
	N2	120	10.94
	N3	77	7.02
	Unknow	20	1.82
M Classification	M0	912	83.14
	M1	22	2.01
	Unknow	163	14.86
