# Supplementary material for: CCL19 has potential to be a potential prognostic biomarker and a modulator of tumor immune microenvironment (TIME) of breast cancer: a comprehensive analysis based on TCGA database
Source: Aging (Albany NY). 2022 May 12;14(9):4158–75. doi: 10.18632/aging.204081 (PMC9134962; doi:10.18632/aging.204081)
Supplement: Supplementary Table 2 [file aging-14-204081-s003.docx]

**Supplementary Table 2. Differentially expressed genes (DEGs) regarding stromal scores.**

| **Gene** | **conMean** | **treatMean** | **logFC** | **pValue** | **FDR** |
| --- | --- | --- | --- | --- | --- |
| CLDN6 | 2.490514 | 0.418272 | -2.57393 | 0.0349944 | 0.044671 |
| S1PR1 | 5.047346 | 10.95065 | 1.11742 | 1.91E-63 | 5.51E-62 |
| JAML | 0.736753 | 1.601144 | 1.11985 | 1.36E-47 | 2.11E-46 |
| SIT1 | 1.474316 | 2.979621 | 1.015083 | 1.96E-32 | 1.71E-31 |
| MMP8 | 0.083356 | 0.177601 | 1.091286 | 2.12E-11 | 6.33E-11 |
| MS4A2 | 0.485632 | 1.204465 | 1.310457 | 2.78E-49 | 4.63E-48 |
| GXYLT2 | 2.87925 | 8.895261 | 1.627344 | 7.36E-102 | 1.10E-99 |
| NCKAP1L | 2.285361 | 4.832886 | 1.080463 | 7.23E-68 | 2.46E-66 |
| CMA1 | 0.350966 | 1.037837 | 1.564177 | 3.45E-33 | 3.10E-32 |
| SLIT2 | 1.175344 | 2.826223 | 1.265792 | 6.68E-95 | 6.84E-93 |
| EVI2B | 5.713191 | 12.29529 | 1.105737 | 6.02E-69 | 2.15E-67 |
| CAMK4 | 0.249985 | 0.500925 | 1.002753 | 3.96E-68 | 1.36E-66 |
| IL10RA | 2.845422 | 5.701897 | 1.002799 | 4.70E-54 | 9.49E-53 |
| GREM1 | 2.164901 | 5.517764 | 1.349783 | 7.10E-65 | 2.14E-63 |
| C3orf80 | 1.218883 | 2.470551 | 1.019273 | 1.69E-80 | 9.22E-79 |
| REEP2 | 2.251718 | 1.108416 | -1.02253 | 9.60E-09 | 2.34E-08 |
| TLX1 | 0.895707 | 0.383842 | -1.22251 | 7.20E-08 | 1.63E-07 |
| RASL10B | 1.908301 | 0.911734 | -1.0656 | 0.0001785 | 0.0002983 |
| DACT3 | 0.723103 | 1.53262 | 1.083726 | 4.26E-86 | 3.04E-84 |
| PODNL1 | 1.819227 | 3.908972 | 1.103464 | 3.04E-71 | 1.17E-69 |
| MRAP | 0.138713 | 0.512035 | 1.884142 | 4.77E-20 | 2.44E-19 |
| EGFL6 | 1.418132 | 2.954817 | 1.059077 | 1.18E-29 | 9.36E-29 |
| BICC1 | 1.826319 | 4.616516 | 1.337866 | 7.02E-117 | 2.58E-114 |
| JCHAIN | 64.42008 | 165.0954 | 1.357717 | 5.13E-33 | 4.59E-32 |
| ITK | 0.488371 | 1.151007 | 1.236846 | 5.72E-40 | 6.52E-39 |
| CNRIP1 | 1.471027 | 3.426925 | 1.22009 | 7.63E-140 | 3.47E-136 |
| GNG3 | 1.309552 | 0.441156 | -1.56971 | 2.50E-06 | 4.95E-06 |
| ADAMTS12 | 2.911638 | 6.784737 | 1.220462 | 1.52E-75 | 7.10E-74 |
| GLI1 | 0.236574 | 0.552597 | 1.223937 | 1.83E-40 | 2.11E-39 |
| CD48 | 3.616167 | 7.276844 | 1.008852 | 9.18E-45 | 1.27E-43 |
| TIMP2 | 48.71738 | 106.662 | 1.130537 | 2.11E-113 | 6.53E-111 |
| CORIN | 0.356793 | 1.050333 | 1.557687 | 2.20E-62 | 6.03E-61 |
| CD2 | 7.141867 | 14.3213 | 1.003789 | 2.60E-33 | 2.35E-32 |
| SSC5D | 2.703992 | 5.611953 | 1.053412 | 7.75E-67 | 2.52E-65 |
| NAP1L3 | 0.835755 | 1.947926 | 1.220787 | 2.39E-99 | 3.07E-97 |
| COX6A2 | 0.384532 | 0.173174 | -1.15088 | 0.0113779 | 0.0154384 |
| DAB2 | 6.949688 | 14.22962 | 1.033877 | 1.88E-125 | 1.60E-122 |
| CPZ | 0.138666 | 0.376595 | 1.441404 | 8.49E-72 | 3.38E-70 |
| TESPA1 | 0.470479 | 1.03122 | 1.13215 | 5.44E-46 | 7.89E-45 |
| C6 | 0.239169 | 0.693647 | 1.536173 | 4.33E-25 | 2.83E-24 |
| FAM110D | 0.960648 | 1.926526 | 1.003922 | 1.49E-25 | 9.97E-25 |
| HMCN1 | 1.184624 | 3.639473 | 1.619301 | 3.44E-100 | 4.79E-98 |
| KLHL6 | 0.614336 | 1.303927 | 1.085762 | 2.85E-59 | 6.88E-58 |
| ANK2 | 0.482537 | 1.145775 | 1.247611 | 6.00E-76 | 2.85E-74 |
| SCARA5 | 0.383904 | 1.275629 | 1.732391 | 9.95E-31 | 8.23E-30 |
| PCSK5 | 0.503672 | 1.17083 | 1.216975 | 5.80E-98 | 7.11E-96 |
| PRRX1 | 7.867329 | 19.97229 | 1.344054 | 9.73E-119 | 4.02E-116 |
| FIBIN | 6.083358 | 16.70882 | 1.45767 | 4.13E-110 | 9.22E-108 |
| MFAP4 | 12.65451 | 41.02993 | 1.697025 | 3.29E-78 | 1.68E-76 |
| VEGFD | 0.341888 | 0.760564 | 1.153546 | 8.12E-15 | 3.01E-14 |
| COL11A1 | 13.26199 | 41.55792 | 1.647826 | 3.40E-45 | 4.79E-44 |
| COL12A1 | 28.2737 | 73.85263 | 1.385189 | 3.36E-76 | 1.61E-74 |
| LUM | 116.458 | 369.8602 | 1.66717 | 2.00E-133 | 3.89E-130 |
| COL14A1 | 5.733553 | 21.04791 | 1.876176 | 1.61E-85 | 1.12E-83 |
| GLI2 | 0.389692 | 0.909664 | 1.223001 | 8.00E-91 | 6.81E-89 |
| CD226 | 0.177695 | 0.369342 | 1.055552 | 2.47E-45 | 3.50E-44 |
| PHGDH | 23.26334 | 10.09081 | -1.20502 | 1.89E-08 | 4.48E-08 |
| RUNDC3A | 1.787187 | 0.35446 | -2.33399 | 1.69E-05 | 3.09E-05 |
| ZEB1 | 2.566348 | 6.149591 | 1.260773 | 7.93E-115 | 2.70E-112 |
| WDFY4 | 0.697222 | 1.507714 | 1.112672 | 7.06E-59 | 1.68E-57 |
| KCTD12 | 10.2089 | 22.82465 | 1.160766 | 1.55E-114 | 5.04E-112 |
| MMRN1 | 0.580648 | 1.770421 | 1.608357 | 1.91E-43 | 2.52E-42 |
| ATP6V0D2 | 0.363835 | 0.894396 | 1.297629 | 6.80E-25 | 4.40E-24 |
| HTR2B | 0.462158 | 1.187842 | 1.361885 | 2.74E-74 | 1.21E-72 |
| FCGR3B | 0.367234 | 0.880757 | 1.262046 | 3.19E-09 | 8.07E-09 |
| ASPN | 18.67568 | 82.83136 | 2.149017 | 9.11E-95 | 9.26E-93 |
| ADAM33 | 0.446587 | 1.310089 | 1.552651 | 3.96E-66 | 1.24E-64 |
| LRRN4CL | 0.44928 | 1.351663 | 1.589049 | 2.35E-75 | 1.09E-73 |
| MAB21L1 | 0.266006 | 0.637927 | 1.261932 | 1.41E-66 | 4.57E-65 |
| MAP1LC3C | 0.213661 | 0.66861 | 1.645843 | 2.21E-58 | 5.20E-57 |
| MYT1 | 1.091271 | 0.285779 | -1.93304 | 6.87E-06 | 1.31E-05 |
| LPO | 0.189825 | 0.051867 | -1.87178 | 0.006447 | 0.0090061 |
| ABCB5 | 0.030092 | 0.081429 | 1.436153 | 3.12E-35 | 3.04E-34 |
| ANTXRL | 0.190603 | 0.058344 | -1.70791 | 0.0203617 | 0.026767 |
| ATOH8 | 0.264914 | 0.625969 | 1.240568 | 3.33E-32 | 2.89E-31 |
| LYVE1 | 0.546137 | 1.495497 | 1.453289 | 4.50E-44 | 6.10E-43 |
| CYP11A1 | 0.225983 | 2.209866 | 3.289673 | 3.25E-52 | 6.13E-51 |
| LRRTM2 | 0.106772 | 0.254776 | 1.254695 | 2.31E-42 | 2.90E-41 |
| ZNF831 | 0.146857 | 0.357999 | 1.285548 | 1.38E-38 | 1.51E-37 |
| SLC22A3 | 0.393031 | 0.789853 | 1.00694 | 1.02E-47 | 1.60E-46 |
| ADAMTS5 | 1.067541 | 2.197814 | 1.041778 | 1.91E-73 | 8.19E-72 |
| CBLN4 | 0.23621 | 0.493444 | 1.062817 | 1.10E-29 | 8.71E-29 |
| ADAMTS2 | 6.666031 | 17.86006 | 1.421837 | 6.28E-110 | 1.38E-107 |
| TMEM145 | 2.703915 | 0.913958 | -1.56485 | 5.23E-11 | 1.51E-10 |
| ACKR4 | 0.382922 | 1.035771 | 1.435583 | 9.08E-78 | 4.57E-76 |
| TGFBR2 | 13.57461 | 32.94206 | 1.27902 | 4.96E-121 | 2.55E-118 |
| 3-Sep | 5.251818 | 2.336258 | -1.16862 | 3.21E-17 | 1.40E-16 |
| ABI3BP | 1.470986 | 3.553804 | 1.27258 | 4.15E-72 | 1.69E-70 |
| GRIN2C | 0.565409 | 0.245459 | -1.20381 | 8.39E-05 | 0.0001449 |
| TNFSF8 | 0.690438 | 1.529656 | 1.147623 | 1.13E-71 | 4.48E-70 |
| CYS1 | 1.208579 | 3.059392 | 1.339933 | 4.51E-90 | 3.73E-88 |
| HPDL | 2.269322 | 1.039371 | -1.12655 | 4.48E-05 | 7.92E-05 |
| SCARF2 | 2.56225 | 5.517495 | 1.106602 | 1.06E-67 | 3.58E-66 |
| HMCN2 | 0.106533 | 0.307674 | 1.530109 | 4.37E-54 | 8.83E-53 |
| TSPAN7 | 2.235734 | 4.568146 | 1.03086 | 5.03E-38 | 5.38E-37 |
| MCTP1 | 0.652453 | 1.32457 | 1.021578 | 2.90E-69 | 1.05E-67 |
| TLR8 | 0.504217 | 1.085388 | 1.106093 | 3.17E-51 | 5.71E-50 |
| PDGFRB | 18.24541 | 40.35198 | 1.145106 | 2.51E-113 | 7.43E-111 |
| MRC2 | 15.27175 | 30.92317 | 1.017823 | 2.23E-77 | 1.11E-75 |
| TEX14 | 1.386705 | 0.507916 | -1.449 | 0.0052848 | 0.0074568 |
| B3GALT2 | 0.107751 | 0.259394 | 1.267445 | 1.97E-42 | 2.48E-41 |
| FCN1 | 0.478363 | 1.033146 | 1.110866 | 1.29E-26 | 9.03E-26 |
| MUC7 | 0.175108 | 0.629635 | 1.846272 | 2.62E-07 | 5.65E-07 |
| CALHM5 | 0.637519 | 1.635981 | 1.359615 | 9.24E-133 | 1.57E-129 |
| INHBA | 6.833857 | 15.70014 | 1.200005 | 2.11E-70 | 7.81E-69 |
| AL845331.2 | 0.051105 | 0.108602 | 1.087518 | 1.90E-12 | 6.07E-12 |
| C9orf84 | 0.080224 | 0.185678 | 1.210692 | 6.67E-09 | 1.64E-08 |
| FCRLB | 4.79609 | 1.585098 | -1.59729 | 5.77E-08 | 1.31E-07 |
| PODN | 5.76543 | 18.20932 | 1.659177 | 3.33E-116 | 1.19E-113 |
| CCL23 | 0.192137 | 0.418944 | 1.124621 | 2.38E-32 | 2.07E-31 |
| POSTN | 123.2275 | 337.551 | 1.453782 | 1.04E-92 | 9.86E-91 |
| GABRB2 | 0.102948 | 0.222709 | 1.113245 | 1.63E-36 | 1.67E-35 |
| FLRT2 | 0.254482 | 0.63067 | 1.309324 | 1.11E-98 | 1.42E-96 |
| CCDC36 | 0.093869 | 0.243231 | 1.373607 | 5.40E-97 | 6.24E-95 |
| LRFN5 | 0.109214 | 0.23104 | 1.080988 | 1.22E-58 | 2.90E-57 |
| MEDAG | 1.942231 | 5.409899 | 1.477887 | 8.93E-83 | 5.43E-81 |
| PRKCB | 0.587888 | 1.41444 | 1.266618 | 1.65E-43 | 2.18E-42 |
| CILP | 12.35948 | 47.91763 | 1.954939 | 2.37E-84 | 1.55E-82 |
| IL16 | 1.108987 | 2.471415 | 1.156094 | 1.72E-94 | 1.71E-92 |
| FREM1 | 0.201595 | 0.546229 | 1.438043 | 2.57E-53 | 5.08E-52 |
| TMEM119 | 7.818524 | 22.1104 | 1.499757 | 2.74E-111 | 6.66E-109 |
| CTSE | 0.160398 | 0.45829 | 1.514604 | 5.95E-08 | 1.35E-07 |
| SERPING1 | 52.10958 | 108.6095 | 1.05953 | 2.75E-102 | 4.26E-100 |
| ITGBL1 | 2.240355 | 6.590722 | 1.556709 | 1.36E-99 | 1.80E-97 |
| BARX2 | 10.30482 | 5.152001 | -1.00011 | 2.66E-09 | 6.78E-09 |
| WIPF1 | 4.853251 | 10.37168 | 1.095626 | 7.76E-112 | 2.07E-109 |
| OGDHL | 0.402277 | 0.117249 | -1.77861 | 7.37E-05 | 0.0001279 |
| C2orf70 | 0.649082 | 0.30915 | -1.07009 | 3.84E-08 | 8.84E-08 |
| CACNA1G | 0.100661 | 0.21437 | 1.090593 | 1.80E-44 | 2.46E-43 |
| EMILIN1 | 13.23986 | 31.98727 | 1.27261 | 2.69E-75 | 1.24E-73 |
| CAV1 | 12.70655 | 26.7061 | 1.071596 | 6.07E-79 | 3.15E-77 |
| MATN4 | 0.471536 | 0.171799 | -1.45665 | 0.0001269 | 0.0002158 |
| SIGLEC6 | 0.160861 | 0.398573 | 1.309027 | 4.81E-52 | 8.99E-51 |
| AVPR2 | 0.306333 | 0.647148 | 1.078992 | 2.81E-25 | 1.86E-24 |
| GAS7 | 2.600656 | 6.961094 | 1.420439 | 3.74E-123 | 2.68E-120 |
| FABP4 | 17.80234 | 66.22144 | 1.895232 | 5.23E-25 | 3.40E-24 |
| KCNH6 | 0.244542 | 0.052032 | -2.23262 | 1.52E-05 | 2.81E-05 |
| GPR158 | 0.525422 | 0.236613 | -1.15095 | 0.0167186 | 0.0221986 |
| CAMK2B | 1.454977 | 0.704901 | -1.0455 | 0.0215485 | 0.0282102 |
| PRTN3 | 0.421475 | 0.164415 | -1.3581 | 0.0004509 | 0.0007191 |
| SORCS2 | 1.069737 | 3.006845 | 1.490995 | 6.92E-80 | 3.71E-78 |
| CFH | 3.58856 | 9.338036 | 1.379714 | 5.16E-132 | 7.81E-129 |
| PLN | 1.393612 | 3.117987 | 1.161786 | 2.51E-63 | 7.22E-62 |
| VWF | 11.81884 | 23.67543 | 1.002302 | 3.20E-63 | 9.15E-62 |
| ROS1 | 0.073485 | 0.280982 | 1.934952 | 2.05E-10 | 5.69E-10 |
| CYP19A1 | 0.051837 | 0.922109 | 4.152892 | 2.45E-22 | 1.41E-21 |
| SVEP1 | 0.963781 | 2.783151 | 1.529941 | 9.63E-89 | 7.29E-87 |
| RFTN2 | 0.489847 | 0.980898 | 1.001771 | 2.79E-104 | 4.82E-102 |
| RASGRF2 | 0.717641 | 1.954387 | 1.445381 | 4.59E-127 | 4.17E-124 |
| FOXI3 | 0.420495 | 0.181747 | -1.21016 | 0.0097122 | 0.013288 |
| RUNX1T1 | 0.299792 | 0.729232 | 1.282413 | 9.97E-113 | 2.83E-110 |
| GPR1 | 0.325923 | 1.128387 | 1.791658 | 1.18E-81 | 6.82E-80 |
| TPSD1 | 1.147096 | 3.299646 | 1.524326 | 7.48E-15 | 2.78E-14 |
| CLEC10A | 1.102648 | 2.724016 | 1.304763 | 6.73E-31 | 5.61E-30 |
| PI16 | 1.878196 | 6.314912 | 1.749415 | 1.66E-22 | 9.58E-22 |
| DLGAP1 | 0.933146 | 0.393648 | -1.2452 | 0.0194074 | 0.0255793 |
| CPA1 | 0.022289 | 0.071084 | 1.673216 | 1.77E-17 | 7.81E-17 |
| PREX2 | 0.513023 | 1.115426 | 1.120499 | 8.83E-65 | 2.64E-63 |
| BMX | 0.279727 | 0.656883 | 1.231617 | 1.36E-41 | 1.65E-40 |
| PLIN1 | 3.644938 | 13.03997 | 1.838974 | 8.71E-22 | 4.88E-21 |
| MT1G | 20.29658 | 9.548202 | -1.08794 | 0.0006182 | 0.0009704 |
| THBS2 | 32.44903 | 91.6497 | 1.497955 | 2.50E-101 | 3.70E-99 |
| COL8A1 | 6.737376 | 18.21657 | 1.434993 | 2.08E-91 | 1.84E-89 |
| ADH1B | 2.547078 | 10.29917 | 2.015613 | 4.81E-30 | 3.89E-29 |
| BTLA | 0.269697 | 0.623978 | 1.210154 | 4.91E-29 | 3.82E-28 |
| C1orf105 | 0.40275 | 0.064962 | -2.63222 | 0.0013826 | 0.0020877 |
| MPEG1 | 5.623424 | 12.11421 | 1.10718 | 5.05E-64 | 1.48E-62 |
| DCN | 22.11266 | 76.57603 | 1.792021 | 2.29E-145 | 1.56E-141 |
| DACT1 | 2.204231 | 5.745252 | 1.382095 | 2.51E-112 | 6.98E-110 |
| CD69 | 1.406053 | 3.292349 | 1.227466 | 1.10E-43 | 1.47E-42 |
| PRDM1 | 2.224031 | 4.573666 | 1.040174 | 8.66E-92 | 7.82E-90 |
| C8orf86 | 0.298652 | 0.09741 | -1.61633 | 4.03E-07 | 8.54E-07 |
| CD248 | 12.34331 | 26.19586 | 1.08561 | 7.08E-78 | 3.58E-76 |
| DIRC1 | 0.112334 | 0.266647 | 1.247135 | 3.84E-55 | 8.05E-54 |
| PDK4 | 7.45655 | 15.74824 | 1.078611 | 1.42E-24 | 9.02E-24 |
| ADH1A | 0.024849 | 0.062433 | 1.329094 | 8.44E-20 | 4.24E-19 |
| ACTL8 | 4.366703 | 1.766039 | -1.30603 | 2.95E-07 | 6.32E-07 |
| CST9L | 0.586685 | 0.277307 | -1.0811 | 0.0108506 | 0.0147553 |
| CYR61 | 49.01804 | 114.254 | 1.22086 | 4.28E-54 | 8.69E-53 |
| HIC1 | 0.867615 | 1.857965 | 1.098596 | 4.73E-78 | 2.40E-76 |
| DMBT1 | 0.16558 | 1.255516 | 2.922676 | 5.87E-15 | 2.20E-14 |
| CYTL1 | 0.731245 | 1.569307 | 1.1017 | 2.31E-42 | 2.90E-41 |
| NMU | 3.4589 | 1.03087 | -1.74645 | 1.15E-06 | 2.33E-06 |
| GNG2 | 1.784301 | 3.886122 | 1.122972 | 1.25E-109 | 2.66E-107 |
| SGCD | 1.07105 | 2.867034 | 1.420533 | 2.30E-96 | 2.59E-94 |
| RUNX2 | 1.835719 | 3.869471 | 1.075791 | 1.80E-89 | 1.45E-87 |
| PLEK | 6.173066 | 12.35478 | 1.00101 | 1.99E-51 | 3.59E-50 |
| EPYC | 1.03185 | 3.712687 | 1.847231 | 6.14E-32 | 5.27E-31 |
| GEM | 7.297982 | 16.97867 | 1.218154 | 7.32E-60 | 1.80E-58 |
| GYPE | 0.093331 | 0.190436 | 1.028878 | 6.48E-59 | 1.55E-57 |
| LYZ | 64.73854 | 130.5277 | 1.01166 | 2.42E-35 | 2.36E-34 |
| ITGA11 | 4.248664 | 11.66294 | 1.456851 | 3.49E-68 | 1.21E-66 |
| FCRL3 | 0.297588 | 0.605656 | 1.025181 | 8.94E-24 | 5.51E-23 |
| GLIS1 | 0.233928 | 0.470697 | 1.008736 | 8.06E-52 | 1.48E-50 |
| SPIN2A | 0.058623 | 0.147993 | 1.335991 | 1.27E-36 | 1.30E-35 |
| ICAM3 | 0.450191 | 0.92934 | 1.045667 | 5.88E-41 | 6.95E-40 |
| KCNH2 | 1.504201 | 0.553882 | -1.44135 | 9.22E-08 | 2.06E-07 |
| FAM189A1 | 0.212724 | 0.432334 | 1.023162 | 2.25E-23 | 1.36E-22 |
| TFEC | 0.723651 | 1.507141 | 1.058448 | 9.23E-49 | 1.50E-47 |
| CPA3 | 6.468937 | 14.57665 | 1.172058 | 2.32E-47 | 3.55E-46 |
| AICDA | 0.03036 | 0.23169 | 2.931929 | 3.91E-19 | 1.90E-18 |
| PGC | 0.726465 | 1.868463 | 1.362887 | 0.0193454 | 0.0255024 |
| TFF2 | 0.720117 | 0.307164 | -1.22922 | 0.0110714 | 0.0150434 |
| ARHGAP20 | 0.211769 | 0.549296 | 1.37509 | 3.91E-96 | 4.30E-94 |
| C1QL4 | 0.583605 | 0.237076 | -1.29964 | 0.0027629 | 0.0040381 |
| THSD7B | 0.17031 | 0.378455 | 1.151955 | 4.08E-42 | 5.07E-41 |
| MS4A4E | 0.153523 | 0.31816 | 1.051292 | 1.71E-34 | 1.62E-33 |
| SCML4 | 0.145899 | 0.332599 | 1.188818 | 6.93E-33 | 6.16E-32 |
| RPL3L | 0.493514 | 0.106358 | -2.21416 | 6.92E-12 | 2.13E-11 |
| FYB1 | 3.255096 | 6.808681 | 1.064675 | 2.19E-54 | 4.51E-53 |
| IL33 | 2.330364 | 5.631996 | 1.273091 | 1.65E-35 | 1.62E-34 |
| SOHLH1 | 0.174368 | 0.07266 | -1.2629 | 0.0054112 | 0.0076272 |
| CTGF | 93.86885 | 226.3519 | 1.269849 | 9.17E-79 | 4.72E-77 |
| LRRC32 | 7.05801 | 15.98232 | 1.179144 | 3.52E-106 | 6.39E-104 |
| CLEC2B | 2.946792 | 6.03807 | 1.034942 | 9.84E-107 | 1.86E-104 |
| TLR7 | 1.221498 | 2.799071 | 1.196296 | 5.08E-68 | 1.74E-66 |
| SLC1A7 | 0.11824 | 0.314713 | 1.412322 | 7.29E-79 | 3.76E-77 |
| ANGPT4 | 0.052765 | 0.12888 | 1.28839 | 1.33E-42 | 1.69E-41 |
| CIDEA | 0.755142 | 2.303232 | 1.608841 | 1.73E-18 | 8.11E-18 |
| TNN | 1.138107 | 3.610279 | 1.665474 | 2.61E-60 | 6.54E-59 |
| RECK | 1.55901 | 3.856194 | 1.306547 | 8.79E-124 | 6.65E-121 |
| HEPACAM | 0.03171 | 0.106876 | 1.752911 | 8.77E-19 | 4.18E-18 |
| PCDH7 | 1.253929 | 2.84663 | 1.1828 | 1.03E-80 | 5.70E-79 |
| KCNA1 | 0.04398 | 0.111328 | 1.339897 | 1.67E-49 | 2.83E-48 |
| VCAM1 | 4.566524 | 9.998392 | 1.1306 | 4.08E-58 | 9.45E-57 |
| MS4A1 | 1.496683 | 3.226325 | 1.108123 | 1.24E-20 | 6.46E-20 |
| TMEM82 | 0.176633 | 0.059718 | -1.56453 | 0.0055271 | 0.0077833 |
| ADAMTS6 | 0.307634 | 0.671192 | 1.125509 | 8.07E-72 | 3.22E-70 |
| CCL14 | 0.306247 | 0.842655 | 1.460247 | 2.16E-38 | 2.34E-37 |
| FBN3 | 0.495689 | 0.240669 | -1.04238 | 0.0009886 | 0.0015184 |
| DDR2 | 3.215207 | 6.558668 | 1.028491 | 2.23E-96 | 2.54E-94 |
| CYP2C8 | 0.568878 | 0.282515 | -1.00979 | 0.0218528 | 0.0285838 |
| ANGPTL2 | 10.34847 | 27.16876 | 1.392531 | 4.59E-118 | 1.79E-115 |
| STAB2 | 0.057557 | 0.121989 | 1.083696 | 1.02E-16 | 4.29E-16 |
| ARHGAP28 | 0.422714 | 0.97551 | 1.206473 | 3.90E-96 | 4.30E-94 |
| NR5A1 | 0.178726 | 0.083648 | -1.09535 | 0.0250656 | 0.0325428 |
| ABCA9 | 0.278138 | 0.912697 | 1.714336 | 2.64E-71 | 1.02E-69 |
| CPXM1 | 8.048112 | 23.11782 | 1.522283 | 5.37E-80 | 2.92E-78 |
| OMD | 2.129481 | 8.835265 | 2.052771 | 2.79E-103 | 4.52E-101 |
| POU5F1B | 0.365301 | 0.155192 | -1.23504 | 0.0007849 | 0.0012186 |
| F2RL2 | 5.68727 | 13.90407 | 1.289699 | 2.81E-52 | 5.33E-51 |
| KLRB1 | 1.11711 | 2.549651 | 1.190529 | 1.52E-42 | 1.93E-41 |
| LAX1 | 0.644498 | 1.488307 | 1.207424 | 1.84E-40 | 2.13E-39 |
| TUBB4A | 0.823787 | 0.357801 | -1.20311 | 0.0025921 | 0.0038011 |
| PLPP7 | 0.566463 | 1.227516 | 1.115688 | 1.69E-94 | 1.69E-92 |
| NEURL1 | 4.334451 | 2.111386 | -1.03766 | 4.59E-05 | 8.11E-05 |
| PRSS35 | 0.155313 | 0.425457 | 1.453835 | 2.41E-34 | 2.27E-33 |
| KLK4 | 0.90769 | 2.739817 | 1.593808 | 2.78E-74 | 1.22E-72 |
| PDGFRA | 3.24265 | 7.364472 | 1.183409 | 9.88E-111 | 2.36E-108 |
| TSPAN11 | 0.539382 | 1.097093 | 1.024305 | 7.06E-65 | 2.13E-63 |
| FCRL5 | 0.265989 | 0.557356 | 1.067236 | 2.15E-22 | 1.24E-21 |
| DLGAP3 | 0.395019 | 0.181122 | -1.12496 | 0.0001199 | 0.000204 |
| FCRL1 | 0.173139 | 0.45974 | 1.408887 | 2.13E-18 | 9.92E-18 |
| GFRA3 | 1.515644 | 0.260384 | -2.54122 | 0.0109255 | 0.0148526 |
| GRID1 | 0.353023 | 0.713182 | 1.01451 | 1.49E-83 | 9.33E-82 |
| MSRB3 | 4.24139 | 10.05291 | 1.245004 | 3.47E-106 | 6.39E-104 |
| LY9 | 0.229914 | 0.520732 | 1.179446 | 1.09E-38 | 1.20E-37 |
| MXRA5 | 21.79114 | 59.12457 | 1.440016 | 5.72E-113 | 1.66E-110 |
| NRTN | 1.971072 | 0.824593 | -1.25723 | 1.69E-10 | 4.71E-10 |
| METTL11B | 0.106613 | 0.268993 | 1.335188 | 3.38E-31 | 2.85E-30 |
| CPED1 | 1.025991 | 2.195665 | 1.09764 | 4.97E-85 | 3.35E-83 |
| SLFN12L | 0.220362 | 0.50106 | 1.185111 | 6.80E-39 | 7.50E-38 |
| EEF1A2 | 75.83674 | 35.869 | -1.08016 | 0.0005717 | 0.0009014 |
| FMO1 | 1.385907 | 3.177383 | 1.197009 | 4.03E-56 | 8.69E-55 |
| MMP11 | 40.65227 | 84.76111 | 1.060067 | 1.00E-34 | 9.59E-34 |
| LAMA4 | 6.08155 | 12.50882 | 1.040435 | 2.23E-113 | 6.75E-111 |
| NLRP3 | 0.576507 | 1.227823 | 1.090693 | 1.05E-72 | 4.39E-71 |
| ELAVL3 | 0.231661 | 0.021089 | -3.45748 | 5.44E-09 | 1.35E-08 |
| CLDN18 | 0.052878 | 0.147582 | 1.480774 | 4.19E-18 | 1.92E-17 |
| ACTL6B | 0.411427 | 0.027228 | -3.91748 | 3.90E-06 | 7.58E-06 |
| DDN | 0.256862 | 0.082529 | -1.63801 | 5.44E-08 | 1.24E-07 |
| CHRDL1 | 2.21126 | 6.759733 | 1.612098 | 1.58E-33 | 1.44E-32 |
| NT5E | 2.975544 | 5.952532 | 1.00035 | 3.40E-84 | 2.20E-82 |
| MYL3 | 0.196498 | 0.441865 | 1.169088 | 2.84E-91 | 2.46E-89 |
| TNFRSF17 | 1.086446 | 2.208212 | 1.023262 | 3.85E-25 | 2.53E-24 |
| IL12B | 0.085841 | 0.177059 | 1.044485 | 4.51E-17 | 1.94E-16 |
| PGM5 | 0.630564 | 1.499831 | 1.250085 | 3.96E-54 | 8.06E-53 |
| RARRES2 | 14.83842 | 32.14059 | 1.11506 | 5.70E-69 | 2.04E-67 |
| GPR15 | 0.151695 | 0.405732 | 1.419355 | 8.50E-24 | 5.25E-23 |
| TMSB15A | 6.599987 | 2.432252 | -1.44017 | 9.59E-08 | 2.14E-07 |
| LAMP5 | 5.19105 | 10.89069 | 1.068997 | 7.04E-57 | 1.56E-55 |
| CLDN11 | 0.960391 | 1.947683 | 1.020065 | 3.60E-33 | 3.23E-32 |
| CD5 | 1.758685 | 3.717811 | 1.079956 | 1.01E-35 | 9.94E-35 |
| EOMES | 0.48621 | 1.112158 | 1.19371 | 1.12E-32 | 9.85E-32 |
| SLIT3 | 2.188058 | 5.310164 | 1.279106 | 6.02E-103 | 9.54E-101 |
| FAP | 3.555724 | 10.09259 | 1.505081 | 1.05E-119 | 4.60E-117 |
| TBX18 | 0.48752 | 1.142808 | 1.229051 | 2.77E-101 | 4.06E-99 |
| SLC1A2 | 2.562235 | 1.128096 | -1.18351 | 8.70E-06 | 1.64E-05 |
| TM6SF1 | 0.846825 | 1.778914 | 1.07086 | 7.85E-91 | 6.72E-89 |
| AKR1B15 | 1.264069 | 4.500751 | 1.83209 | 4.31E-08 | 9.87E-08 |
| TWIST2 | 1.402212 | 3.047444 | 1.119895 | 1.33E-68 | 4.68E-67 |
| COL8A2 | 8.431631 | 22.73115 | 1.430787 | 2.60E-89 | 2.06E-87 |
| LVRN | 0.060903 | 0.14115 | 1.212645 | 6.76E-23 | 4.00E-22 |
| CSDC2 | 0.44735 | 1.130419 | 1.337383 | 3.40E-58 | 7.93E-57 |
| FSTL1 | 24.92146 | 62.0924 | 1.317028 | 3.45E-136 | 9.40E-133 |
| GALNT15 | 0.882316 | 3.093975 | 1.810095 | 4.96E-103 | 7.95E-101 |
| FBLN2 | 20.8828 | 45.19877 | 1.113968 | 6.87E-98 | 8.35E-96 |
| TMEM255A | 0.481712 | 0.979264 | 1.023527 | 9.85E-58 | 2.25E-56 |
| SLC27A2 | 11.53841 | 4.977918 | -1.21283 | 0.0014713 | 0.0022138 |
| SCN2B | 0.098185 | 0.266289 | 1.439418 | 5.98E-62 | 1.61E-60 |
| NPR1 | 1.355296 | 2.745228 | 1.018318 | 5.84E-38 | 6.24E-37 |
| INMT | 0.764683 | 1.74811 | 1.192862 | 2.24E-42 | 2.81E-41 |
| SELP | 1.3732 | 3.783028 | 1.461999 | 3.34E-40 | 3.84E-39 |
| WNT2 | 2.087427 | 5.225409 | 1.323818 | 1.84E-79 | 9.67E-78 |
| TPSB2 | 7.730699 | 16.9745 | 1.134698 | 6.72E-29 | 5.19E-28 |
| MSI1 | 2.907775 | 1.27801 | -1.18602 | 5.89E-09 | 1.46E-08 |
| PRND | 0.574486 | 1.447016 | 1.332739 | 2.13E-38 | 2.31E-37 |
| TMEM252 | 0.043633 | 0.108537 | 1.314687 | 1.07E-35 | 1.06E-34 |
| TPSAB1 | 5.864052 | 13.63354 | 1.217191 | 9.58E-35 | 9.17E-34 |
| WISP2 | 2.846939 | 9.056388 | 1.669524 | 3.86E-61 | 1.00E-59 |
| APOA1 | 1.275734 | 0.284985 | -2.16237 | 0.0205907 | 0.0270421 |
| ANTXR1 | 18.13204 | 44.38144 | 1.291415 | 1.48E-86 | 1.08E-84 |
| HSD17B13 | 0.072971 | 0.174619 | 1.258808 | 2.01E-33 | 1.82E-32 |
| AGXT | 0.081389 | 0.250111 | 1.619663 | 5.54E-09 | 1.38E-08 |
| SERPINF1 | 32.46762 | 86.00768 | 1.405464 | 1.24E-127 | 1.21E-124 |
| GLT8D2 | 3.326296 | 9.166827 | 1.462506 | 7.25E-123 | 4.94E-120 |
| GIMAP6 | 3.704693 | 7.531634 | 1.023609 | 1.61E-71 | 6.32E-70 |
| TFR2 | 1.063502 | 0.526536 | -1.01422 | 6.51E-12 | 2.01E-11 |
| TAGLN3 | 0.321384 | 0.071428 | -2.16975 | 0.024128 | 0.031397 |
| SELE | 1.500292 | 3.025322 | 1.011845 | 5.13E-28 | 3.80E-27 |
| PLIN4 | 4.575473 | 14.92737 | 1.705967 | 6.12E-15 | 2.29E-14 |
| MRO | 0.233786 | 0.748035 | 1.67792 | 3.46E-34 | 3.23E-33 |
| CAVIN2 | 1.754712 | 4.541024 | 1.371783 | 3.78E-43 | 4.91E-42 |
| CNR2 | 0.084207 | 0.195477 | 1.214986 | 6.36E-20 | 3.22E-19 |
| SLAMF1 | 0.547629 | 1.190767 | 1.120621 | 6.28E-38 | 6.70E-37 |
| CD40LG | 0.552157 | 1.233605 | 1.159729 | 3.02E-36 | 3.06E-35 |
| GSTM5 | 0.676093 | 1.722352 | 1.349086 | 3.79E-50 | 6.57E-49 |
| ACSM5 | 0.28755 | 0.61503 | 1.096847 | 1.61E-51 | 2.93E-50 |
| LILRB5 | 0.311868 | 0.678233 | 1.120843 | 6.84E-40 | 7.77E-39 |
| LEPR | 0.727854 | 1.461757 | 1.005983 | 1.82E-39 | 2.04E-38 |
| FLNC | 0.978347 | 2.13357 | 1.124851 | 1.09E-83 | 6.88E-82 |
| AOAH | 1.830354 | 3.708033 | 1.018531 | 7.72E-52 | 1.42E-50 |
| PLPP4 | 2.685654 | 6.948371 | 1.371401 | 9.94E-69 | 3.51E-67 |
| FCRL6 | 0.331802 | 0.672672 | 1.019583 | 3.69E-30 | 3.00E-29 |
| CTHRC1 | 36.45565 | 76.84063 | 1.075727 | 3.20E-85 | 2.18E-83 |
| CLDN5 | 1.996105 | 4.477858 | 1.165622 | 1.93E-28 | 1.46E-27 |
| KCNA5 | 0.147003 | 0.315254 | 1.100672 | 8.92E-29 | 6.86E-28 |
| CRISPLD2 | 5.9527 | 17.33643 | 1.542191 | 1.18E-131 | 1.61E-128 |
| CD36 | 5.625632 | 12.18012 | 1.114441 | 3.15E-24 | 1.97E-23 |
| PTGDS | 6.346204 | 15.82694 | 1.318416 | 4.24E-34 | 3.96E-33 |
| ACVR1C | 0.153279 | 0.358331 | 1.225134 | 6.63E-21 | 3.52E-20 |
| CORO2B | 0.573688 | 1.280687 | 1.158579 | 1.74E-74 | 7.76E-73 |
| CD209 | 0.568904 | 1.264251 | 1.152026 | 6.27E-37 | 6.51E-36 |
| ANGPTL1 | 0.473886 | 1.665881 | 1.813675 | 1.80E-82 | 1.08E-80 |
| MMP13 | 5.614016 | 17.60007 | 1.648476 | 6.76E-36 | 6.71E-35 |
| SPOCK1 | 4.688887 | 11.49382 | 1.293541 | 1.44E-77 | 7.23E-76 |
| CD200R1 | 0.289558 | 0.618466 | 1.094843 | 2.56E-49 | 4.28E-48 |
| SLC38A4 | 0.166779 | 0.431802 | 1.372436 | 1.16E-80 | 6.38E-79 |
| KLK3 | 0.172175 | 0.57254 | 1.733496 | 1.16E-20 | 6.06E-20 |
| COL16A1 | 8.260506 | 17.04608 | 1.045138 | 6.29E-65 | 1.91E-63 |
| KLK2 | 0.044105 | 0.229685 | 2.380645 | 7.66E-57 | 1.68E-55 |
| WISP1 | 3.031717 | 8.36899 | 1.464918 | 5.52E-119 | 2.35E-116 |
| FGF18 | 0.511353 | 1.068605 | 1.063337 | 4.82E-57 | 1.07E-55 |
| LGALS12 | 0.73863 | 1.717864 | 1.217693 | 5.28E-17 | 2.26E-16 |
| GPD1 | 2.242113 | 7.798492 | 1.798336 | 9.06E-18 | 4.07E-17 |
| CSF2RB | 2.289215 | 5.051519 | 1.141864 | 3.93E-60 | 9.83E-59 |
| C7orf61 | 0.389896 | 0.182384 | -1.09611 | 0.0002031 | 0.0003375 |
| C7 | 2.64362 | 7.654634 | 1.533819 | 4.25E-35 | 4.11E-34 |
| NCAN | 1.452753 | 0.364375 | -1.99529 | 0.023832 | 0.0310238 |
| AADAC | 0.216678 | 0.595169 | 1.457744 | 8.27E-15 | 3.06E-14 |
| C1QTNF3 | 3.156007 | 8.280852 | 1.391679 | 4.26E-52 | 7.99E-51 |
| CHSY3 | 0.854604 | 2.014811 | 1.237316 | 1.42E-108 | 2.85E-106 |
| CCL19 | 18.40938 | 39.53494 | 1.102687 | 9.19E-23 | 5.39E-22 |
| TFCP2L1 | 6.566775 | 3.266349 | -1.00751 | 5.77E-10 | 1.55E-09 |
| MRVI1 | 1.626172 | 3.497691 | 1.104924 | 4.28E-96 | 4.63E-94 |
| AP3B2 | 0.96651 | 0.416413 | -1.21477 | 4.16E-07 | 8.81E-07 |
| PAH | 0.461378 | 0.189795 | -1.28151 | 0.0008851 | 0.0013673 |
| NTSR2 | 0.270395 | 0.023479 | -3.52561 | 6.00E-16 | 2.40E-15 |
| ALDH1A1 | 5.655156 | 11.78433 | 1.059231 | 6.97E-80 | 3.72E-78 |
| ADAMTS16 | 0.999954 | 2.827224 | 1.499452 | 1.06E-64 | 3.15E-63 |
| SLURP1 | 1.751451 | 0.695405 | -1.33263 | 0.0034794 | 0.0050196 |
| KCNIP2 | 0.756463 | 1.828815 | 1.273568 | 3.88E-11 | 1.13E-10 |
| APLP1 | 6.602709 | 2.131287 | -1.63133 | 5.23E-11 | 1.51E-10 |
| SYNDIG1 | 1.360325 | 4.345415 | 1.675542 | 2.08E-80 | 1.13E-78 |
| TMPRSS6 | 3.145692 | 1.520081 | -1.04923 | 0.009447 | 0.0129434 |
| SLC24A2 | 0.254162 | 0.922674 | 1.86007 | 9.14E-77 | 4.46E-75 |
| ADH1C | 0.571614 | 1.214942 | 1.087775 | 4.60E-26 | 3.14E-25 |
| LRP1 | 11.28476 | 28.56577 | 1.339912 | 7.71E-122 | 4.56E-119 |
| DHH | 0.155382 | 0.311148 | 1.001784 | 1.08E-35 | 1.07E-34 |
| SULT1B1 | 0.09949 | 0.325996 | 1.712233 | 1.42E-30 | 1.17E-29 |
| ABCC9 | 0.715657 | 1.434214 | 1.002919 | 1.01E-69 | 3.70E-68 |
| TCF4 | 2.23096 | 4.904405 | 1.136414 | 1.44E-120 | 6.77E-118 |
| PARP15 | 0.366408 | 0.796651 | 1.120497 | 3.79E-42 | 4.73E-41 |
| ITIH5 | 0.890817 | 2.168036 | 1.283188 | 1.65E-42 | 2.08E-41 |
| GRAP2 | 0.43801 | 0.876734 | 1.001176 | 7.07E-41 | 8.34E-40 |
| COMP | 25.79598 | 66.60772 | 1.368543 | 4.07E-54 | 8.28E-53 |
| IL6 | 0.864339 | 2.068749 | 1.25909 | 2.94E-19 | 1.43E-18 |
| ZNF366 | 0.377358 | 0.830788 | 1.138544 | 3.83E-58 | 8.88E-57 |
| MXRA8 | 25.46899 | 52.01643 | 1.030226 | 2.88E-69 | 1.04E-67 |
| HTRA3 | 18.65565 | 48.52581 | 1.37914 | 1.91E-76 | 9.18E-75 |
| KLHL4 | 0.090081 | 0.250809 | 1.4773 | 1.20E-62 | 3.37E-61 |
| PM20D1 | 0.068154 | 0.165217 | 1.27749 | 2.83E-37 | 2.96E-36 |
| SLAMF6 | 1.457359 | 3.273881 | 1.167645 | 4.88E-37 | 5.08E-36 |
| CASQ2 | 0.441751 | 0.915467 | 1.051273 | 9.25E-32 | 7.91E-31 |
| CEL | 2.857485 | 1.146528 | -1.31747 | 6.49E-09 | 1.60E-08 |
| UBASH3A | 0.484348 | 0.974503 | 1.008622 | 3.45E-32 | 2.99E-31 |
| FN1 | 177.9491 | 415.7633 | 1.224298 | 5.87E-69 | 2.10E-67 |
| BRINP2 | 3.327171 | 0.834485 | -1.99534 | 1.82E-05 | 3.34E-05 |
| GFPT2 | 2.908864 | 5.94663 | 1.031616 | 7.00E-97 | 8.02E-95 |
| DPYSL3 | 14.46163 | 34.08741 | 1.237009 | 1.29E-115 | 4.52E-113 |
| IGF1 | 0.176015 | 0.571789 | 1.699786 | 3.12E-75 | 1.43E-73 |
| ANTXR2 | 2.576371 | 5.161515 | 1.002454 | 2.03E-111 | 5.03E-109 |
| ADAMTS14 | 0.907332 | 1.918724 | 1.080445 | 3.81E-54 | 7.77E-53 |
| ARL9 | 2.297786 | 1.034884 | -1.15078 | 4.31E-06 | 8.36E-06 |
| BLK | 0.247945 | 0.521546 | 1.072774 | 1.25E-17 | 5.57E-17 |
| ASCL1 | 3.856598 | 1.432428 | -1.42887 | 0.0060844 | 0.0085293 |
| SLC19A3 | 0.474628 | 1.381922 | 1.541808 | 6.32E-17 | 2.68E-16 |
| GZMM | 1.305251 | 2.643786 | 1.018278 | 8.80E-22 | 4.93E-21 |
| ZCCHC24 | 5.194397 | 11.18034 | 1.105936 | 2.45E-91 | 2.14E-89 |
| OGN | 5.021666 | 10.88331 | 1.115879 | 3.95E-74 | 1.74E-72 |
| ST6GAL2 | 0.902337 | 2.452378 | 1.442443 | 1.20E-71 | 4.73E-70 |
| KIF26B | 1.788657 | 3.580847 | 1.001424 | 2.51E-65 | 7.71E-64 |
| MYOM2 | 0.316704 | 0.929084 | 1.552673 | 2.09E-09 | 5.38E-09 |
| MT3 | 0.202432 | 0.082214 | -1.29998 | 1.15E-05 | 2.14E-05 |
| KRT10 | 26.42095 | 12.91608 | -1.03251 | 0.0010974 | 0.0016767 |
| VXN | 1.209072 | 0.481962 | -1.32691 | 0.0197261 | 0.0259867 |
| PLAC9 | 2.329966 | 5.827166 | 1.322486 | 7.93E-50 | 1.36E-48 |
| CASS4 | 0.31404 | 0.646127 | 1.040871 | 4.44E-59 | 1.07E-57 |
| MAST1 | 0.489763 | 0.182133 | -1.42709 | 9.98E-16 | 3.94E-15 |
| SEC14L4 | 0.208772 | 0.100067 | -1.06096 | 0.0095483 | 0.0130743 |
| GLYAT | 0.061996 | 0.233193 | 1.911273 | 1.30E-14 | 4.75E-14 |
| VGF | 0.984084 | 0.348008 | -1.49966 | 1.21E-15 | 4.76E-15 |
| LRRC17 | 3.366161 | 9.319382 | 1.46913 | 3.35E-66 | 1.06E-64 |
| PYHIN1 | 0.451427 | 0.967734 | 1.100117 | 5.54E-35 | 5.34E-34 |
| ABCB1 | 0.537905 | 1.168822 | 1.119633 | 1.80E-45 | 2.56E-44 |
| COL5A1 | 25.85375 | 69.27044 | 1.421866 | 1.08E-99 | 1.46E-97 |
| FMR1NB | 0.439245 | 0.044099 | -3.31622 | 0.0232655 | 0.0303153 |
| FHL5 | 0.512104 | 1.15799 | 1.177113 | 1.66E-47 | 2.57E-46 |
| CLEC4G | 0.066777 | 0.147081 | 1.13919 | 3.05E-15 | 1.16E-14 |
| PECAM1 | 14.73484 | 29.79892 | 1.016029 | 5.63E-95 | 5.81E-93 |
| AEBP1 | 88.77119 | 222.8131 | 1.32767 | 2.24E-97 | 2.65E-95 |
| SLC38A3 | 1.948472 | 0.958515 | -1.02347 | 0.0034374 | 0.0049621 |
| CEMIP | 1.85064 | 4.306026 | 1.218333 | 4.49E-31 | 3.77E-30 |
| PSORS1C2 | 1.328851 | 0.614856 | -1.11186 | 0.0078196 | 0.0108052 |
| DPT | 9.449278 | 34.06637 | 1.850072 | 6.86E-104 | 1.15E-101 |
| GPX3 | 15.20066 | 31.49933 | 1.051187 | 1.28E-31 | 1.09E-30 |
| CELF3 | 0.360098 | 0.090357 | -1.99469 | 0.0001617 | 0.0002715 |
| TERT | 0.167256 | 0.068687 | -1.28396 | 1.83E-15 | 7.09E-15 |
| ARSI | 1.027849 | 2.196641 | 1.09567 | 1.37E-64 | 4.07E-63 |
| GIMAP7 | 6.698803 | 13.4631 | 1.007036 | 2.09E-45 | 2.97E-44 |
| TLL1 | 0.389152 | 0.990758 | 1.348197 | 5.11E-94 | 5.00E-92 |
| PLAU | 21.8906 | 44.85677 | 1.035015 | 1.98E-52 | 3.79E-51 |
| C2CD4B | 0.696109 | 1.499513 | 1.107109 | 4.79E-20 | 2.44E-19 |
| LHFPL6 | 10.1993 | 22.03066 | 1.111043 | 2.80E-96 | 3.13E-94 |
| EVI2A | 3.080315 | 6.948784 | 1.173683 | 4.16E-96 | 4.54E-94 |
| MMP19 | 1.692224 | 4.274711 | 1.336907 | 8.60E-122 | 4.88E-119 |
| SPOCK3 | 0.042688 | 0.138242 | 1.695289 | 1.37E-12 | 4.42E-12 |
| BACH2 | 0.407249 | 0.82748 | 1.022813 | 3.05E-68 | 1.06E-66 |
| CTSK | 57.65663 | 177.1722 | 1.619594 | 6.78E-135 | 1.54E-131 |
| SGIP1 | 0.480134 | 1.00712 | 1.068725 | 1.09E-66 | 3.52E-65 |
| RTBDN | 1.386442 | 0.380818 | -1.86421 | 1.52E-07 | 3.33E-07 |
| LOX | 6.36003 | 16.40994 | 1.367465 | 2.07E-103 | 3.39E-101 |
| CD300LG | 0.276544 | 0.931545 | 1.752116 | 1.72E-24 | 1.09E-23 |
| EGR1 | 52.15545 | 113.6547 | 1.123768 | 4.51E-32 | 3.89E-31 |
| ADGRA2 | 3.298279 | 7.258634 | 1.137985 | 3.28E-97 | 3.85E-95 |
| MYH11 | 2.946255 | 6.5436 | 1.151202 | 3.02E-40 | 3.48E-39 |
| MFAP5 | 6.910442 | 19.14051 | 1.46978 | 3.33E-85 | 2.26E-83 |
| CRTAM | 0.380184 | 0.870489 | 1.195126 | 1.68E-54 | 3.47E-53 |
| DOCK2 | 1.301009 | 2.772758 | 1.09169 | 3.80E-63 | 1.08E-61 |
| SLC18A2 | 0.232173 | 0.486262 | 1.066533 | 1.44E-41 | 1.74E-40 |
| PCOLCE | 16.9009 | 40.71914 | 1.268607 | 9.47E-90 | 7.68E-88 |
| LRCH2 | 0.291026 | 0.630461 | 1.115257 | 3.40E-105 | 6.01E-103 |
| CYBB | 9.878907 | 20.02537 | 1.019406 | 4.07E-52 | 7.64E-51 |
| CAMK2N2 | 1.088878 | 0.480982 | -1.17879 | 2.48E-08 | 5.82E-08 |
| GIMAP8 | 2.220617 | 4.461985 | 1.006725 | 3.31E-68 | 1.15E-66 |
| CXCL12 | 11.32373 | 29.67829 | 1.390059 | 1.37E-111 | 3.53E-109 |
| C1S | 36.93529 | 75.55286 | 1.032486 | 9.67E-112 | 2.53E-109 |
| SAMD3 | 0.138566 | 0.311042 | 1.166536 | 4.44E-42 | 5.52E-41 |
| BHMT2 | 0.78019 | 1.586576 | 1.024018 | 4.78E-81 | 2.69E-79 |
| P2RY12 | 0.481161 | 0.997613 | 1.05196 | 3.34E-45 | 4.70E-44 |
| CA9 | 5.69599 | 2.292517 | -1.31301 | 0.0002809 | 0.0004586 |
| COL6A6 | 0.168387 | 0.444027 | 1.39887 | 1.59E-53 | 3.18E-52 |
| MGARP | 0.173107 | 0.467076 | 1.431991 | 1.80E-27 | 1.30E-26 |
| PRSS3 | 0.443789 | 0.140554 | -1.65875 | 0.0136404 | 0.0183239 |
| CD3E | 4.610073 | 9.429792 | 1.032437 | 5.52E-34 | 5.13E-33 |
| CCR5 | 2.386187 | 4.855231 | 1.024833 | 2.35E-43 | 3.08E-42 |
| LRRC70 | 0.184424 | 0.378173 | 1.036023 | 1.17E-42 | 1.49E-41 |
| CH25H | 0.958631 | 2.317344 | 1.273424 | 4.33E-56 | 9.30E-55 |
| CR2 | 0.650974 | 1.648963 | 1.340887 | 2.79E-08 | 6.52E-08 |
| TCL1A | 0.620547 | 1.246434 | 1.006194 | 7.63E-12 | 2.34E-11 |
| F13A1 | 5.279839 | 18.95048 | 1.843668 | 1.04E-110 | 2.45E-108 |
| SBSN | 5.465731 | 0.87971 | -2.63531 | 0.0029042 | 0.0042318 |
| XCR1 | 0.158164 | 0.396575 | 1.32617 | 3.44E-33 | 3.09E-32 |
| P2RY13 | 1.131854 | 2.326691 | 1.039591 | 6.61E-51 | 1.18E-49 |
| COL5A2 | 39.73406 | 117.7567 | 1.567361 | 4.75E-114 | 1.51E-111 |
| LILRA4 | 0.50532 | 1.06534 | 1.076044 | 3.19E-35 | 3.11E-34 |
| GPR174 | 0.464753 | 1.206756 | 1.376598 | 3.45E-36 | 3.47E-35 |
| GRP | 3.872549 | 8.141842 | 1.072072 | 4.81E-41 | 5.73E-40 |
| TSHZ3 | 1.615457 | 3.624931 | 1.166011 | 3.56E-117 | 1.35E-114 |
| CPLX2 | 2.525712 | 0.198912 | -3.66649 | 0.0378299 | 0.0480474 |
| SULT4A1 | 0.863127 | 0.377173 | -1.19435 | 1.88E-06 | 3.75E-06 |
| LOXL1 | 10.90394 | 22.94507 | 1.073335 | 8.76E-81 | 4.87E-79 |
| MRGPRF | 1.122011 | 2.952022 | 1.395616 | 1.32E-85 | 9.20E-84 |
| RFX8 | 0.295233 | 0.619272 | 1.06872 | 1.71E-66 | 5.48E-65 |
| PIK3CG | 0.606248 | 1.515077 | 1.32141 | 1.80E-67 | 6.01E-66 |
| CRHBP | 0.114243 | 0.23799 | 1.058801 | 9.11E-43 | 1.17E-41 |
| ELN | 12.20828 | 29.52615 | 1.274133 | 2.53E-55 | 5.32E-54 |
| LTBP2 | 13.9244 | 28.85193 | 1.051053 | 5.51E-85 | 3.70E-83 |
| C11orf86 | 0.63662 | 0.145025 | -2.13414 | 0.0031484 | 0.0045662 |
| CCDC80 | 7.643277 | 27.21019 | 1.831884 | 6.66E-139 | 2.27E-135 |
| FOS | 50.71699 | 107.0935 | 1.078329 | 7.88E-27 | 5.56E-26 |
| FCRLA | 0.44016 | 0.93191 | 1.082163 | 2.66E-26 | 1.83E-25 |
| G0S2 | 9.575735 | 22.47006 | 1.230549 | 1.21E-19 | 6.02E-19 |
| EMX2 | 0.237473 | 0.533103 | 1.16665 | 9.06E-70 | 3.34E-68 |
| SPDYC | 10.80973 | 2.150604 | -2.32952 | 0.016524 | 0.021953 |
| IKZF1 | 1.308371 | 2.853188 | 1.124804 | 1.24E-55 | 2.63E-54 |
| MSR1 | 4.11026 | 8.492161 | 1.046902 | 2.23E-61 | 5.89E-60 |
| IL9R | 0.126774 | 0.266958 | 1.074357 | 8.44E-38 | 8.96E-37 |
| ASPA | 0.142219 | 0.32003 | 1.170093 | 6.07E-46 | 8.77E-45 |
| MMP9 | 35.4014 | 96.16491 | 1.441704 | 2.82E-08 | 6.57E-08 |
| FGL2 | 5.231744 | 12.13468 | 1.213772 | 2.79E-73 | 1.18E-71 |
| SOX17 | 1.000119 | 2.065383 | 1.046237 | 1.78E-41 | 2.15E-40 |
| CYP1A1 | 0.051081 | 0.161993 | 1.665078 | 0.0338304 | 0.0432459 |
| SYT13 | 18.41932 | 8.317551 | -1.14699 | 0.0001704 | 0.0002855 |
| COL15A1 | 13.44089 | 28.48949 | 1.083802 | 4.38E-98 | 5.42E-96 |
| DCSTAMP | 0.233525 | 0.520659 | 1.15676 | 4.34E-23 | 2.59E-22 |
| ISM1 | 2.820728 | 5.892396 | 1.062787 | 2.05E-61 | 5.43E-60 |
| SLC28A1 | 0.220922 | 0.104484 | -1.08025 | 2.46E-07 | 5.31E-07 |
| OLFML1 | 2.079349 | 5.550627 | 1.416519 | 3.42E-151 | 4.66E-147 |
| HSPB6 | 3.710983 | 8.787896 | 1.243716 | 2.08E-40 | 2.40E-39 |
| FBN1 | 9.729323 | 31.14749 | 1.678705 | 6.29E-122 | 3.90E-119 |
| HBQ1 | 0.194061 | 0.048835 | -1.99052 | 2.64E-12 | 8.37E-12 |
| FBLN1 | 22.1035 | 45.07335 | 1.028 | 6.82E-72 | 2.74E-70 |
| COL2A1 | 17.24563 | 7.485825 | -1.204 | 4.82E-05 | 8.50E-05 |
| ABCA10 | 0.174136 | 0.372506 | 1.09705 | 4.37E-25 | 2.86E-24 |
| CCR4 | 0.772335 | 1.997954 | 1.371225 | 3.99E-57 | 8.86E-56 |
| ABCA8 | 0.325726 | 1.159755 | 1.832089 | 2.57E-58 | 6.01E-57 |
| NID1 | 15.14302 | 30.85454 | 1.026829 | 8.84E-94 | 8.48E-92 |
| MEOX1 | 1.305116 | 2.825642 | 1.1144 | 1.01E-23 | 6.22E-23 |
| LGI2 | 0.553417 | 1.159045 | 1.066498 | 5.42E-64 | 1.59E-62 |
| TMEM130 | 0.379651 | 0.816724 | 1.105175 | 4.05E-52 | 7.61E-51 |
| CLMP | 4.252671 | 8.601555 | 1.016228 | 5.47E-90 | 4.47E-88 |
| RGS18 | 0.424804 | 0.869897 | 1.034048 | 2.43E-46 | 3.56E-45 |
| HSPB7 | 0.657398 | 1.52771 | 1.216531 | 1.86E-45 | 2.64E-44 |
| XPNPEP2 | 0.16879 | 0.507154 | 1.587197 | 1.78E-57 | 4.01E-56 |
| CCL11 | 0.854291 | 1.952433 | 1.192473 | 1.62E-39 | 1.81E-38 |
| CRYBA2 | 0.406816 | 0.063327 | -2.68349 | 1.28E-07 | 2.83E-07 |
| CR1 | 0.093569 | 0.221345 | 1.242196 | 2.18E-31 | 1.85E-30 |
| FCER1A | 1.550889 | 3.803031 | 1.294054 | 3.10E-40 | 3.57E-39 |
| CLEC17A | 0.175097 | 0.355558 | 1.021932 | 9.36E-22 | 5.24E-21 |
| HSPG2 | 11.76767 | 23.97381 | 1.026631 | 6.93E-74 | 3.03E-72 |
| GIMAP1 | 1.064281 | 2.135497 | 1.004693 | 3.85E-55 | 8.06E-54 |
| TMEM273 | 1.083738 | 2.351059 | 1.117295 | 1.03E-71 | 4.10E-70 |
| GAPT | 0.942569 | 1.992509 | 1.079916 | 2.84E-61 | 7.41E-60 |
| ONECUT2 | 0.353265 | 0.172239 | -1.03634 | 5.45E-05 | 9.58E-05 |
| GPR171 | 0.701645 | 1.40687 | 1.003676 | 7.49E-28 | 5.51E-27 |
| FADS6 | 0.187962 | 0.03217 | -2.54666 | 4.21E-06 | 8.18E-06 |
| MMP3 | 4.13344 | 11.5121 | 1.477737 | 7.00E-58 | 1.61E-56 |
| TNFAIP6 | 5.21416 | 10.47587 | 1.006563 | 7.45E-80 | 3.97E-78 |
| FGF16 | 0.084024 | 0.35558 | 2.081306 | 2.25E-65 | 6.93E-64 |
| FPR1 | 1.57852 | 3.302168 | 1.064841 | 6.32E-58 | 1.45E-56 |
| PCSK1N | 5.321413 | 1.926444 | -1.46587 | 0.0050613 | 0.0071562 |
| GPR34 | 2.604137 | 5.872052 | 1.17306 | 1.84E-82 | 1.10E-80 |
| NETO1 | 0.112712 | 0.302005 | 1.421927 | 8.78E-20 | 4.40E-19 |
| PDE1B | 0.535324 | 1.150859 | 1.104227 | 1.88E-83 | 1.18E-81 |
| GNG13 | 1.57514 | 0.746877 | -1.07654 | 1.24E-07 | 2.75E-07 |
| ELAVL2 | 1.002827 | 0.465933 | -1.10588 | 0.0005801 | 0.0009138 |
| GLIS3 | 0.57166 | 1.185039 | 1.051705 | 9.52E-71 | 3.56E-69 |
| CD28 | 0.607806 | 1.329639 | 1.129352 | 8.22E-49 | 1.35E-47 |
| PTPRC | 4.347183 | 10.36023 | 1.252903 | 1.59E-56 | 3.47E-55 |
| TRAT1 | 0.467365 | 1.021305 | 1.127792 | 3.88E-34 | 3.62E-33 |
| NEXN | 1.84433 | 3.758699 | 1.027137 | 7.87E-92 | 7.20E-90 |
| LRP1B | 0.594301 | 1.321729 | 1.15316 | 1.71E-10 | 4.77E-10 |
| DPP4 | 1.220216 | 3.360651 | 1.461605 | 6.65E-96 | 7.08E-94 |
| COL10A1 | 20.73639 | 65.12775 | 1.651108 | 6.72E-69 | 2.39E-67 |
| NR4A3 | 0.718483 | 1.672034 | 1.218577 | 8.77E-27 | 6.17E-26 |
| CSAG1 | 1.858309 | 0.722161 | -1.3636 | 0.0009171 | 0.0014134 |
| MYOCD | 0.050456 | 0.14622 | 1.535051 | 3.63E-35 | 3.52E-34 |
| HAS1 | 0.295874 | 0.662295 | 1.162493 | 2.52E-29 | 1.98E-28 |
| OMG | 0.115428 | 0.249924 | 1.11449 | 7.71E-21 | 4.08E-20 |
| FOLR2 | 4.280231 | 10.12757 | 1.242527 | 4.09E-48 | 6.49E-47 |
| GPR141 | 0.403162 | 0.904202 | 1.165287 | 5.64E-75 | 2.57E-73 |
| USH1C | 0.28401 | 0.049755 | -2.51303 | 0.000121 | 0.0002058 |
| RGS13 | 0.073889 | 0.168427 | 1.188698 | 3.06E-48 | 4.89E-47 |
| ABCD2 | 0.151483 | 0.347284 | 1.196963 | 9.51E-45 | 1.31E-43 |
| PRSS1 | 0.731499 | 0.309942 | -1.23886 | 0.0095357 | 0.0130585 |
| FOSB | 6.456835 | 17.07872 | 1.403301 | 2.25E-19 | 1.10E-18 |
| GPR183 | 4.885441 | 9.775188 | 1.000636 | 4.46E-45 | 6.24E-44 |
| VSTM4 | 1.341858 | 3.228903 | 1.266812 | 3.79E-108 | 7.49E-106 |
| ERG | 1.396234 | 2.796118 | 1.001885 | 2.62E-81 | 1.49E-79 |
| CD3G | 0.799281 | 1.798479 | 1.170003 | 2.38E-39 | 2.66E-38 |
| SLC8A2 | 0.561716 | 0.134909 | -2.05786 | 0.0029382 | 0.0042768 |
| CDK15 | 0.121763 | 0.300878 | 1.305103 | 1.89E-76 | 9.15E-75 |
| TH | 0.313661 | 0.156237 | -1.00547 | 0.0041813 | 0.0059747 |
| DKK3 | 7.791861 | 16.07082 | 1.044403 | 2.50E-73 | 1.07E-71 |
| TMEM151A | 0.293944 | 0.102215 | -1.52394 | 4.35E-06 | 8.42E-06 |
| COL6A1 | 92.11009 | 221.0722 | 1.263087 | 4.03E-86 | 2.89E-84 |
| KCNK7 | 0.361803 | 0.166032 | -1.12374 | 0.0144115 | 0.0192989 |
| GAB3 | 0.593926 | 1.221789 | 1.04064 | 2.40E-82 | 1.42E-80 |
| HPGDS | 0.951851 | 1.921941 | 1.013757 | 1.29E-58 | 3.05E-57 |
| CDH11 | 6.106419 | 15.94806 | 1.384982 | 9.31E-109 | 1.89E-106 |
| PDIA2 | 0.626893 | 0.132965 | -2.23717 | 6.39E-08 | 1.45E-07 |
| CA10 | 0.079976 | 0.180907 | 1.177609 | 6.32E-05 | 0.0001104 |
| FCAMR | 0.070184 | 0.216095 | 1.622442 | 0.000131 | 0.0002223 |
| PDE1A | 0.44934 | 0.963449 | 1.1004 | 1.35E-72 | 5.61E-71 |
| PLXNC1 | 2.835922 | 6.646447 | 1.228765 | 1.33E-109 | 2.79E-107 |
| AQP7 | 0.3754 | 1.195943 | 1.671647 | 3.67E-21 | 1.98E-20 |
| CCDC69 | 3.092658 | 6.287379 | 1.023612 | 1.15E-44 | 1.59E-43 |
| PRDM8 | 0.223918 | 0.492027 | 1.135768 | 7.60E-91 | 6.55E-89 |
| JCAD | 3.249377 | 6.918986 | 1.090398 | 5.05E-121 | 2.55E-118 |
| PPP1R16B | 1.301491 | 2.62134 | 1.010139 | 2.93E-43 | 3.82E-42 |
| TLR10 | 0.392204 | 0.816563 | 1.057961 | 7.82E-34 | 7.22E-33 |
| FHL1 | 3.614043 | 10.07657 | 1.479319 | 1.33E-84 | 8.86E-83 |
| THEMIS | 0.330149 | 0.750289 | 1.184331 | 4.54E-42 | 5.64E-41 |
| TNXB | 0.581486 | 1.754482 | 1.593229 | 1.02E-44 | 1.40E-43 |
| BNC2 | 0.51941 | 1.565434 | 1.591617 | 4.39E-125 | 3.52E-122 |
| METTL24 | 0.13533 | 0.345645 | 1.352803 | 1.96E-71 | 7.61E-70 |
| ZFPM2 | 0.519586 | 1.328142 | 1.353974 | 1.84E-110 | 4.25E-108 |
| CLEC9A | 0.124042 | 0.261971 | 1.078583 | 1.76E-28 | 1.33E-27 |
| SPARC | 313.594 | 841.5549 | 1.424159 | 7.50E-121 | 3.65E-118 |
| GLRA3 | 0.713087 | 1.775874 | 1.31638 | 0.0009748 | 0.0014985 |
| CD1C | 1.04237 | 2.351854 | 1.173932 | 5.71E-33 | 5.09E-32 |
| PENK | 0.200985 | 0.729125 | 1.859075 | 1.98E-28 | 1.49E-27 |
| SLCO2B1 | 3.184239 | 6.605633 | 1.052748 | 6.89E-76 | 3.25E-74 |
| FGF7 | 0.812633 | 2.265816 | 1.479355 | 6.88E-94 | 6.69E-92 |
| MYOG | 0.125442 | 0.046959 | -1.41756 | 5.15E-06 | 9.92E-06 |
| DOCK11 | 1.955569 | 4.166771 | 1.091341 | 2.15E-86 | 1.55E-84 |
| SLC38A5 | 1.527372 | 3.240481 | 1.085156 | 8.74E-65 | 2.62E-63 |
| TRARG1 | 1.245708 | 3.88618 | 1.641387 | 2.61E-22 | 1.49E-21 |
| COL3A1 | 324.7393 | 1083.604 | 1.738484 | 2.91E-121 | 1.59E-118 |
| ARMH4 | 0.546459 | 1.23478 | 1.17607 | 7.01E-101 | 1.00E-98 |
| NID2 | 3.359779 | 8.164409 | 1.280982 | 6.16E-89 | 4.71E-87 |
| BRSK2 | 0.617707 | 0.2455 | -1.3312 | 0.000185 | 0.0003086 |
| DPYSL5 | 0.579423 | 0.095344 | -2.60339 | 0.0001209 | 0.0002057 |
| PDGFRL | 5.91707 | 15.37584 | 1.37771 | 2.88E-100 | 4.04E-98 |
| NDN | 4.557532 | 9.94804 | 1.126159 | 4.59E-102 | 6.94E-100 |
| RBMS3 | 0.896809 | 2.149839 | 1.261355 | 2.49E-98 | 3.14E-96 |
| TEX19 | 0.34275 | 0.093289 | -1.87738 | 2.93E-06 | 5.77E-06 |
| C14orf180 | 0.126452 | 0.40188 | 1.668173 | 6.53E-18 | 2.96E-17 |
| KL | 0.270936 | 0.548787 | 1.018291 | 3.15E-47 | 4.80E-46 |
| SEMA3D | 0.36071 | 0.90521 | 1.327414 | 2.46E-76 | 1.18E-74 |
| PRSS33 | 1.40906 | 0.376097 | -1.90556 | 0.0066332 | 0.0092507 |
| CYP2A13 | 0.424438 | 0.064084 | -2.72752 | 1.42E-05 | 2.62E-05 |
| CDK5R2 | 0.6159 | 0.062181 | -3.30816 | 0.0015311 | 0.0022992 |
| HGF | 0.432033 | 1.08386 | 1.326967 | 1.62E-90 | 1.37E-88 |
| SLITRK1 | 0.369776 | 0.095107 | -1.95903 | 0.0008467 | 0.0013103 |
| NR2F1 | 3.523928 | 7.767959 | 1.140351 | 2.40E-52 | 4.58E-51 |
| KRTDAP | 8.057161 | 0.848034 | -3.24808 | 6.32E-05 | 0.0001104 |
| ZEB2 | 1.105562 | 2.643189 | 1.2575 | 3.02E-122 | 1.96E-119 |
| IGDCC4 | 0.411694 | 0.846382 | 1.039737 | 2.62E-61 | 6.85E-60 |
| ALPK2 | 0.156926 | 0.341379 | 1.121292 | 2.21E-48 | 3.55E-47 |
| TENM4 | 0.667787 | 1.417089 | 1.08547 | 9.80E-85 | 6.55E-83 |
| TBX5 | 0.512789 | 1.149073 | 1.164034 | 1.15E-94 | 1.16E-92 |
| PLA2G5 | 0.270029 | 0.588359 | 1.123585 | 1.84E-53 | 3.65E-52 |
| KERA | 0.351008 | 0.916933 | 1.385312 | 1.83E-85 | 1.27E-83 |
| TEX11 | 0.067862 | 0.144437 | 1.089771 | 1.30E-47 | 2.02E-46 |
| VGLL3 | 0.853953 | 2.007138 | 1.232912 | 3.06E-89 | 2.39E-87 |
| PTGER4 | 1.717222 | 3.450451 | 1.006708 | 1.04E-70 | 3.86E-69 |
| LAMA2 | 2.042591 | 5.983328 | 1.550548 | 7.83E-89 | 5.96E-87 |
| C1QTNF7 | 0.225313 | 0.737812 | 1.711324 | 1.92E-62 | 5.29E-61 |
| MSLN | 16.59692 | 5.246696 | -1.66143 | 1.76E-05 | 3.23E-05 |
| VMO1 | 2.471929 | 7.580185 | 1.616596 | 0.0001579 | 0.0002656 |
| LIPE | 2.510099 | 5.617256 | 1.162121 | 3.85E-13 | 1.28E-12 |
| PNOC | 0.249565 | 0.542625 | 1.120539 | 2.23E-36 | 2.27E-35 |
| CIDEC | 1.874897 | 6.984949 | 1.897438 | 1.25E-17 | 5.56E-17 |
| CA4 | 0.117985 | 0.401228 | 1.765818 | 7.79E-17 | 3.29E-16 |
| ACKR1 | 6.001523 | 17.66765 | 1.55771 | 8.69E-30 | 6.93E-29 |
| CTSG | 0.815309 | 2.27096 | 1.477884 | 2.96E-41 | 3.56E-40 |
| OLFML2B | 12.77747 | 31.52514 | 1.3029 | 5.35E-112 | 1.46E-109 |
| FNDC1 | 7.519235 | 16.8735 | 1.166101 | 1.07E-76 | 5.19E-75 |
| SLC9A9 | 1.159506 | 2.338104 | 1.011829 | 3.61E-84 | 2.32E-82 |
| CILP2 | 2.367961 | 5.830202 | 1.299901 | 5.28E-56 | 1.13E-54 |
| MMP2 | 49.23939 | 154.6391 | 1.65102 | 1.45E-130 | 1.64E-127 |
| C1QTNF6 | 4.546405 | 9.206562 | 1.017936 | 5.77E-80 | 3.12E-78 |
| TMEM132C | 0.208574 | 0.730111 | 1.807554 | 1.87E-34 | 1.77E-33 |
| CHIT1 | 1.203969 | 4.21764 | 1.808637 | 1.33E-15 | 5.19E-15 |
| CD300LB | 0.272808 | 0.638458 | 1.226704 | 9.28E-49 | 1.51E-47 |
| FAM131C | 0.430791 | 0.167547 | -1.36242 | 3.56E-11 | 1.04E-10 |
| SPARCL1 | 46.72172 | 112.5831 | 1.268825 | 4.85E-90 | 3.98E-88 |
| LMOD1 | 4.142881 | 8.585127 | 1.051205 | 1.25E-70 | 4.65E-69 |
| DNASE1L3 | 0.273847 | 0.565335 | 1.045734 | 1.31E-29 | 1.03E-28 |
| CD84 | 1.549932 | 3.675575 | 1.245765 | 2.24E-64 | 6.61E-63 |
| NOX4 | 1.082239 | 2.569023 | 1.247201 | 3.16E-95 | 3.32E-93 |
| CD96 | 1.047288 | 2.173196 | 1.053159 | 1.92E-37 | 2.02E-36 |
| DBX2 | 0.050991 | 0.137255 | 1.428541 | 1.19E-44 | 1.63E-43 |
| SFRP4 | 14.03829 | 52.13726 | 1.892947 | 3.52E-102 | 5.39E-100 |
| ROPN1B | 1.964327 | 0.722188 | -1.44359 | 0.0358067 | 0.0456139 |
| PPM1E | 0.796606 | 0.264409 | -1.59109 | 8.61E-12 | 2.63E-11 |
| TMEM213 | 0.89863 | 0.386297 | -1.21802 | 0.028882 | 0.0372206 |
| MAT1A | 1.284804 | 0.475322 | -1.43457 | 7.23E-08 | 1.63E-07 |
| ERVFRD-1 | 0.059861 | 0.241165 | 2.010339 | 2.93E-28 | 2.19E-27 |
| PCDHGA12 | 0.332067 | 0.694477 | 1.064451 | 2.15E-79 | 1.13E-77 |
| HTRA1 | 69.47871 | 179.4904 | 1.369264 | 3.78E-110 | 8.59E-108 |
| SPON1 | 6.180065 | 17.43989 | 1.496697 | 1.84E-109 | 3.79E-107 |
| LRTM2 | 0.738218 | 0.197649 | -1.90111 | 0.0004144 | 0.0006637 |
| TLR4 | 2.210176 | 5.013849 | 1.181757 | 2.02E-99 | 2.62E-97 |
| TNFSF4 | 1.396715 | 2.85976 | 1.033856 | 1.64E-58 | 3.86E-57 |
| ENHO | 7.982484 | 3.03753 | -1.39394 | 4.50E-13 | 1.50E-12 |
| ADIPOQ | 2.517934 | 10.42695 | 2.050005 | 2.04E-25 | 1.36E-24 |
| CD1E | 0.705914 | 1.503701 | 1.090953 | 7.94E-28 | 5.82E-27 |
| LEP | 0.517019 | 2.724689 | 2.397801 | 3.63E-18 | 1.67E-17 |
| DMP1 | 0.07493 | 0.25856 | 1.786889 | 0.0004391 | 0.0007013 |
| IL21R | 0.670757 | 1.380945 | 1.041795 | 2.57E-52 | 4.90E-51 |
| TMEM179 | 0.503431 | 0.165688 | -1.60332 | 0.0382474 | 0.0485505 |
| DIO2 | 2.86724 | 7.025532 | 1.292945 | 1.25E-57 | 2.84E-56 |
| CPN2 | 0.103793 | 0.213179 | 1.038351 | 3.79E-47 | 5.73E-46 |
| ABCA6 | 0.232779 | 0.737369 | 1.663423 | 7.90E-94 | 7.63E-92 |
| IL7R | 2.705323 | 7.052137 | 1.38226 | 5.23E-49 | 8.65E-48 |
| FILIP1L | 4.282962 | 10.10513 | 1.238406 | 4.26E-106 | 7.64E-104 |
| ASPHD1 | 3.340523 | 1.445137 | -1.20887 | 9.50E-09 | 2.31E-08 |
| IFFO1 | 1.316925 | 2.634911 | 1.000581 | 1.58E-92 | 1.48E-90 |
| EBF1 | 1.234074 | 2.692531 | 1.125534 | 1.17E-88 | 8.77E-87 |
| SCN7A | 0.169566 | 0.592304 | 1.804488 | 8.75E-53 | 1.69E-51 |
| MSLNL | 0.249038 | 0.106035 | -1.23183 | 2.25E-09 | 5.75E-09 |
| PEX5L | 0.78676 | 0.364311 | -1.11075 | 7.67E-05 | 0.0001329 |
| AXL | 7.544504 | 15.73924 | 1.060867 | 2.55E-128 | 2.67E-125 |
| F2R | 12.75162 | 26.56743 | 1.058978 | 1.81E-88 | 1.35E-86 |
| GIMAP5 | 0.161136 | 0.36366 | 1.174315 | 1.43E-43 | 1.91E-42 |
| APBB1IP | 2.900419 | 5.802367 | 1.00038 | 7.33E-73 | 3.06E-71 |
| GPIHBP1 | 0.868029 | 2.331594 | 1.425502 | 2.65E-34 | 2.49E-33 |
| FST | 4.664616 | 9.347751 | 1.002861 | 4.32E-58 | 9.99E-57 |
| HSD11B1 | 1.263901 | 3.244996 | 1.360333 | 2.67E-39 | 2.98E-38 |
| COL6A3 | 36.53153 | 100.1673 | 1.455197 | 1.86E-118 | 7.45E-116 |
| MAGEL2 | 0.143153 | 0.401545 | 1.488 | 1.16E-99 | 1.55E-97 |
| KCNJ15 | 0.205938 | 0.418403 | 1.022683 | 9.33E-66 | 2.90E-64 |
| FAM57B | 0.721764 | 0.313405 | -1.2035 | 8.02E-09 | 1.96E-08 |
| FAT4 | 0.469712 | 1.077311 | 1.197586 | 1.90E-86 | 1.38E-84 |
| CNTN1 | 1.185433 | 2.717157 | 1.196684 | 1.39E-62 | 3.88E-61 |
| COL5A3 | 5.186647 | 10.89559 | 1.07087 | 2.45E-57 | 5.48E-56 |
| PDPN | 3.915047 | 9.277351 | 1.244683 | 4.91E-100 | 6.76E-98 |
| CELF4 | 0.483642 | 0.118136 | -2.0335 | 2.52E-08 | 5.91E-08 |
| COL6A2 | 102.2395 | 240.0801 | 1.231563 | 7.41E-83 | 4.52E-81 |
| DPEP1 | 0.411794 | 0.828424 | 1.008447 | 2.21E-42 | 2.77E-41 |
| CLEC4C | 0.073875 | 0.198618 | 1.426844 | 2.38E-16 | 9.79E-16 |
| CD27 | 2.889626 | 5.795032 | 1.003934 | 2.49E-32 | 2.17E-31 |
| CD52 | 16.31754 | 47.60899 | 1.544811 | 2.82E-34 | 2.65E-33 |
| TBX15 | 1.167986 | 2.478752 | 1.085591 | 2.70E-98 | 3.37E-96 |
| EGR2 | 3.994408 | 8.255645 | 1.047399 | 1.44E-43 | 1.91E-42 |
| COL1A1 | 369.4535 | 1119.203 | 1.599007 | 2.84E-104 | 4.84E-102 |
| THBS4 | 8.831472 | 20.86402 | 1.240292 | 1.08E-46 | 1.61E-45 |
| AKAP12 | 2.176602 | 6.102936 | 1.487426 | 7.20E-100 | 9.81E-98 |
| ADAM12 | 5.730434 | 17.33328 | 1.596828 | 9.95E-110 | 2.15E-107 |
| AOC3 | 4.39103 | 11.55351 | 1.3957 | 7.33E-66 | 2.29E-64 |
| VCAN | 16.43086 | 46.36692 | 1.496688 | 1.49E-111 | 3.75E-109 |
| SPN | 1.226262 | 2.609332 | 1.089414 | 1.79E-55 | 3.80E-54 |
| ECM2 | 2.317304 | 7.296785 | 1.654814 | 1.27E-130 | 1.57E-127 |
| LRRC15 | 11.05629 | 39.90075 | 1.851549 | 8.07E-98 | 9.73E-96 |
| PLA2G2D | 1.464664 | 3.172289 | 1.114954 | 1.64E-14 | 5.93E-14 |
| TEK | 1.801684 | 3.771041 | 1.065617 | 3.50E-71 | 1.34E-69 |
| PDE2A | 0.810101 | 1.653915 | 1.029711 | 1.59E-48 | 2.57E-47 |
| THY1 | 17.42375 | 37.19547 | 1.094072 | 2.03E-93 | 1.93E-91 |
| BHLHE22 | 0.357794 | 0.938764 | 1.391631 | 3.16E-85 | 2.17E-83 |
| FLI1 | 1.627536 | 3.352751 | 1.042655 | 1.90E-99 | 2.48E-97 |
| PTH1R | 0.405946 | 1.163785 | 1.519464 | 1.34E-82 | 8.09E-81 |
| CCR2 | 0.979976 | 2.208896 | 1.172507 | 1.34E-47 | 2.08E-46 |
| SFRP2 | 127.1853 | 383.8855 | 1.593744 | 5.13E-105 | 8.97E-103 |
| SALL1 | 0.22786 | 0.473374 | 1.054834 | 3.81E-71 | 1.46E-69 |
| JAM2 | 1.820294 | 3.960382 | 1.121468 | 4.34E-86 | 3.08E-84 |
| GZMK | 2.381497 | 5.85686 | 1.298259 | 3.16E-36 | 3.19E-35 |
| P4HA3 | 1.500773 | 3.783474 | 1.334006 | 4.19E-97 | 4.87E-95 |
| TREML2 | 0.089237 | 0.18691 | 1.066626 | 7.57E-28 | 5.56E-27 |
| SH3GL3 | 0.168928 | 0.061961 | -1.44698 | 2.26E-05 | 4.10E-05 |
| SRPX | 5.308512 | 11.52607 | 1.118521 | 1.90E-91 | 1.69E-89 |
| FCER2 | 0.33104 | 0.692962 | 1.065769 | 7.43E-23 | 4.37E-22 |
| HEG1 | 5.630079 | 12.7833 | 1.183033 | 1.58E-120 | 7.18E-118 |
| DLL3 | 0.410347 | 0.150967 | -1.44261 | 1.91E-08 | 4.54E-08 |
| CLEC4D | 0.066902 | 0.233061 | 1.800573 | 2.88E-10 | 7.91E-10 |
| ENPP2 | 3.263698 | 6.587601 | 1.013246 | 4.62E-48 | 7.31E-47 |
| KCNA3 | 0.292865 | 0.756513 | 1.369131 | 3.44E-47 | 5.23E-46 |
| PGPEP1L | 0.14014 | 0.064901 | -1.11055 | 4.08E-07 | 8.65E-07 |
| SRPX2 | 5.810786 | 12.16134 | 1.065497 | 2.22E-84 | 1.46E-82 |
| COL1A2 | 226.9273 | 718.519 | 1.662796 | 9.62E-115 | 3.20E-112 |
| SH2D1A | 0.954469 | 2.099437 | 1.137232 | 3.07E-34 | 2.88E-33 |
| LMO1 | 0.399163 | 0.145906 | -1.45194 | 0.0005621 | 0.000887 |
| GAS1 | 6.177104 | 14.34576 | 1.215622 | 2.61E-92 | 2.42E-90 |
| LCN6 | 0.071647 | 0.179251 | 1.323012 | 1.53E-21 | 8.46E-21 |
| NUGGC | 0.18969 | 0.421507 | 1.151912 | 1.06E-41 | 1.30E-40 |
| TCEAL7 | 0.988609 | 2.096206 | 1.084308 | 4.16E-71 | 1.58E-69 |
| CST7 | 4.675994 | 9.458763 | 1.016379 | 1.80E-48 | 2.91E-47 |
| CCNE1 | 5.015592 | 2.423079 | -1.04958 | 3.58E-12 | 1.13E-11 |
| GLDC | 2.722604 | 0.932762 | -1.54541 | 6.48E-06 | 1.24E-05 |
| C16orf54 | 1.060457 | 2.296265 | 1.114602 | 9.91E-43 | 1.27E-41 |
| MS4A6E | 0.026178 | 0.150917 | 2.527311 | 7.44E-11 | 2.13E-10 |
