# Supplementary material for: CCL19 has potential to be a potential prognostic biomarker and a modulator of tumor immune microenvironment (TIME) of breast cancer: a comprehensive analysis based on TCGA database
Source: Aging (Albany NY). 2022 May 12;14(9):4158–75. doi: 10.18632/aging.204081 (PMC9134962; doi:10.18632/aging.204081)
Supplement: Supplementary Table 3 [file aging-14-204081-s004.docx]

**Supplementary Table 3. DEGs regarding immune scores.**

| **Gene** | **conMean** | **treatMean** | **logFC** | **pValue** | **FDR** |
| --- | --- | --- | --- | --- | --- |
| CD180 | 0.923218 | 2.267056 | 1.296076 | 1.84E-92 | 7.74E-91 |
| JAML | 0.454751 | 1.883655 | 2.050387 | 2.48E-128 | 3.48E-126 |
| SIT1 | 0.679828 | 3.775543 | 2.473443 | 6.56E-133 | 1.42E-130 |
| ZDHHC22 | 0.313516 | 0.076859 | -2.02825 | 0.000305188 | 0.00056634 |
| MMP8 | 0.071974 | 0.189003 | 1.392872 | 2.00E-12 | 9.88E-12 |
| STAP1 | 0.318195 | 1.347672 | 2.082485 | 2.68E-81 | 9.11E-80 |
| LYL1 | 0.906231 | 1.961374 | 1.113915 | 7.33E-65 | 1.78E-63 |
| LY86 | 3.964073 | 8.520141 | 1.103894 | 3.83E-99 | 1.86E-97 |
| CAPN6 | 2.853097 | 6.362776 | 1.157128 | 1.86E-15 | 1.18E-14 |
| KCNC2 | 2.276754 | 0.847535 | -1.42563 | 1.25E-05 | 2.80E-05 |
| NCKAP1L | 1.710115 | 5.409171 | 1.661315 | 3.07E-144 | 1.73E-141 |
| KLK14 | 2.911496 | 13.33214 | 2.195075 | 0.00070341 | 0.001244005 |
| FOXP3 | 1.214136 | 3.761588 | 1.631412 | 8.39E-101 | 4.24E-99 |
| PLA1A | 1.119741 | 2.662181 | 1.249443 | 8.95E-46 | 1.53E-44 |
| CMA1 | 0.442439 | 0.946199 | 1.096666 | 3.45E-16 | 2.31E-15 |
| CYSLTR2 | 0.206466 | 0.435009 | 1.075142 | 1.24E-63 | 2.95E-62 |
| EVI2B | 4.121795 | 13.88956 | 1.752656 | 1.47E-143 | 7.39E-141 |
| CAMK4 | 0.244175 | 0.506745 | 1.053346 | 9.95E-60 | 2.16E-58 |
| GBP6 | 0.17854 | 0.41514 | 1.21735 | 5.58E-40 | 8.34E-39 |
| IL10RA | 1.920768 | 6.628221 | 1.786939 | 1.23E-147 | 1.15E-144 |
| APOBEC3C | 6.875806 | 14.24769 | 1.051127 | 1.26E-82 | 4.38E-81 |
| TXK | 0.113757 | 0.356201 | 1.64673 | 4.28E-63 | 1.00E-61 |
| GPSM3 | 8.001891 | 18.81591 | 1.23354 | 1.94E-109 | 1.25E-107 |
| CYTH4 | 1.5309 | 4.247642 | 1.472282 | 2.73E-139 | 1.10E-136 |
| EGFL6 | 1.161816 | 3.211595 | 1.466908 | 7.89E-37 | 1.12E-35 |
| MFNG | 2.112025 | 5.048898 | 1.257342 | 1.46E-106 | 8.52E-105 |
| HLA-DMB | 5.020215 | 14.91674 | 1.571111 | 2.42E-145 | 1.62E-142 |
| TOX | 0.514343 | 1.547943 | 1.58955 | 6.61E-90 | 2.62E-88 |
| JCHAIN | 65.39691 | 164.1168 | 1.327428 | 3.25E-48 | 5.81E-47 |
| KRT86 | 1.90328 | 4.547846 | 1.256696 | 4.45E-14 | 2.51E-13 |
| ITK | 0.235435 | 1.404401 | 2.576556 | 3.01E-126 | 3.78E-124 |
| KCNJ3 | 17.76675 | 6.849746 | -1.37506 | 3.77E-08 | 1.16E-07 |
| CD48 | 1.993378 | 8.902562 | 2.159005 | 2.92E-150 | 3.42E-147 |
| HLA-DMA | 15.7611 | 42.04272 | 1.415488 | 1.93E-131 | 3.64E-129 |
| CD2 | 3.267924 | 18.20224 | 2.47767 | 2.03E-139 | 8.39E-137 |
| AKNA | 2.23087 | 5.160563 | 1.209922 | 5.06E-103 | 2.66E-101 |
| MS4A6A | 4.006376 | 10.34416 | 1.368447 | 1.23E-129 | 1.94E-127 |
| CPB1 | 445.9284 | 157.0165 | -1.5059 | 4.84E-09 | 1.66E-08 |
| FCGR2B | 0.839058 | 1.894503 | 1.174977 | 3.30E-87 | 1.24E-85 |
| FCRL2 | 0.046203 | 0.383621 | 3.053624 | 1.23E-72 | 3.42E-71 |
| C1QB | 37.86424 | 118.4038 | 1.644807 | 1.84E-123 | 2.16E-121 |
| TESPA1 | 0.288729 | 1.213298 | 2.071146 | 1.18E-112 | 8.65E-111 |
| PHGR1 | 3.523687 | 1.396747 | -1.33501 | 6.55E-08 | 1.96E-07 |
| PRR4 | 2.115318 | 0.593166 | -1.83437 | 0.012551553 | 0.018371028 |
| SNX20 | 0.603306 | 2.565342 | 2.08819 | 2.42E-155 | 1.13E-151 |
| KLHL6 | 0.439769 | 1.47881 | 1.749619 | 2.57E-124 | 3.06E-122 |
| CARD16 | 1.94086 | 4.310169 | 1.151049 | 4.97E-105 | 2.79E-103 |
| ARRDC5 | 0.08801 | 0.2573 | 1.547713 | 7.03E-84 | 2.49E-82 |
| SCARA5 | 0.500829 | 1.158493 | 1.209861 | 1.38E-30 | 1.67E-29 |
| TNFSF13B | 2.564043 | 7.521655 | 1.55263 | 1.02E-107 | 6.14E-106 |
| LRP2 | 11.11203 | 5.418324 | -1.0362 | 2.66E-06 | 6.50E-06 |
| FAM20A | 1.341532 | 3.158474 | 1.235346 | 1.05E-75 | 3.13E-74 |
| GZMH | 0.859225 | 4.143196 | 2.269635 | 3.24E-109 | 2.04E-107 |
| MS4A8 | 1.168612 | 0.569382 | -1.03733 | 1.10E-09 | 4.09E-09 |
| GSDMA | 0.284259 | 0.633156 | 1.155354 | 1.50E-38 | 2.17E-37 |
| C1QA | 39.3272 | 126.007 | 1.679905 | 2.54E-126 | 3.22E-124 |
| RASSF4 | 1.974829 | 4.945284 | 1.324325 | 1.63E-107 | 9.72E-106 |
| RAB33A | 0.457517 | 1.088278 | 1.250149 | 6.86E-95 | 3.04E-93 |
| IL2RG | 3.686384 | 20.08206 | 2.445629 | 3.08E-147 | 2.55E-144 |
| PHKG1 | 0.632912 | 1.905379 | 1.59 | 1.76E-08 | 5.68E-08 |
| KRT33A | 0.258839 | 0.119118 | -1.11967 | 0.000106028 | 0.000209065 |
| PRAM1 | 0.425077 | 0.936864 | 1.140115 | 1.08E-79 | 3.50E-78 |
| VNN2 | 0.470908 | 1.396895 | 1.568708 | 3.19E-95 | 1.42E-93 |
| LAT2 | 1.968434 | 4.103668 | 1.059865 | 4.85E-105 | 2.74E-103 |
| KLRF1 | 0.245928 | 0.509608 | 1.051152 | 9.09E-45 | 1.52E-43 |
| SELL | 4.666611 | 12.14635 | 1.380076 | 1.93E-98 | 9.20E-97 |
| CD226 | 0.130759 | 0.416363 | 1.670927 | 7.50E-109 | 4.64E-107 |
| RUNDC3A | 1.686817 | 0.455011 | -1.89033 | 0.011814994 | 0.017368956 |
| WDFY4 | 0.51575 | 1.689513 | 1.711863 | 5.49E-122 | 6.07E-120 |
| CXCL11 | 2.802227 | 14.09294 | 2.330327 | 7.60E-83 | 2.66E-81 |
| AC136428.1 | 0.234622 | 1.383194 | 2.559592 | 3.94E-44 | 6.48E-43 |
| UGT2B4 | 5.806173 | 1.320721 | -2.13626 | 7.98E-05 | 0.000160419 |
| FUT7 | 0.100573 | 0.511422 | 2.346269 | 9.95E-112 | 6.99E-110 |
| FCGR3B | 0.318964 | 0.929114 | 1.542463 | 3.58E-21 | 3.23E-20 |
| C2 | 3.045511 | 6.458725 | 1.084565 | 4.50E-83 | 1.59E-81 |
| MAP1LC3C | 0.282623 | 0.599523 | 1.084936 | 3.07E-31 | 3.79E-30 |
| MYT1 | 1.080672 | 0.296398 | -1.86632 | 7.12E-14 | 3.94E-13 |
| TAGAP | 1.292278 | 4.073448 | 1.656334 | 3.61E-117 | 3.25E-115 |
| LILRB2 | 0.793215 | 2.578952 | 1.701001 | 4.89E-142 | 2.37E-139 |
| CCL25 | 0.072395 | 0.280127 | 1.95212 | 1.17E-22 | 1.12E-21 |
| HEPHL1 | 0.132381 | 0.278985 | 1.075491 | 0.003853289 | 0.006091647 |
| CD14 | 23.42823 | 50.64476 | 1.112165 | 1.10E-73 | 3.14E-72 |
| FASLG | 0.228906 | 1.195921 | 2.385295 | 5.02E-109 | 3.15E-107 |
| ZNF831 | 0.074014 | 0.430974 | 2.54173 | 6.05E-115 | 4.92E-113 |
| SLC22A3 | 0.380528 | 0.802379 | 1.07628 | 4.53E-44 | 7.38E-43 |
| STC2 | 126.5433 | 58.23927 | -1.11957 | 1.40E-10 | 5.73E-10 |
| TMEM145 | 2.557519 | 1.060618 | -1.26984 | 4.98E-13 | 2.58E-12 |
| SIGLEC9 | 0.791724 | 1.740853 | 1.136724 | 3.69E-97 | 1.71E-95 |
| IL4I1 | 2.102126 | 7.333079 | 1.80257 | 1.47E-95 | 6.62E-94 |
| PLAC8 | 0.24269 | 1.19418 | 2.298837 | 1.33E-93 | 5.72E-92 |
| ABI3BP | 1.501667 | 3.523067 | 1.230267 | 3.57E-48 | 6.37E-47 |
| MZB1 | 8.014039 | 16.87954 | 1.074674 | 2.66E-29 | 3.11E-28 |
| IL18RAP | 0.096706 | 0.440109 | 2.18618 | 1.65E-107 | 9.85E-106 |
| SPI1 | 6.383825 | 17.16941 | 1.427347 | 8.21E-136 | 2.35E-133 |
| TNFSF8 | 0.603403 | 1.616849 | 1.421992 | 1.24E-100 | 6.24E-99 |
| TRH | 21.76325 | 4.159224 | -2.38751 | 1.41E-08 | 4.59E-08 |
| TLR8 | 0.294874 | 1.295108 | 2.1349 | 6.31E-113 | 4.64E-111 |
| BIN2 | 1.374456 | 4.275993 | 1.637399 | 4.16E-147 | 3.25E-144 |
| EPHB6 | 1.133368 | 2.317757 | 1.032113 | 1.02E-40 | 1.55E-39 |
| FCN1 | 0.258923 | 1.252982 | 2.274769 | 7.14E-98 | 3.37E-96 |
| GYPC | 4.160021 | 8.400418 | 1.01387 | 3.04E-74 | 8.75E-73 |
| FCRLB | 4.746496 | 1.634782 | -1.53776 | 0.000804609 | 0.001411607 |
| XIRP1 | 0.150777 | 0.570507 | 1.91983 | 2.73E-63 | 6.44E-62 |
| WDR17 | 0.523556 | 0.250157 | -1.06551 | 0.000331626 | 0.000612712 |
| DOCK8 | 2.12299 | 4.302708 | 1.019147 | 8.52E-67 | 2.16E-65 |
| TRPM8 | 0.126272 | 0.303012 | 1.262841 | 5.15E-17 | 3.67E-16 |
| CCL23 | 0.150213 | 0.460944 | 1.617577 | 9.33E-68 | 2.40E-66 |
| HCRTR2 | 0.057473 | 0.163766 | 1.51067 | 4.23E-05 | 8.82E-05 |
| HLA-B | 356.5416 | 753.8618 | 1.08023 | 9.35E-75 | 2.70E-73 |
| HLA-G | 1.797009 | 4.020492 | 1.161774 | 8.94E-27 | 9.71E-26 |
| FGR | 1.490694 | 4.220716 | 1.501503 | 1.88E-130 | 3.18E-128 |
| PRKCB | 0.328635 | 1.674161 | 2.348878 | 1.63E-125 | 2.02E-123 |
| SCIMP | 0.550122 | 1.702075 | 1.629471 | 3.86E-127 | 4.97E-125 |
| IL16 | 0.961266 | 2.619402 | 1.44623 | 1.92E-107 | 1.14E-105 |
| IDO1 | 1.502916 | 11.2046 | 2.898254 | 1.17E-93 | 5.06E-92 |
| CD1B | 0.112639 | 0.674678 | 2.582491 | 2.28E-69 | 5.94E-68 |
| IGSF6 | 2.401786 | 6.026497 | 1.327212 | 6.24E-109 | 3.88E-107 |
| APOBEC3A | 0.241764 | 1.030698 | 2.09195 | 3.45E-53 | 6.70E-52 |
| CTSE | 0.149798 | 0.46891 | 1.646294 | 2.03E-15 | 1.28E-14 |
| KRT1 | 0.896345 | 14.31791 | 3.997623 | 4.91E-07 | 1.32E-06 |
| WNT10A | 0.389112 | 0.853947 | 1.13396 | 3.53E-75 | 1.03E-73 |
| HLA-DRB5 | 50.58831 | 166.2858 | 1.716789 | 1.98E-90 | 8.01E-89 |
| IL12RB1 | 0.370701 | 1.513641 | 2.029696 | 2.33E-151 | 2.98E-148 |
| NRROS | 1.504373 | 3.065187 | 1.026812 | 2.99E-89 | 1.16E-87 |
| WIPF1 | 4.611608 | 10.61376 | 1.202594 | 8.91E-115 | 7.19E-113 |
| LCK | 1.070292 | 5.538986 | 2.371617 | 1.33E-128 | 1.94E-126 |
| PIK3AP1 | 2.441858 | 5.897544 | 1.272135 | 2.38E-98 | 1.13E-96 |
| C11orf21 | 0.109329 | 0.472701 | 2.112251 | 5.49E-108 | 3.32E-106 |
| KRT81 | 28.14517 | 62.72143 | 1.156071 | 1.33E-08 | 4.36E-08 |
| PTPN7 | 0.68649 | 3.023 | 2.138671 | 1.55E-151 | 2.20E-148 |
| SLA | 1.546573 | 4.346237 | 1.490692 | 1.19E-132 | 2.45E-130 |
| FABP4 | 24.6941 | 59.31724 | 1.264285 | 2.58E-14 | 1.49E-13 |
| KCNH6 | 0.232175 | 0.06442 | -1.84962 | 0.004565375 | 0.007137081 |
| HLA-DQB2 | 4.899825 | 14.76219 | 1.591105 | 6.75E-72 | 1.84E-70 |
| P2RY10 | 0.821964 | 2.572365 | 1.645949 | 1.49E-90 | 6.08E-89 |
| PRTN3 | 0.396023 | 0.189913 | -1.06025 | 1.39E-07 | 4.00E-07 |
| MPPED1 | 0.213101 | 0.10323 | -1.04567 | 0.004904686 | 0.007639479 |
| ZBED2 | 0.157768 | 1.000272 | 2.664512 | 1.97E-93 | 8.45E-92 |
| GPR150 | 0.074235 | 0.173066 | 1.221148 | 3.37E-67 | 8.65E-66 |
| ROS1 | 0.049546 | 0.304964 | 2.621794 | 0.000381947 | 0.000700618 |
| TIMD4 | 0.165441 | 0.667266 | 2.011941 | 4.89E-64 | 1.17E-62 |
| TPSD1 | 1.453669 | 2.99252 | 1.041662 | 0.007212538 | 0.010961966 |
| NCR3 | 0.1886 | 0.874446 | 2.213042 | 3.10E-101 | 1.58E-99 |
| FAM234B | 23.31606 | 10.49611 | -1.15147 | 9.31E-21 | 8.25E-20 |
| CLEC10A | 0.673562 | 3.153876 | 2.227244 | 5.33E-86 | 1.95E-84 |
| PI16 | 2.364667 | 5.827563 | 1.301256 | 1.09E-24 | 1.12E-23 |
| CPA1 | 0.029277 | 0.064084 | 1.130207 | 4.85E-07 | 1.30E-06 |
| GPR55 | 0.126536 | 0.386292 | 1.610137 | 1.29E-75 | 3.84E-74 |
| BTLA | 0.115175 | 0.778779 | 2.757384 | 4.12E-113 | 3.04E-111 |
| MPEG1 | 4.347505 | 13.39243 | 1.623158 | 1.69E-122 | 1.89E-120 |
| RAC2 | 8.72723 | 24.21623 | 1.472379 | 2.06E-107 | 1.22E-105 |
| SPIB | 0.403031 | 2.873262 | 2.833728 | 8.73E-85 | 3.15E-83 |
| CPVL | 4.060313 | 9.312407 | 1.197563 | 6.89E-105 | 3.84E-103 |
| CD69 | 1.005038 | 3.694088 | 1.877968 | 1.89E-102 | 9.84E-101 |
| CHRNB2 | 1.048849 | 0.351364 | -1.57777 | 7.04E-05 | 0.000142546 |
| HLA-DOB | 1.026408 | 3.958869 | 1.947484 | 3.49E-80 | 1.15E-78 |
| PRDM1 | 2.225895 | 4.571798 | 1.038376 | 1.90E-78 | 5.99E-77 |
| VSIG4 | 4.682497 | 11.34758 | 1.277035 | 2.75E-63 | 6.47E-62 |
| C8orf86 | 0.292448 | 0.103625 | -1.49682 | 8.89E-15 | 5.33E-14 |
| GP1BA | 0.307837 | 0.876996 | 1.510405 | 8.25E-40 | 1.23E-38 |
| SPOCK2 | 2.429022 | 8.376377 | 1.785951 | 4.44E-117 | 3.95E-115 |
| LIMD2 | 2.61036 | 6.798175 | 1.380899 | 3.40E-85 | 1.23E-83 |
| CST9L | 0.600391 | 0.263576 | -1.18768 | 5.98E-10 | 2.27E-09 |
| TBX10 | 0.432523 | 1.289252 | 1.575685 | 0.001110165 | 0.001904398 |
| IGLL1 | 0.034382 | 0.378941 | 3.462227 | 7.19E-33 | 9.28E-32 |
| DMBT1 | 0.097885 | 1.323334 | 3.75695 | 0.00082353 | 0.001441922 |
| TIGIT | 0.360137 | 2.243279 | 2.638993 | 9.32E-128 | 1.25E-125 |
| CD6 | 0.699824 | 3.174751 | 2.181578 | 2.08E-129 | 3.21E-127 |
| SAMSN1 | 1.615204 | 4.499093 | 1.477918 | 1.40E-128 | 2.01E-126 |
| PLEK | 3.993901 | 14.53788 | 1.863946 | 1.05E-146 | 7.77E-144 |
| EPYC | 1.527821 | 3.21582 | 1.073711 | 7.12E-14 | 3.94E-13 |
| LYZ | 34.53586 | 160.7849 | 2.218965 | 1.10E-104 | 6.03E-103 |
| FCRL3 | 0.096509 | 0.807099 | 3.064015 | 5.03E-103 | 2.66E-101 |
| ITGB2 | 7.123773 | 22.16083 | 1.637298 | 1.10E-135 | 3.08E-133 |
| IL15 | 0.321385 | 0.693042 | 1.108642 | 1.54E-81 | 5.26E-80 |
| IL32 | 7.829915 | 21.32845 | 1.445711 | 9.46E-87 | 3.50E-85 |
| ICAM3 | 0.246246 | 1.133654 | 2.20281 | 2.47E-121 | 2.64E-119 |
| FAM129C | 0.074148 | 0.455248 | 2.618181 | 2.67E-61 | 6.03E-60 |
| TFEC | 0.553063 | 1.678037 | 1.601259 | 2.03E-109 | 1.30E-107 |
| AICDA | 0.011628 | 0.250456 | 4.428894 | 2.62E-43 | 4.19E-42 |
| GNGT2 | 0.506618 | 1.148519 | 1.180803 | 8.17E-132 | 1.59E-129 |
| KRT24 | 0.60056 | 0.266115 | -1.17426 | 0.000384602 | 0.000705028 |
| NCF1 | 0.392534 | 1.439147 | 1.874325 | 1.37E-128 | 1.99E-126 |
| HLA-DQB1 | 14.08285 | 45.89423 | 1.704374 | 1.22E-105 | 6.94E-104 |
| GIMAP4 | 8.115121 | 20.42345 | 1.331542 | 1.75E-124 | 2.10E-122 |
| RASAL3 | 1.106444 | 3.740479 | 1.757292 | 7.52E-132 | 1.49E-129 |
| FAM78A | 1.019118 | 2.805882 | 1.461133 | 2.51E-144 | 1.47E-141 |
| S100A9 | 205.4151 | 437.1756 | 1.089671 | 1.07E-31 | 1.34E-30 |
| MYO1F | 1.459071 | 3.870894 | 1.407617 | 9.90E-144 | 5.35E-141 |
| VAV1 | 1.736641 | 4.259851 | 1.294504 | 3.99E-110 | 2.65E-108 |
| SCML4 | 0.071414 | 0.407219 | 2.511536 | 2.66E-116 | 2.28E-114 |
| RPL3L | 0.473202 | 0.126707 | -1.90096 | 0.002028871 | 0.0033444 |
| HCST | 2.770032 | 9.656782 | 1.80164 | 5.47E-112 | 3.90E-110 |
| FYB1 | 2.291207 | 7.774309 | 1.762606 | 2.04E-123 | 2.37E-121 |
| KIF21B | 0.516993 | 1.175015 | 1.184462 | 9.46E-69 | 2.45E-67 |
| CELF2 | 2.135208 | 4.602043 | 1.107898 | 2.31E-79 | 7.38E-78 |
| KCNAB2 | 1.390122 | 3.224392 | 1.213815 | 1.60E-115 | 1.34E-113 |
| CXCL5 | 0.358493 | 1.348369 | 1.911201 | 2.00E-16 | 1.36E-15 |
| FABP7 | 9.619081 | 23.18034 | 1.268931 | 1.95E-09 | 7.06E-09 |
| TLR7 | 1.219307 | 2.801266 | 1.200018 | 4.62E-69 | 1.20E-67 |
| PRSS12 | 0.615391 | 1.375265 | 1.160134 | 1.42E-10 | 5.80E-10 |
| FXYD7 | 0.096378 | 0.22236 | 1.20613 | 1.90E-43 | 3.05E-42 |
| CST9 | 15.20943 | 4.59185 | -1.72782 | 4.65E-10 | 1.79E-09 |
| TSPAN32 | 0.170692 | 0.456907 | 1.420507 | 1.60E-74 | 4.63E-73 |
| AIM2 | 0.382965 | 2.168711 | 2.501551 | 9.99E-109 | 6.13E-107 |
| VCAM1 | 3.745961 | 10.82044 | 1.530351 | 1.37E-73 | 3.88E-72 |
| MS4A1 | 0.362419 | 4.362636 | 3.589469 | 1.62E-80 | 5.40E-79 |
| LSP1 | 7.779821 | 18.32917 | 1.236333 | 1.17E-100 | 5.92E-99 |
| ZBP1 | 0.386998 | 1.452838 | 1.908475 | 2.22E-90 | 8.95E-89 |
| FMNL1 | 2.443546 | 6.017114 | 1.300095 | 2.81E-133 | 6.38E-131 |
| TMEM82 | 0.158733 | 0.077651 | -1.03153 | 0.010050629 | 0.014926687 |
| SH2D2A | 1.105492 | 2.731366 | 1.304934 | 8.35E-80 | 2.72E-78 |
| EPSTI1 | 2.634503 | 7.077757 | 1.425761 | 3.04E-96 | 1.38E-94 |
| LAIR2 | 0.119313 | 0.567217 | 2.249148 | 7.87E-90 | 3.10E-88 |
| CD1A | 0.56847 | 1.288305 | 1.180317 | 9.89E-33 | 1.27E-31 |
| LILRA6 | 0.268373 | 0.611404 | 1.187887 | 4.31E-64 | 1.03E-62 |
| GNG8 | 0.241066 | 0.64977 | 1.430501 | 2.66E-45 | 4.50E-44 |
| SIRPG | 0.32783 | 2.181177 | 2.734087 | 7.45E-129 | 1.11E-126 |
| ZNF683 | 0.439931 | 2.168973 | 2.301663 | 7.79E-105 | 4.33E-103 |
| ACAP1 | 0.717267 | 2.607708 | 1.862201 | 2.16E-111 | 1.50E-109 |
| KLRB1 | 0.772325 | 2.895059 | 1.906312 | 6.95E-106 | 3.97E-104 |
| LAX1 | 0.338957 | 1.794399 | 2.404325 | 1.27E-93 | 5.50E-92 |
| SYNPO2L | 2.332612 | 0.776189 | -1.58747 | 4.11E-08 | 1.26E-07 |
| MILR1 | 1.525448 | 3.33005 | 1.126311 | 5.28E-109 | 3.30E-107 |
| CYTIP | 1.985883 | 6.135382 | 1.627372 | 2.74E-119 | 2.63E-117 |
| NEURL1 | 4.341683 | 2.104141 | -1.04502 | 7.57E-07 | 1.99E-06 |
| CEACAM4 | 0.070237 | 0.23775 | 1.759149 | 6.99E-84 | 2.49E-82 |
| CMKLR1 | 1.93092 | 4.355693 | 1.173614 | 6.81E-89 | 2.63E-87 |
| NLRC3 | 0.694715 | 1.645614 | 1.244133 | 5.08E-83 | 1.79E-81 |
| SHISAL2A | 0.186797 | 0.478513 | 1.357089 | 1.23E-66 | 3.12E-65 |
| OSM | 0.835909 | 1.952961 | 1.224245 | 3.61E-45 | 6.09E-44 |
| FCRL5 | 0.104228 | 0.719409 | 2.78707 | 3.01E-70 | 7.95E-69 |
| ADA2 | 6.733887 | 16.42818 | 1.286661 | 4.84E-97 | 2.21E-95 |
| FCRL1 | 0.045682 | 0.587427 | 3.684716 | 1.23E-73 | 3.50E-72 |
| CD19 | 0.161511 | 1.663684 | 3.364676 | 3.07E-81 | 1.04E-79 |
| ADGRE2 | 0.438149 | 0.970717 | 1.147628 | 4.19E-67 | 1.07E-65 |
| DNAJC12 | 30.89506 | 15.12761 | -1.03019 | 3.07E-16 | 2.06E-15 |
| HLA-DQA1 | 9.829626 | 35.02752 | 1.833281 | 2.85E-128 | 3.93E-126 |
| KLRC1 | 0.089825 | 0.271002 | 1.593116 | 2.79E-75 | 8.21E-74 |
| CD163 | 4.607153 | 13.59852 | 1.561503 | 8.80E-88 | 3.34E-86 |
| CADM2 | 0.429797 | 0.195555 | -1.13608 | 3.05E-12 | 1.48E-11 |
| IL1A | 0.105172 | 0.244684 | 1.21817 | 2.66E-19 | 2.20E-18 |
| GPR65 | 0.837371 | 2.14358 | 1.356083 | 5.02E-115 | 4.10E-113 |
| LY9 | 0.110588 | 0.640273 | 2.533493 | 1.11E-124 | 1.35E-122 |
| CEACAM21 | 0.508126 | 1.134848 | 1.159241 | 1.05E-82 | 3.66E-81 |
| HLA-DQA2 | 9.049241 | 31.60932 | 1.804481 | 6.94E-54 | 1.37E-52 |
| SLFN12L | 0.139616 | 0.581951 | 2.059435 | 1.72E-103 | 9.23E-102 |
| EEF1A2 | 76.19039 | 35.51471 | -1.10119 | 2.02E-14 | 1.18E-13 |
| ATRNL1 | 1.66729 | 0.594485 | -1.48779 | 7.16E-06 | 1.65E-05 |
| CCL4L2 | 1.876778 | 4.486244 | 1.25725 | 1.20E-63 | 2.86E-62 |
| ASB2 | 0.209612 | 0.574764 | 1.45525 | 4.15E-91 | 1.72E-89 |
| BATF2 | 1.690047 | 4.20576 | 1.315303 | 5.56E-43 | 8.84E-42 |
| SLAMF8 | 2.529914 | 9.297354 | 1.877732 | 1.87E-121 | 2.03E-119 |
| ZAP70 | 0.391927 | 2.151741 | 2.456849 | 1.85E-118 | 1.76E-116 |
| P2RY8 | 0.843876 | 2.809865 | 1.735399 | 9.29E-115 | 7.46E-113 |
| NLRP3 | 0.518622 | 1.285812 | 1.309925 | 1.20E-99 | 5.90E-98 |
| LILRB1 | 0.714825 | 2.178539 | 1.607699 | 1.69E-138 | 6.10E-136 |
| LOR | 0.05379 | 2.220533 | 5.367418 | 4.38E-18 | 3.36E-17 |
| C3AR1 | 5.313486 | 12.08741 | 1.185775 | 5.72E-104 | 3.09E-102 |
| CHRDL1 | 2.819713 | 6.150182 | 1.125081 | 1.31E-25 | 1.38E-24 |
| IRF1 | 5.73969 | 12.71951 | 1.147999 | 1.39E-87 | 5.24E-86 |
| TNFRSF17 | 0.547316 | 2.748315 | 2.328102 | 7.18E-61 | 1.60E-59 |
| BSN | 0.362768 | 0.176764 | -1.03723 | 1.32E-11 | 5.98E-11 |
| CHST2 | 1.480106 | 2.963886 | 1.001789 | 5.63E-59 | 1.20E-57 |
| SLC15A3 | 3.879962 | 8.438483 | 1.120941 | 2.53E-110 | 1.69E-108 |
| IL12B | 0.04179 | 0.22119 | 2.404044 | 8.14E-88 | 3.10E-86 |
| CYP2A6 | 38.44271 | 8.254789 | -2.21941 | 3.95E-07 | 1.07E-06 |
| GPR15 | 0.118753 | 0.438733 | 1.885374 | 1.11E-34 | 1.49E-33 |
| CD5 | 0.874616 | 4.603476 | 2.396003 | 1.94E-131 | 3.64E-129 |
| EOMES | 0.23968 | 1.359134 | 2.503509 | 1.93E-114 | 1.52E-112 |
| CARD17 | 0.171203 | 0.596161 | 1.799998 | 3.02E-61 | 6.82E-60 |
| BCL11B | 0.569724 | 1.398363 | 1.295403 | 6.75E-74 | 1.94E-72 |
| SLC1A2 | 2.5173 | 1.173112 | -1.10154 | 4.02E-12 | 1.93E-11 |
| ELOVL2 | 16.24208 | 7.350494 | -1.14382 | 3.27E-09 | 1.15E-08 |
| STX11 | 1.154619 | 2.733791 | 1.243485 | 2.57E-97 | 1.20E-95 |
| LILRA5 | 0.384655 | 1.09456 | 1.508713 | 2.98E-90 | 1.19E-88 |
| PIK3R5 | 0.800407 | 2.043902 | 1.352521 | 4.68E-131 | 8.65E-129 |
| RUFY4 | 0.148469 | 0.499516 | 1.750368 | 1.99E-55 | 4.07E-54 |
| AIF1 | 9.669088 | 24.64683 | 1.34995 | 5.08E-138 | 1.79E-135 |
| RBM24 | 5.206136 | 2.434314 | -1.0967 | 3.38E-05 | 7.14E-05 |
| SLC27A2 | 11.47964 | 5.036789 | -1.1885 | 3.16E-08 | 9.86E-08 |
| SELP | 1.513915 | 3.64206 | 1.266471 | 8.19E-35 | 1.11E-33 |
| IL15RA | 1.845356 | 4.524859 | 1.293974 | 6.36E-107 | 3.74E-105 |
| XCL2 | 0.345723 | 1.729091 | 2.322327 | 1.71E-100 | 8.54E-99 |
| SAMHD1 | 15.47446 | 31.17787 | 1.010633 | 1.12E-99 | 5.49E-98 |
| C10orf113 | 0.341566 | 0.16295 | -1.06773 | 0.000334237 | 0.000617293 |
| NLRP7 | 0.094456 | 0.230931 | 1.289743 | 3.72E-50 | 6.85E-49 |
| LMOD2 | 0.200152 | 0.034993 | -2.51597 | 0.002254011 | 0.003686545 |
| APOL3 | 4.213691 | 9.523727 | 1.176442 | 1.03E-82 | 3.60E-81 |
| AGXT | 0.098033 | 0.233437 | 1.251696 | 5.85E-08 | 1.76E-07 |
| GIMAP6 | 3.614413 | 7.622077 | 1.076423 | 2.50E-80 | 8.23E-79 |
| SLC5A7 | 0.203929 | 0.051424 | -1.98756 | 4.90E-05 | 0.00010133 |
| WAS | 2.409244 | 7.629757 | 1.663057 | 4.00E-155 | 1.40E-151 |
| SELE | 1.415946 | 3.109821 | 1.135065 | 3.69E-39 | 5.42E-38 |
| CFP | 0.206349 | 0.586777 | 1.507725 | 1.03E-61 | 2.35E-60 |
| CXorf21 | 0.977818 | 2.719981 | 1.475959 | 2.51E-114 | 1.94E-112 |
| FAM92B | 0.121356 | 0.294905 | 1.281006 | 1.44E-27 | 1.60E-26 |
| CD300C | 0.789494 | 1.918509 | 1.280985 | 3.06E-101 | 1.57E-99 |
| PATL2 | 0.157405 | 0.490245 | 1.639025 | 1.75E-82 | 6.07E-81 |
| ALOX5AP | 7.184716 | 15.07649 | 1.069297 | 2.61E-79 | 8.34E-78 |
| CNR2 | 0.031967 | 0.247811 | 2.954585 | 4.65E-78 | 1.45E-76 |
| SLAMF1 | 0.25612 | 1.482803 | 2.533435 | 2.73E-130 | 4.57E-128 |
| CD40LG | 0.293367 | 1.492863 | 2.347303 | 5.62E-110 | 3.69E-108 |
| KCNJ10 | 0.109406 | 0.422396 | 1.948904 | 1.75E-53 | 3.42E-52 |
| LILRB5 | 0.254089 | 0.736117 | 1.534602 | 2.51E-68 | 6.49E-67 |
| CD74 | 328.7019 | 961.0342 | 1.547808 | 4.32E-135 | 1.15E-132 |
| AOAH | 1.239953 | 4.299499 | 1.793883 | 3.39E-136 | 1.01E-133 |
| FCRL6 | 0.22403 | 0.780639 | 1.800963 | 2.49E-95 | 1.11E-93 |
| SELPLG | 6.4439 | 17.48208 | 1.439871 | 2.38E-140 | 1.11E-137 |
| TNFRSF8 | 0.195045 | 0.63337 | 1.699239 | 1.57E-113 | 1.17E-111 |
| IL2RB | 2.648928 | 7.709487 | 1.541226 | 1.15E-110 | 7.84E-109 |
| HLA-F | 9.864591 | 23.87071 | 1.27491 | 4.63E-77 | 1.42E-75 |
| CD68 | 0.409773 | 0.917521 | 1.162915 | 1.18E-63 | 2.80E-62 |
| PTGDS | 3.919429 | 18.25809 | 2.219821 | 2.28E-64 | 5.48E-63 |
| COL4A4 | 0.240789 | 0.516146 | 1.100006 | 3.70E-46 | 6.35E-45 |
| 1-Sep | 1.488835 | 3.615399 | 1.279971 | 9.53E-65 | 2.30E-63 |
| CD209 | 0.452274 | 1.381092 | 1.610541 | 6.81E-71 | 1.82E-69 |
| FCGR1A | 1.167323 | 2.745781 | 1.234013 | 3.78E-86 | 1.39E-84 |
| IL1B | 0.991098 | 2.232335 | 1.171454 | 2.00E-44 | 3.32E-43 |
| CXCR6 | 0.483699 | 2.092279 | 2.112893 | 7.87E-121 | 8.19E-119 |
| SEL1L3 | 3.164805 | 6.981705 | 1.141463 | 3.63E-51 | 6.84E-50 |
| CASP5 | 0.059094 | 0.210542 | 1.833028 | 1.65E-62 | 3.83E-61 |
| CD200R1 | 0.224573 | 0.683567 | 1.6059 | 3.13E-101 | 1.59E-99 |
| PDCD1LG2 | 1.015692 | 2.93933 | 1.533025 | 2.74E-106 | 1.58E-104 |
| GZMB | 0.66685 | 5.607622 | 3.071955 | 8.21E-108 | 4.95E-106 |
| CXCL9 | 9.236684 | 76.20409 | 3.044422 | 3.66E-100 | 1.82E-98 |
| CD40 | 3.508882 | 8.360554 | 1.252587 | 1.92E-112 | 1.39E-110 |
| LY96 | 6.264998 | 13.71803 | 1.130688 | 2.86E-110 | 1.90E-108 |
| JAK3 | 1.017869 | 3.670886 | 1.850577 | 1.94E-120 | 1.96E-118 |
| CASP1 | 3.315112 | 7.201587 | 1.119257 | 2.36E-94 | 1.03E-92 |
| GBP4 | 4.084759 | 15.93232 | 1.963634 | 1.82E-96 | 8.29E-95 |
| XCL1 | 0.229746 | 0.782118 | 1.767347 | 1.31E-90 | 5.36E-89 |
| LILRB3 | 0.247963 | 0.642183 | 1.372861 | 1.41E-111 | 9.88E-110 |
| BANK1 | 0.205965 | 1.188642 | 2.528845 | 1.91E-76 | 5.79E-75 |
| CSF2RB | 1.557809 | 5.784245 | 1.89261 | 5.95E-119 | 5.68E-117 |
| CTLA4 | 0.364132 | 2.02906 | 2.478278 | 7.82E-114 | 6.01E-112 |
| SIGLEC14 | 0.831701 | 2.232007 | 1.424205 | 4.89E-73 | 1.37E-71 |
| SLC1A3 | 1.90616 | 3.943512 | 1.048812 | 7.99E-70 | 2.10E-68 |
| MATK | 0.722967 | 1.596757 | 1.143144 | 3.63E-55 | 7.35E-54 |
| SLAMF7 | 1.423813 | 7.488385 | 2.394894 | 1.74E-117 | 1.59E-115 |
| HVCN1 | 0.978872 | 2.252031 | 1.202035 | 9.92E-111 | 6.80E-109 |
| TDRD12 | 0.474346 | 0.975005 | 1.039471 | 0.001135753 | 0.001945679 |
| CD53 | 9.824276 | 33.35276 | 1.763383 | 6.10E-161 | 8.58E-157 |
| CD1D | 0.543375 | 1.34755 | 1.310317 | 6.06E-87 | 2.25E-85 |
| SERPINC1 | 0.418066 | 0.145219 | -1.5255 | 0.004240762 | 0.006667444 |
| LCP1 | 23.19748 | 48.45152 | 1.062574 | 1.99E-73 | 5.63E-72 |
| ADGRG5 | 0.204822 | 0.704753 | 1.782746 | 1.17E-82 | 4.07E-81 |
| CXCL10 | 17.93718 | 83.87767 | 2.225334 | 1.42E-87 | 5.34E-86 |
| CCL19 | 7.886755 | 50.07656 | 2.666632 | 7.83E-70 | 2.06E-68 |
| CARMIL2 | 0.452541 | 1.002959 | 1.148143 | 2.77E-52 | 5.30E-51 |
| STH | 0.28869 | 0.138262 | -1.06211 | 1.94E-08 | 6.22E-08 |
| HAPLN3 | 5.428166 | 11.19451 | 1.044255 | 3.66E-71 | 9.88E-70 |
| PAH | 0.463028 | 0.188141 | -1.29928 | 0.000123958 | 0.000242244 |
| BATF3 | 0.439747 | 0.896026 | 1.026867 | 1.44E-72 | 3.98E-71 |
| TREML1 | 0.22553 | 0.474348 | 1.072625 | 1.33E-60 | 2.93E-59 |
| IVL | 0.380492 | 0.858277 | 1.173578 | 5.14E-06 | 1.21E-05 |
| CTSW | 1.111605 | 5.364228 | 2.270727 | 1.56E-111 | 1.08E-109 |
| RIPOR2 | 0.61958 | 1.967986 | 1.667358 | 3.45E-78 | 1.08E-76 |
| VSTM2A | 6.415709 | 2.833143 | -1.17921 | 0.023760183 | 0.033199516 |
| S100A7 | 68.97529 | 169.6142 | 1.298105 | 4.44E-11 | 1.91E-10 |
| TAP1 | 19.20664 | 47.48139 | 1.305757 | 3.47E-61 | 7.82E-60 |
| APLP1 | 5.976861 | 2.758265 | -1.11563 | 0.010626422 | 0.01571373 |
| RGS1 | 9.797021 | 21.99749 | 1.166924 | 8.33E-58 | 1.76E-56 |
| PILRA | 2.472676 | 5.421897 | 1.132724 | 3.73E-103 | 1.99E-101 |
| TMPRSS6 | 3.147708 | 1.518061 | -1.05207 | 3.97E-09 | 1.38E-08 |
| FPR2 | 0.061239 | 0.187096 | 1.611244 | 1.28E-66 | 3.23E-65 |
| LRRC25 | 1.666799 | 3.907518 | 1.229172 | 3.54E-120 | 3.53E-118 |
| IL18BP | 1.939194 | 4.985168 | 1.362185 | 4.84E-116 | 4.12E-114 |
| MAL | 0.749726 | 1.533159 | 1.032072 | 1.61E-79 | 5.17E-78 |
| LAIR1 | 2.017894 | 5.293509 | 1.391374 | 2.44E-140 | 1.11E-137 |
| PTPN22 | 0.61686 | 1.932149 | 1.64719 | 4.89E-115 | 4.04E-113 |
| HEPACAM2 | 2.518329 | 1.128426 | -1.15816 | 8.61E-06 | 1.97E-05 |
| SULT1B1 | 0.10157 | 0.323913 | 1.673136 | 2.61E-14 | 1.50E-13 |
| SAMD9L | 5.074145 | 10.67234 | 1.072639 | 4.22E-54 | 8.35E-53 |
| NXPH1 | 3.386996 | 1.292288 | -1.39008 | 0.008731277 | 0.013094586 |
| PARP15 | 0.205554 | 0.957796 | 2.220198 | 7.46E-111 | 5.13E-109 |
| GLT1D1 | 0.070016 | 0.154264 | 1.139649 | 1.46E-60 | 3.22E-59 |
| GRAP2 | 0.308058 | 1.00692 | 1.708676 | 5.45E-104 | 2.96E-102 |
| CCL22 | 1.662865 | 3.870165 | 1.218724 | 9.81E-70 | 2.57E-68 |
| CORO1A | 4.944348 | 17.8596 | 1.852848 | 1.63E-133 | 3.82E-131 |
| IL6 | 0.819536 | 2.113633 | 1.366846 | 1.91E-36 | 2.69E-35 |
| VNN1 | 0.479029 | 1.378981 | 1.525417 | 9.88E-73 | 2.76E-71 |
| MARCO | 1.058617 | 5.488448 | 2.374218 | 4.14E-44 | 6.80E-43 |
| TRAF3IP3 | 0.579788 | 1.94922 | 1.749301 | 3.59E-120 | 3.55E-118 |
| HK3 | 0.637431 | 2.316784 | 1.861783 | 3.91E-79 | 1.24E-77 |
| CD247 | 0.536309 | 2.654156 | 2.307116 | 9.21E-131 | 1.66E-128 |
| CSF1R | 7.197047 | 17.40672 | 1.274167 | 2.19E-109 | 1.40E-107 |
| SLAMF6 | 0.645774 | 4.086931 | 2.661916 | 1.51E-130 | 2.62E-128 |
| GPR18 | 0.34754 | 1.079952 | 1.635717 | 1.60E-95 | 7.16E-94 |
| UBASH3A | 0.235284 | 1.224017 | 2.379147 | 1.99E-127 | 2.64E-125 |
| CCR8 | 0.340568 | 0.946417 | 1.474532 | 6.75E-71 | 1.81E-69 |
| BRINP2 | 2.919475 | 1.242917 | -1.23198 | 2.41E-06 | 5.92E-06 |
| TRIM22 | 6.31679 | 13.98463 | 1.146578 | 2.99E-80 | 9.85E-79 |
| CLECL1 | 0.213823 | 0.68014 | 1.669415 | 1.74E-98 | 8.29E-97 |
| LGALS2 | 1.134108 | 4.48398 | 1.983221 | 3.97E-88 | 1.52E-86 |
| IRF4 | 0.442683 | 2.160232 | 2.286841 | 1.47E-97 | 6.91E-96 |
| PIGR | 9.286058 | 20.77531 | 1.161732 | 1.29E-11 | 5.84E-11 |
| FCGR3A | 16.83488 | 39.72819 | 1.23871 | 9.02E-77 | 2.75E-75 |
| BLK | 0.082702 | 0.687087 | 3.054494 | 3.90E-73 | 1.10E-71 |
| ASCL1 | 4.166914 | 1.121551 | -1.89348 | 0.019652101 | 0.027830243 |
| CTSC | 5.561744 | 12.65808 | 1.18645 | 5.16E-84 | 1.85E-82 |
| TNFSF14 | 0.178472 | 0.6619 | 1.890917 | 2.92E-109 | 1.86E-107 |
| GZMM | 0.603185 | 3.347119 | 2.472248 | 8.20E-105 | 4.54E-103 |
| KRT2 | 0.056481 | 0.177211 | 1.649631 | 9.34E-18 | 7.01E-17 |
| C1orf162 | 3.025703 | 7.585717 | 1.326015 | 1.21E-133 | 2.88E-131 |
| SPNS3 | 0.194038 | 0.446319 | 1.20174 | 5.20E-55 | 1.05E-53 |
| ITGAD | 0.105099 | 0.293175 | 1.480018 | 5.61E-51 | 1.05E-49 |
| CCR1 | 2.985659 | 7.874838 | 1.399201 | 8.63E-95 | 3.81E-93 |
| PDE6G | 0.325629 | 0.916473 | 1.492864 | 8.79E-100 | 4.33E-98 |
| ICAM1 | 9.285727 | 19.36187 | 1.060132 | 2.19E-58 | 4.66E-57 |
| CLEC4A | 1.37189 | 3.558918 | 1.375274 | 8.00E-130 | 1.28E-127 |
| DERL3 | 2.990785 | 7.530158 | 1.332156 | 1.47E-47 | 2.57E-46 |
| CSMD3 | 0.210589 | 0.04279 | -2.29907 | 2.00E-05 | 4.35E-05 |
| CASS4 | 0.288288 | 0.671926 | 1.220794 | 1.86E-102 | 9.72E-101 |
| IL10 | 0.228828 | 0.601833 | 1.395098 | 1.62E-89 | 6.36E-88 |
| GLYAT | 0.096429 | 0.198698 | 1.043036 | 2.29E-08 | 7.26E-08 |
| C11orf16 | 0.144595 | 0.292705 | 1.017427 | 3.10E-07 | 8.55E-07 |
| CLEC7A | 2.125875 | 4.641335 | 1.126483 | 5.76E-92 | 2.39E-90 |
| IFI44L | 4.686194 | 9.71203 | 1.051356 | 8.82E-39 | 1.29E-37 |
| NOVA1 | 3.872559 | 1.613009 | -1.26353 | 6.84E-05 | 0.000138888 |
| PYHIN1 | 0.208015 | 1.211585 | 2.542133 | 6.82E-130 | 1.12E-127 |
| C5AR1 | 3.289463 | 6.631119 | 1.0114 | 7.84E-71 | 2.09E-69 |
| CGA | 18.56825 | 3.694325 | -2.32946 | 0.001361412 | 0.002306113 |
| TRIM55 | 0.197697 | 0.539733 | 1.448956 | 1.78E-12 | 8.82E-12 |
| PHF21B | 0.503758 | 0.189628 | -1.40956 | 2.15E-07 | 6.04E-07 |
| GMFG | 5.407372 | 14.84332 | 1.456814 | 2.53E-135 | 6.84E-133 |
| APOE | 77.27552 | 188.0017 | 1.282662 | 7.47E-63 | 1.74E-61 |
| CLEC4G | 0.05737 | 0.156504 | 1.447824 | 1.08E-33 | 1.42E-32 |
| AQP9 | 0.690185 | 1.955602 | 1.502559 | 3.67E-27 | 4.02E-26 |
| IL12RB2 | 0.460335 | 0.93434 | 1.021262 | 4.44E-45 | 7.47E-44 |
| KCNC1 | 0.329987 | 0.057071 | -2.53157 | 1.89E-05 | 4.13E-05 |
| PNMA5 | 0.112584 | 0.274354 | 1.285033 | 1.34E-15 | 8.58E-15 |
| MYO1G | 0.666782 | 1.910332 | 1.518536 | 7.94E-110 | 5.19E-108 |
| PSORS1C2 | 0.454477 | 1.490809 | 1.713816 | 0.005012055 | 0.007791169 |
| DPT | 13.89073 | 29.6169 | 1.092299 | 1.18E-39 | 1.76E-38 |
| IKZF3 | 1.101804 | 3.397256 | 1.624502 | 3.86E-81 | 1.30E-79 |
| CCDC88B | 0.928483 | 2.364873 | 1.348816 | 4.67E-71 | 1.25E-69 |
| TNFRSF4 | 1.403285 | 3.306892 | 1.236667 | 1.91E-60 | 4.20E-59 |
| CELF3 | 0.347883 | 0.102594 | -1.76166 | 9.26E-06 | 2.11E-05 |
| NCF2 | 3.544507 | 8.780669 | 1.308746 | 6.16E-105 | 3.45E-103 |
| GIMAP7 | 5.36851 | 14.7958 | 1.462594 | 1.82E-94 | 8.00E-93 |
| BIRC3 | 2.573172 | 8.057184 | 1.646728 | 4.25E-82 | 1.46E-80 |
| KIF19 | 0.140526 | 0.323102 | 1.201154 | 4.45E-41 | 6.79E-40 |
| EVI2A | 2.88782 | 7.141626 | 1.306272 | 2.21E-116 | 1.92E-114 |
| MMP1 | 10.74983 | 26.23706 | 1.287292 | 1.32E-10 | 5.40E-10 |
| ACHE | 0.384706 | 0.860718 | 1.161787 | 2.48E-31 | 3.08E-30 |
| NELL1 | 0.931285 | 0.198018 | -2.23359 | 0.01143069 | 0.016826934 |
| EBI3 | 1.020983 | 3.338253 | 1.709135 | 1.57E-110 | 1.07E-108 |
| CD79A | 2.144507 | 17.05585 | 2.991549 | 9.08E-79 | 2.87E-77 |
| GZMA | 2.483788 | 13.48148 | 2.440365 | 6.67E-130 | 1.10E-127 |
| APOBEC3G | 1.416797 | 4.048605 | 1.514792 | 9.54E-109 | 5.88E-107 |
| MIXL1 | 0.158979 | 0.332112 | 1.062833 | 1.99E-40 | 3.01E-39 |
| SEZ6L | 2.443218 | 0.813392 | -1.58676 | 0.007676981 | 0.011616332 |
| RTBDN | 1.270574 | 0.496896 | -1.35447 | 0.008220311 | 0.012386444 |
| FCGR1B | 0.17452 | 0.402142 | 1.204315 | 2.00E-81 | 6.80E-80 |
| CD300LG | 0.399708 | 0.808159 | 1.015694 | 1.03E-15 | 6.67E-15 |
| RASGRP2 | 0.38739 | 1.413325 | 1.867234 | 1.09E-80 | 3.66E-79 |
| CRTAM | 0.217137 | 1.033831 | 2.251324 | 1.58E-139 | 6.73E-137 |
| DOCK2 | 0.981581 | 3.092763 | 1.655718 | 6.45E-134 | 1.56E-131 |
| SRGN | 27.39092 | 80.60596 | 1.557189 | 3.15E-137 | 1.05E-134 |
| S1PR4 | 0.466664 | 2.494972 | 2.418569 | 9.88E-121 | 1.02E-118 |
| LCP2 | 2.087372 | 6.086711 | 1.543975 | 1.56E-151 | 2.20E-148 |
| TSHR | 0.071266 | 0.154011 | 1.111755 | 5.35E-48 | 9.46E-47 |
| LTA | 0.201562 | 1.14532 | 2.506454 | 4.69E-117 | 4.15E-115 |
| LGALS9 | 6.809969 | 14.66654 | 1.106808 | 1.02E-87 | 3.85E-86 |
| PRF1 | 0.846004 | 4.485447 | 2.406516 | 1.95E-129 | 3.05E-127 |
| ACY3 | 0.765605 | 2.753963 | 1.846837 | 1.63E-38 | 2.37E-37 |
| INHA | 2.763571 | 0.933848 | -1.56527 | 7.64E-05 | 0.000153853 |
| FATE1 | 0.172465 | 0.421235 | 1.288324 | 7.78E-13 | 3.98E-12 |
| PARVG | 0.821954 | 2.27976 | 1.471753 | 1.67E-138 | 6.10E-136 |
| CYBB | 7.108505 | 22.80077 | 1.681465 | 7.73E-112 | 5.46E-110 |
| P2RX5 | 0.215259 | 0.598933 | 1.476323 | 5.84E-53 | 1.13E-51 |
| CNMD | 1.049681 | 0.163687 | -2.68094 | 0.021415437 | 0.030133016 |
| NRCAM | 2.638074 | 1.181103 | -1.15935 | 3.81E-05 | 8.01E-05 |
| GIMAP8 | 2.209246 | 4.473376 | 1.01781 | 5.28E-76 | 1.58E-74 |
| RNASE6 | 7.772534 | 18.52142 | 1.252738 | 1.83E-115 | 1.53E-113 |
| C1S | 34.56811 | 77.92432 | 1.172632 | 4.34E-78 | 1.36E-76 |
| SAMD3 | 0.083205 | 0.366503 | 2.139077 | 1.32E-122 | 1.49E-120 |
| NLRC5 | 1.988822 | 5.114674 | 1.362728 | 7.68E-91 | 3.16E-89 |
| P2RX1 | 0.198108 | 0.63033 | 1.669822 | 9.23E-65 | 2.23E-63 |
| GFI1 | 0.250294 | 0.90109 | 1.848046 | 1.89E-108 | 1.15E-106 |
| ITGB7 | 0.429987 | 1.333582 | 1.632942 | 5.56E-88 | 2.12E-86 |
| OR52N4 | 0.065052 | 0.145548 | 1.161818 | 2.44E-41 | 3.75E-40 |
| SIGLEC7 | 0.491138 | 1.233675 | 1.328761 | 2.49E-108 | 1.52E-106 |
| CD3E | 2.020511 | 12.02403 | 2.573128 | 5.30E-137 | 1.69E-134 |
| CCR5 | 1.367914 | 5.875342 | 2.102696 | 7.54E-148 | 7.56E-145 |
| ZC3H12D | 0.155402 | 0.564148 | 1.860072 | 4.04E-114 | 3.12E-112 |
| BTK | 1.172209 | 3.22393 | 1.45959 | 6.31E-138 | 2.16E-135 |
| CH25H | 1.008136 | 2.26775 | 1.169572 | 2.16E-44 | 3.58E-43 |
| HCLS1 | 4.464796 | 13.28875 | 1.573539 | 1.57E-153 | 3.68E-150 |
| CR2 | 0.275036 | 2.02558 | 2.880645 | 5.35E-42 | 8.37E-41 |
| TCL1A | 0.108982 | 1.758924 | 4.012537 | 2.39E-82 | 8.24E-81 |
| APOBEC3D | 1.179272 | 2.599746 | 1.140474 | 6.09E-65 | 1.48E-63 |
| PIM2 | 6.785205 | 16.83268 | 1.3108 | 9.95E-60 | 2.16E-58 |
| AC119396.1 | 0.181487 | 0.480019 | 1.403228 | 1.03E-32 | 1.32E-31 |
| XCR1 | 0.105497 | 0.449337 | 2.090593 | 1.95E-90 | 7.91E-89 |
| P2RY13 | 0.997362 | 2.461426 | 1.303306 | 3.79E-72 | 1.04E-70 |
| LIPG | 0.312393 | 0.631881 | 1.016293 | 2.47E-33 | 3.23E-32 |
| HLA-DPA1 | 26.49289 | 79.34248 | 1.582488 | 7.06E-129 | 1.07E-126 |
| LILRA4 | 0.342767 | 1.228187 | 1.84123 | 7.48E-75 | 2.17E-73 |
| GPR174 | 0.239877 | 1.432038 | 2.577706 | 2.88E-97 | 1.34E-95 |
| CPLX2 | 2.409732 | 0.315101 | -2.93499 | 0.016559305 | 0.023737481 |
| LPXN | 5.121795 | 11.51684 | 1.169023 | 2.50E-128 | 3.48E-126 |
| GPR25 | 0.040279 | 0.185242 | 2.201324 | 5.93E-49 | 1.06E-47 |
| PXDNL | 2.745696 | 1.136145 | -1.27302 | 0.012691584 | 0.018560517 |
| HCK | 3.721141 | 9.170637 | 1.301277 | 2.28E-119 | 2.21E-117 |
| IL22RA2 | 0.180883 | 0.458705 | 1.342509 | 1.60E-37 | 2.29E-36 |
| VSIR | 3.774496 | 8.402047 | 1.154457 | 3.13E-99 | 1.52E-97 |
| PIK3CG | 0.551005 | 1.57042 | 1.511013 | 7.56E-80 | 2.46E-78 |
| SLA2 | 0.415818 | 2.07807 | 2.321219 | 2.52E-135 | 6.84E-133 |
| PLD4 | 0.771197 | 1.689777 | 1.131661 | 6.19E-39 | 9.03E-38 |
| PYY | 1.418802 | 0.658877 | -1.10659 | 1.01E-08 | 3.34E-08 |
| BIRC7 | 0.155263 | 0.321317 | 1.049279 | 1.31E-22 | 1.25E-21 |
| TNFRSF13B | 0.05906 | 0.366223 | 2.632479 | 1.27E-73 | 3.60E-72 |
| FCRLA | 0.166479 | 1.206085 | 2.856922 | 6.07E-87 | 2.25E-85 |
| SNAI3 | 0.470453 | 1.06852 | 1.18349 | 9.54E-79 | 3.00E-77 |
| G0S2 | 9.983532 | 22.06152 | 1.14391 | 3.07E-32 | 3.89E-31 |
| IKZF1 | 0.874813 | 3.287529 | 1.909957 | 2.23E-136 | 6.98E-134 |
| TBX21 | 0.165547 | 0.947821 | 2.517374 | 7.21E-130 | 1.16E-127 |
| SIRPB2 | 0.443028 | 1.06945 | 1.271398 | 1.10E-98 | 5.30E-97 |
| IL9R | 0.094164 | 0.299627 | 1.669917 | 2.24E-80 | 7.41E-79 |
| MMP9 | 27.92517 | 103.6546 | 1.892147 | 2.43E-31 | 3.02E-30 |
| SYP | 2.268337 | 0.950517 | -1.25485 | 0.020947906 | 0.029525432 |
| FGL2 | 4.359817 | 13.00818 | 1.57708 | 3.93E-112 | 2.82E-110 |
| C3 | 36.39387 | 95.23623 | 1.387815 | 6.74E-90 | 2.66E-88 |
| EMILIN2 | 1.567767 | 3.621626 | 1.207926 | 2.39E-94 | 1.04E-92 |
| IL18R1 | 0.454398 | 1.018472 | 1.164378 | 6.65E-75 | 1.93E-73 |
| CD79B | 1.142108 | 5.54426 | 2.279296 | 2.25E-80 | 7.44E-79 |
| SYT13 | 19.30939 | 7.425875 | -1.37867 | 1.14E-07 | 3.30E-07 |
| LILRA1 | 0.1827 | 0.373 | 1.029702 | 1.23E-65 | 3.03E-64 |
| DCSTAMP | 0.186186 | 0.568084 | 1.609356 | 1.11E-39 | 1.65E-38 |
| S100A7A | 1.491685 | 3.051505 | 1.032578 | 2.73E-08 | 8.57E-08 |
| WDR72 | 1.023953 | 0.359769 | -1.50901 | 0.001462776 | 0.002465035 |
| CDX1 | 0.160929 | 0.364943 | 1.181246 | 2.31E-40 | 3.49E-39 |
| HLA-DRB1 | 155.2776 | 476.4428 | 1.617454 | 2.63E-133 | 6.05E-131 |
| CD37 | 2.51544 | 9.05279 | 1.847552 | 1.20E-151 | 2.18E-148 |
| CARD11 | 0.807088 | 2.866293 | 1.828389 | 4.61E-136 | 1.35E-133 |
| COL2A1 | 17.24805 | 7.483396 | -1.20467 | 1.59E-09 | 5.79E-09 |
| IL2RA | 0.617924 | 2.192757 | 1.827245 | 1.27E-103 | 6.83E-102 |
| CCL8 | 1.836368 | 5.874714 | 1.677663 | 2.27E-59 | 4.88E-58 |
| CCR4 | 0.523299 | 2.247439 | 2.102575 | 1.22E-106 | 7.14E-105 |
| GPA33 | 0.075951 | 0.168977 | 1.15369 | 4.45E-68 | 1.15E-66 |
| SIGLEC10 | 1.23276 | 3.389381 | 1.45913 | 1.18E-113 | 8.93E-112 |
| MEOX1 | 1.317589 | 2.813146 | 1.094284 | 3.90E-29 | 4.55E-28 |
| LGI2 | 0.556019 | 1.156438 | 1.056481 | 1.37E-50 | 2.56E-49 |
| CLIC2 | 2.869073 | 6.186654 | 1.108575 | 7.86E-91 | 3.22E-89 |
| TMC8 | 1.195404 | 4.4766 | 1.904906 | 1.71E-134 | 4.36E-132 |
| SLC26A9 | 0.146692 | 0.345902 | 1.237572 | 2.04E-20 | 1.77E-19 |
| RGS18 | 0.337877 | 0.956981 | 1.501991 | 4.13E-101 | 2.09E-99 |
| CLDN16 | 0.06403 | 0.187972 | 1.553693 | 5.12E-18 | 3.92E-17 |
| GBP1 | 7.781366 | 27.10134 | 1.800269 | 2.35E-94 | 1.03E-92 |
| THEMIS2 | 2.693268 | 6.793014 | 1.334694 | 4.98E-115 | 4.09E-113 |
| XPNPEP2 | 0.195761 | 0.480134 | 1.294342 | 6.35E-51 | 1.19E-49 |
| CCL11 | 0.794562 | 2.012269 | 1.340591 | 2.26E-49 | 4.07E-48 |
| CR1 | 0.059578 | 0.255397 | 2.099897 | 1.22E-72 | 3.38E-71 |
| CCL20 | 0.698597 | 1.575885 | 1.17363 | 8.83E-34 | 1.16E-32 |
| RUBCNL | 0.698849 | 1.451323 | 1.054316 | 8.11E-67 | 2.06E-65 |
| LTB | 2.017821 | 13.38016 | 2.729225 | 9.39E-99 | 4.54E-97 |
| GPR84 | 0.565085 | 1.512049 | 1.419964 | 1.69E-69 | 4.42E-68 |
| CLEC17A | 0.090124 | 0.440683 | 2.289756 | 4.61E-62 | 1.06E-60 |
| GIMAP1 | 0.926533 | 2.273494 | 1.294997 | 4.03E-92 | 1.68E-90 |
| TMEM273 | 1.047735 | 2.387127 | 1.188002 | 2.92E-92 | 1.22E-90 |
| TMIGD2 | 0.086903 | 0.408492 | 2.232826 | 5.19E-87 | 1.94E-85 |
| IFNG | 0.071063 | 0.700176 | 3.300556 | 1.44E-95 | 6.50E-94 |
| GAPT | 0.9577 | 1.97735 | 1.045923 | 2.51E-47 | 4.36E-46 |
| GPR171 | 0.33099 | 1.778194 | 2.425552 | 1.39E-114 | 1.11E-112 |
| CYP2A7 | 12.71347 | 5.245814 | -1.27712 | 0.00019197 | 0.000365696 |
| FPR1 | 1.424637 | 3.456329 | 1.278646 | 1.41E-79 | 4.57E-78 |
| PSTPIP1 | 0.485968 | 2.036759 | 2.067342 | 1.30E-139 | 5.72E-137 |
| LYN | 3.842287 | 8.215278 | 1.096344 | 2.91E-92 | 1.22E-90 |
| KLRG1 | 0.420376 | 1.005759 | 1.258531 | 4.90E-66 | 1.22E-64 |
| SERPINA6 | 34.95574 | 17.00567 | -1.03951 | 0.000459399 | 0.000833981 |
| DOK3 | 1.353574 | 2.904731 | 1.101631 | 2.59E-106 | 1.50E-104 |
| MEI1 | 0.276672 | 0.944399 | 1.771219 | 1.28E-105 | 7.23E-104 |
| HLA-DRA | 285.8781 | 895.1658 | 1.646755 | 4.05E-145 | 2.59E-142 |
| CIITA | 0.982007 | 3.271968 | 1.736353 | 5.69E-118 | 5.26E-116 |
| GBP2 | 12.08371 | 24.16845 | 1.000061 | 1.18E-65 | 2.92E-64 |
| CD244 | 0.138911 | 0.504846 | 1.861688 | 1.43E-113 | 1.07E-111 |
| CXCR2 | 0.097425 | 0.213826 | 1.134077 | 8.35E-42 | 1.30E-40 |
| MMP12 | 2.05667 | 6.012072 | 1.547552 | 6.54E-19 | 5.29E-18 |
| CD28 | 0.427564 | 1.510206 | 1.820533 | 9.78E-110 | 6.36E-108 |
| PTPRC | 2.750215 | 11.96008 | 2.120611 | 8.10E-131 | 1.48E-128 |
| NSG2 | 2.723994 | 0.326545 | -3.06037 | 0.000963824 | 0.001668975 |
| TRAT1 | 0.178638 | 1.310553 | 2.875065 | 1.21E-120 | 1.24E-118 |
| CCL18 | 2.098606 | 10.43346 | 2.313714 | 5.15E-52 | 9.79E-51 |
| CD4 | 7.508557 | 22.507 | 1.583766 | 2.97E-154 | 8.35E-151 |
| TNFAIP8L2 | 2.373394 | 6.20797 | 1.38717 | 5.92E-132 | 1.19E-129 |
| CACNB2 | 1.008309 | 0.446803 | -1.17423 | 0.016748756 | 0.023991923 |
| CD300E | 0.348594 | 0.92213 | 1.403421 | 2.38E-61 | 5.38E-60 |
| FOLR2 | 3.872181 | 10.53635 | 1.444157 | 1.89E-69 | 4.93E-68 |
| RGS13 | 0.078949 | 0.163357 | 1.049026 | 1.80E-29 | 2.12E-28 |
| ABCD2 | 0.128532 | 0.370276 | 1.526471 | 8.57E-77 | 2.62E-75 |
| CD33 | 0.373785 | 0.871544 | 1.221363 | 1.96E-114 | 1.54E-112 |
| IFI30 | 0.276952 | 0.608498 | 1.135618 | 6.42E-80 | 2.10E-78 |
| LST1 | 2.222716 | 5.978787 | 1.427529 | 1.04E-132 | 2.18E-130 |
| CD300A | 2.440412 | 5.251799 | 1.105687 | 1.05E-101 | 5.42E-100 |
| GPR183 | 3.399494 | 11.26382 | 1.728304 | 2.26E-113 | 1.68E-111 |
| ABI3 | 2.846363 | 6.200647 | 1.123299 | 6.72E-117 | 5.90E-115 |
| TRAF1 | 1.644179 | 3.839012 | 1.223368 | 6.74E-85 | 2.44E-83 |
| CD3G | 0.390554 | 2.207943 | 2.499108 | 2.24E-128 | 3.18E-126 |
| VPREB3 | 0.590733 | 2.944978 | 2.317678 | 6.82E-44 | 1.11E-42 |
| KLRD1 | 0.091071 | 0.314607 | 1.788484 | 3.95E-97 | 1.81E-95 |
| FCER1G | 21.98858 | 52.98466 | 1.26882 | 1.02E-122 | 1.16E-120 |
| PI3 | 10.92156 | 26.89646 | 1.300237 | 3.48E-13 | 1.83E-12 |
| CD8A | 1.462335 | 6.811578 | 2.219715 | 1.53E-114 | 1.21E-112 |
| SLC8A2 | 0.509499 | 0.18722 | -1.44434 | 0.002116857 | 0.003476792 |
| AFF3 | 15.61571 | 7.399372 | -1.07752 | 7.31E-18 | 5.53E-17 |
| RCSD1 | 2.000929 | 4.767532 | 1.252573 | 4.88E-104 | 2.66E-102 |
| SLC12A3 | 0.070865 | 0.14309 | 1.013786 | 1.57E-73 | 4.44E-72 |
| PTAFR | 2.320499 | 6.089812 | 1.391963 | 1.97E-118 | 1.86E-116 |
| WARS | 17.06297 | 34.80637 | 1.028483 | 3.00E-50 | 5.54E-49 |
| RUNX3 | 2.838503 | 6.507506 | 1.196975 | 5.81E-93 | 2.46E-91 |
| GNLY | 0.632685 | 3.429734 | 2.438537 | 1.04E-102 | 5.43E-101 |
| GAB3 | 0.515601 | 1.300256 | 1.334469 | 1.50E-126 | 1.92E-124 |
| SIGLEC12 | 0.109697 | 0.423688 | 1.949471 | 3.21E-28 | 3.65E-27 |
| CD300LF | 0.76929 | 2.07447 | 1.431143 | 4.25E-124 | 5.02E-122 |
| P2RY14 | 0.466029 | 0.971714 | 1.060112 | 4.72E-66 | 1.18E-64 |
| GPBAR1 | 0.161754 | 0.347628 | 1.103743 | 7.05E-66 | 1.75E-64 |
| CCL13 | 0.577069 | 3.277969 | 2.505987 | 5.91E-84 | 2.11E-82 |
| PPBP | 0.06483 | 0.151781 | 1.227246 | 3.40E-05 | 7.19E-05 |
| FCAMR | 0.023402 | 0.262962 | 3.490156 | 1.42E-28 | 1.63E-27 |
| PSMB9 | 6.783432 | 19.2991 | 1.508446 | 2.93E-90 | 1.17E-88 |
| IPCEF1 | 0.203427 | 0.564341 | 1.472057 | 1.32E-93 | 5.67E-92 |
| ADGRG3 | 0.106051 | 0.264038 | 1.315989 | 3.28E-39 | 4.82E-38 |
| KLHDC7B | 2.555859 | 12.25598 | 2.261605 | 1.60E-41 | 2.46E-40 |
| CCDC69 | 2.949269 | 6.431026 | 1.124692 | 3.59E-71 | 9.70E-70 |
| PRDM8 | 0.214747 | 0.501215 | 1.222795 | 2.86E-82 | 9.84E-81 |
| PPP1R16B | 1.067745 | 2.855508 | 1.419181 | 1.07E-73 | 3.06E-72 |
| HLA-DPB1 | 43.49472 | 130.7332 | 1.587714 | 3.48E-137 | 1.14E-134 |
| CADM3 | 0.565729 | 1.376371 | 1.282687 | 4.40E-31 | 5.40E-30 |
| TLR10 | 0.229687 | 0.979373 | 2.092188 | 2.27E-84 | 8.15E-83 |
| MNDA | 3.152974 | 8.577974 | 1.443924 | 1.44E-121 | 1.58E-119 |
| THEMIS | 0.202311 | 0.878357 | 2.118234 | 5.01E-110 | 3.30E-108 |
| ADGRE5 | 4.432844 | 9.559963 | 1.108772 | 1.49E-86 | 5.51E-85 |
| FPR3 | 6.165323 | 17.2157 | 1.481476 | 4.23E-90 | 1.68E-88 |
| CCL4 | 1.576115 | 5.467923 | 1.79462 | 1.15E-116 | 1.00E-114 |
| MGAM2 | 0.311593 | 0.701917 | 1.17164 | 5.05E-06 | 1.19E-05 |
| PLCB2 | 1.251389 | 3.114336 | 1.315394 | 2.09E-121 | 2.25E-119 |
| LAG3 | 0.789351 | 3.427901 | 2.118587 | 1.48E-79 | 4.77E-78 |
| MAP4K1 | 1.055155 | 3.673806 | 1.79982 | 7.33E-125 | 9.04E-123 |
| CLEC9A | 0.103339 | 0.282712 | 1.451953 | 2.83E-56 | 5.86E-55 |
| CD1C | 0.720106 | 2.674699 | 1.893095 | 7.24E-63 | 1.69E-61 |
| DCDC1 | 0.48599 | 0.23018 | -1.07816 | 2.56E-09 | 9.11E-09 |
| NKG7 | 2.441021 | 14.79014 | 2.599079 | 1.72E-124 | 2.08E-122 |
| CD80 | 0.269619 | 0.659181 | 1.289753 | 1.96E-72 | 5.40E-71 |
| POU2F2 | 0.553586 | 1.608958 | 1.539248 | 1.12E-115 | 9.47E-114 |
| CLEC4E | 0.299547 | 1.395168 | 2.219585 | 9.01E-94 | 3.92E-92 |
| CXCR3 | 0.814181 | 4.797185 | 2.558767 | 4.30E-133 | 9.60E-131 |
| RLN2 | 3.063607 | 1.112711 | -1.46115 | 3.37E-07 | 9.25E-07 |
| PENK | 0.150673 | 0.779528 | 2.371182 | 8.55E-10 | 3.19E-09 |
| SLCO2B1 | 2.962723 | 6.827549 | 1.204444 | 5.08E-93 | 2.16E-91 |
| MYOG | 0.127683 | 0.044714 | -1.51376 | 2.95E-07 | 8.16E-07 |
| CCL7 | 0.41918 | 1.030081 | 1.297115 | 1.38E-22 | 1.31E-21 |
| TNFRSF9 | 0.214583 | 0.964981 | 2.168966 | 2.61E-116 | 2.25E-114 |
| ADAMDEC1 | 1.291666 | 7.202328 | 2.47923 | 1.04E-76 | 3.17E-75 |
| GDAP1L1 | 0.160931 | 0.053614 | -1.58575 | 3.15E-05 | 6.69E-05 |
| ARHGAP30 | 3.438232 | 9.498317 | 1.466005 | 1.25E-134 | 3.26E-132 |
| TRARG1 | 1.579113 | 3.552173 | 1.169588 | 4.17E-11 | 1.80E-10 |
| RARRES1 | 15.80231 | 41.40889 | 1.389805 | 1.47E-42 | 2.32E-41 |
| IGDCC3 | 0.907116 | 0.451571 | -1.00633 | 5.17E-06 | 1.22E-05 |
| OSCAR | 1.729119 | 3.670601 | 1.085979 | 1.34E-77 | 4.16E-76 |
| CD70 | 0.301126 | 0.795699 | 1.401853 | 1.51E-66 | 3.81E-65 |
| CD7 | 0.777152 | 3.632264 | 2.2246 | 3.35E-115 | 2.79E-113 |
| HAVCR2 | 3.181127 | 7.331961 | 1.204661 | 2.56E-117 | 2.32E-115 |
| ITGAL | 2.82125 | 7.317776 | 1.375071 | 2.77E-85 | 1.01E-83 |
| INSYN2 | 5.300959 | 1.74133 | -1.60606 | 3.25E-09 | 1.14E-08 |
| GLYATL2 | 10.17171 | 23.15818 | 1.18696 | 5.48E-06 | 1.29E-05 |
| PAX5 | 0.124144 | 0.68078 | 2.455169 | 3.03E-59 | 6.51E-58 |
| ICOS | 0.258635 | 1.610703 | 2.638698 | 9.39E-114 | 7.14E-112 |
| CDH7 | 0.21278 | 0.088312 | -1.26868 | 4.26E-07 | 1.15E-06 |
| TEX19 | 0.298014 | 0.138106 | -1.10961 | 0.029665499 | 0.040785708 |
| C14orf180 | 0.171296 | 0.356956 | 1.059252 | 7.75E-10 | 2.91E-09 |
| SOWAHD | 0.427259 | 0.886749 | 1.053415 | 5.71E-62 | 1.31E-60 |
| MCEMP1 | 0.126587 | 0.31239 | 1.30322 | 1.26E-20 | 1.11E-19 |
| TYROBP | 30.90328 | 77.07598 | 1.318521 | 3.32E-123 | 3.83E-121 |
| AGAP2 | 0.589405 | 1.264 | 1.100665 | 6.34E-89 | 2.46E-87 |
| SERPINB9 | 2.616729 | 5.512138 | 1.074848 | 5.16E-89 | 2.00E-87 |
| CCL2 | 7.667863 | 19.35516 | 1.335821 | 5.31E-76 | 1.59E-74 |
| NFAM1 | 1.311625 | 3.089897 | 1.236204 | 2.00E-98 | 9.50E-97 |
| KIR2DL4 | 0.04344 | 0.264128 | 2.604138 | 1.11E-65 | 2.74E-64 |
| CXorf65 | 0.136263 | 0.437551 | 1.683062 | 2.73E-65 | 6.65E-64 |
| SIRPB1 | 0.170099 | 0.520828 | 1.614429 | 9.87E-62 | 2.25E-60 |
| CD8B | 0.793951 | 3.038221 | 1.936105 | 1.53E-98 | 7.33E-97 |
| ARHGAP25 | 1.513319 | 4.340236 | 1.520057 | 2.00E-145 | 1.41E-142 |
| CSAG3 | 0.249341 | 0.507764 | 1.026038 | 3.11E-12 | 1.51E-11 |
| ETV7 | 1.77003 | 4.572034 | 1.369063 | 1.37E-55 | 2.82E-54 |
| NCF4 | 2.618433 | 7.3168 | 1.482509 | 1.43E-143 | 7.39E-141 |
| MS4A4A | 2.484777 | 6.713315 | 1.433909 | 2.51E-110 | 1.69E-108 |
| BCL2A1 | 2.194956 | 8.695208 | 1.986028 | 2.20E-97 | 1.03E-95 |
| PLA2G7 | 2.522332 | 6.69246 | 1.407778 | 1.25E-61 | 2.84E-60 |
| OR2I1P | 1.439279 | 8.627358 | 2.583573 | 1.27E-81 | 4.34E-80 |
| TEX11 | 0.066971 | 0.145329 | 1.11771 | 4.83E-45 | 8.12E-44 |
| VNN3 | 0.08951 | 0.179768 | 1.006013 | 1.15E-16 | 7.96E-16 |
| FGD2 | 0.385335 | 1.176239 | 1.609996 | 1.32E-130 | 2.32E-128 |
| MEFV | 0.086573 | 0.181571 | 1.068549 | 3.73E-50 | 6.86E-49 |
| IL7 | 0.300413 | 0.635311 | 1.080514 | 4.05E-51 | 7.63E-50 |
| UBD | 3.137197 | 16.81302 | 2.422031 | 1.58E-72 | 4.35E-71 |
| PTGER4 | 1.576066 | 3.591862 | 1.188404 | 9.33E-90 | 3.66E-88 |
| CCL3 | 2.208813 | 5.17085 | 1.22713 | 9.74E-63 | 2.26E-61 |
| CLEC12A | 0.228262 | 0.693374 | 1.602946 | 2.78E-90 | 1.12E-88 |
| TBC1D10C | 0.810124 | 2.970369 | 1.874428 | 1.05E-104 | 5.79E-103 |
| CCL5 | 9.761217 | 51.93418 | 2.411552 | 4.80E-128 | 6.54E-126 |
| TAP2 | 3.887582 | 8.298734 | 1.094018 | 5.27E-63 | 1.23E-61 |
| SASH3 | 3.155668 | 11.62989 | 1.88182 | 4.76E-160 | 3.34E-156 |
| PIK3CD | 1.702344 | 4.650759 | 1.449943 | 1.98E-119 | 1.93E-117 |
| SP140 | 0.358272 | 1.541105 | 2.104838 | 7.87E-133 | 1.68E-130 |
| CCL21 | 6.292159 | 22.91787 | 1.864846 | 4.12E-41 | 6.29E-40 |
| TRABD2A | 0.140232 | 0.368598 | 1.394235 | 3.51E-93 | 1.50E-91 |
| SCGN | 3.379771 | 1.20854 | -1.48366 | 0.000218034 | 0.000412273 |
| CPNE5 | 1.00919 | 2.134315 | 1.080576 | 2.00E-59 | 4.31E-58 |
| TNFRSF1B | 5.871688 | 17.014 | 1.534875 | 2.00E-147 | 1.76E-144 |
| KBTBD8 | 0.302219 | 0.681597 | 1.173322 | 8.15E-75 | 2.36E-73 |
| CCL17 | 0.735148 | 2.768669 | 1.913087 | 1.35E-65 | 3.31E-64 |
| VMO1 | 1.959359 | 8.09368 | 2.046414 | 8.97E-25 | 9.21E-24 |
| ARHGAP9 | 0.808873 | 3.084343 | 1.930979 | 1.24E-151 | 2.18E-148 |
| PNOC | 0.125912 | 0.666501 | 2.404187 | 2.61E-76 | 7.86E-75 |
| JSRP1 | 0.381169 | 1.155822 | 1.600417 | 1.91E-32 | 2.44E-31 |
| GRIA2 | 3.908277 | 1.860892 | -1.07054 | 5.07E-07 | 1.36E-06 |
| LAPTM5 | 38.77953 | 99.91802 | 1.365449 | 1.37E-144 | 8.36E-142 |
| CLLU1OS | 0.102629 | 0.404889 | 1.980087 | 1.87E-26 | 2.01E-25 |
| MMP7 | 28.84938 | 57.87247 | 1.004337 | 2.41E-25 | 2.52E-24 |
| IRF8 | 1.834207 | 6.314162 | 1.783435 | 2.61E-134 | 6.44E-132 |
| ACKR1 | 6.598443 | 17.06966 | 1.371237 | 1.72E-29 | 2.03E-28 |
| CTSG | 1.021877 | 2.064018 | 1.014234 | 1.01E-16 | 6.99E-16 |
| CD86 | 2.165191 | 5.955509 | 1.459731 | 2.33E-134 | 5.84E-132 |
| C1QC | 44.12429 | 124.0026 | 1.490725 | 4.64E-121 | 4.91E-119 |
| SLC9A9 | 1.071688 | 2.42608 | 1.178742 | 5.06E-103 | 2.66E-101 |
| TRPV2 | 2.349094 | 5.553991 | 1.241421 | 1.53E-130 | 2.62E-128 |
| TNFRSF13C | 0.391825 | 1.428363 | 1.866083 | 2.05E-39 | 3.03E-38 |
| CD8B2 | 0.219542 | 0.562216 | 1.356629 | 9.15E-20 | 7.72E-19 |
| CCL24 | 0.173759 | 0.45083 | 1.375499 | 1.30E-35 | 1.78E-34 |
| DPEP2 | 0.414795 | 1.096174 | 1.402008 | 2.04E-110 | 1.38E-108 |
| ARHGAP15 | 0.553591 | 1.578198 | 1.511387 | 4.53E-133 | 9.94E-131 |
| CHIT1 | 0.665437 | 4.757145 | 2.837722 | 1.90E-37 | 2.72E-36 |
| CD300LB | 0.236214 | 0.675118 | 1.515042 | 6.47E-68 | 1.67E-66 |
| PRKCQ | 0.390095 | 1.311152 | 1.748939 | 3.25E-102 | 1.68E-100 |
| RIMS4 | 9.078798 | 4.14728 | -1.13034 | 6.28E-10 | 2.38E-09 |
| DNASE1L3 | 0.23852 | 0.600726 | 1.332596 | 5.59E-45 | 9.39E-44 |
| CD84 | 1.316178 | 3.90975 | 1.570722 | 9.48E-105 | 5.22E-103 |
| HTR3A | 0.109558 | 0.3721 | 1.763998 | 1.66E-18 | 1.31E-17 |
| CD96 | 0.549498 | 2.671885 | 2.28167 | 1.17E-128 | 1.74E-126 |
| ADAMTS19 | 0.635936 | 0.307577 | -1.04793 | 3.20E-07 | 8.81E-07 |
| ERVFRD-1 | 0.083797 | 0.217185 | 1.373955 | 2.49E-07 | 6.94E-07 |
| POU2AF1 | 0.403636 | 2.313566 | 2.51899 | 5.46E-72 | 1.49E-70 |
| ITGAX | 1.887563 | 4.871507 | 1.367844 | 3.78E-117 | 3.39E-115 |
| LRTM2 | 0.699687 | 0.23625 | -1.5664 | 0.015343627 | 0.022130292 |
| TNFSF18 | 0.165148 | 0.36696 | 1.151861 | 4.27E-34 | 5.65E-33 |
| LILRB4 | 1.83294 | 5.785609 | 1.658309 | 4.21E-129 | 6.43E-127 |
| CD72 | 0.759248 | 2.185111 | 1.525063 | 1.49E-109 | 9.66E-108 |
| TLR4 | 2.398247 | 4.825439 | 1.00868 | 1.37E-70 | 3.62E-69 |
| MCOLN2 | 0.814247 | 1.762923 | 1.114431 | 3.03E-64 | 7.27E-63 |
| CD1E | 0.453665 | 1.756405 | 1.952927 | 1.48E-65 | 3.64E-64 |
| LEP | 0.956705 | 2.28421 | 1.255549 | 1.29E-11 | 5.86E-11 |
| S100A8 | 42.29462 | 113.7976 | 1.427924 | 3.88E-35 | 5.27E-34 |
| RTL9 | 0.36618 | 0.075759 | -2.27306 | 0.004474749 | 0.007005939 |
| FERMT3 | 4.0359 | 12.58142 | 1.640333 | 5.64E-149 | 6.10E-146 |
| BMP3 | 0.070626 | 0.145105 | 1.038819 | 8.79E-05 | 0.000175536 |
| IL21R | 0.395609 | 1.656589 | 2.066067 | 1.49E-119 | 1.46E-117 |
| PTCRA | 0.08713 | 0.315906 | 1.858258 | 1.55E-86 | 5.70E-85 |
| ACSL5 | 3.966519 | 8.06225 | 1.023309 | 1.42E-60 | 3.13E-59 |
| CALHM6 | 2.622031 | 9.444423 | 1.848778 | 2.04E-114 | 1.59E-112 |
| CTSS | 12.73075 | 37.42152 | 1.55555 | 1.37E-120 | 1.39E-118 |
| IL7R | 1.63531 | 8.124081 | 2.31264 | 2.11E-106 | 1.22E-104 |
| PDCD1 | 0.313505 | 1.876328 | 2.581354 | 5.98E-118 | 5.49E-116 |
| TIFAB | 0.043892 | 0.299686 | 2.771434 | 8.64E-114 | 6.60E-112 |
| RP1 | 0.184881 | 0.081943 | -1.17391 | 0.000249162 | 0.000467919 |
| CXCL1 | 1.065045 | 2.920838 | 1.455468 | 1.27E-25 | 1.34E-24 |
| APOBEC3H | 0.300614 | 1.059705 | 1.817678 | 6.84E-112 | 4.85E-110 |
| SLC2A5 | 0.523957 | 1.280054 | 1.288685 | 2.69E-66 | 6.74E-65 |
| KLRK1 | 0.056045 | 0.188108 | 1.746899 | 7.02E-66 | 1.74E-64 |
| KLHL41 | 0.565707 | 0.261926 | -1.1109 | 0.000379152 | 0.000696218 |
| CD274 | 0.733199 | 1.930885 | 1.396985 | 4.03E-77 | 1.24E-75 |
| PEX5L | 0.813293 | 0.33773 | -1.2679 | 1.61E-10 | 6.55E-10 |
| GIMAP5 | 0.127276 | 0.397581 | 1.643293 | 4.02E-90 | 1.60E-88 |
| FAM135B | 0.398415 | 0.114527 | -1.79858 | 2.43E-08 | 7.68E-08 |
| APBB1IP | 2.402571 | 6.301114 | 1.391028 | 1.23E-131 | 2.38E-129 |
| TM4SF19 | 0.194672 | 0.477423 | 1.29422 | 2.50E-11 | 1.11E-10 |
| FAM57B | 0.699877 | 0.335332 | -1.06151 | 1.47E-11 | 6.64E-11 |
| SMCO3 | 1.45584 | 0.57 | -1.35282 | 0.018538897 | 0.026360053 |
| STAT4 | 0.469962 | 1.549386 | 1.72108 | 2.19E-112 | 1.59E-110 |
| TMPRSS11E | 1.377481 | 0.679056 | -1.02043 | 0.018010346 | 0.025663054 |
| HLA-DOA | 4.168833 | 12.92481 | 1.632427 | 2.33E-120 | 2.34E-118 |
| CCR7 | 1.511751 | 5.644951 | 1.90074 | 5.81E-106 | 3.33E-104 |
| CSF3 | 0.051331 | 0.151564 | 1.56204 | 4.35E-11 | 1.88E-10 |
| TMEM150B | 0.301465 | 1.010604 | 1.745157 | 1.34E-99 | 6.54E-98 |
| CSF2RA | 1.492821 | 3.533524 | 1.243067 | 2.13E-103 | 1.14E-101 |
| IGLL5 | 6.665351 | 40.02283 | 2.58607 | 4.82E-58 | 1.02E-56 |
| CLEC4C | 0.032181 | 0.240387 | 2.901094 | 1.60E-77 | 4.95E-76 |
| CD27 | 1.371501 | 7.315898 | 2.41528 | 2.61E-118 | 2.44E-116 |
| GBP5 | 0.925107 | 6.896298 | 2.89813 | 3.78E-118 | 3.51E-116 |
| SOCS1 | 1.702215 | 4.534408 | 1.413501 | 3.13E-79 | 9.99E-78 |
| CD52 | 8.484347 | 55.45632 | 2.708476 | 2.08E-127 | 2.73E-125 |
| CYP17A1 | 0.376108 | 0.099308 | -1.92117 | 0.000941893 | 0.001634903 |
| UTS2 | 0.075115 | 0.256184 | 1.769999 | 7.84E-53 | 1.51E-51 |
| BMPR1B | 45.17059 | 21.4146 | -1.07679 | 1.96E-06 | 4.87E-06 |
| SPN | 0.805729 | 3.030623 | 1.911248 | 1.21E-138 | 4.60E-136 |
| APOL1 | 16.30328 | 37.09368 | 1.186011 | 1.64E-90 | 6.64E-89 |
| PLA2G2D | 0.315695 | 4.323333 | 3.775543 | 9.35E-96 | 4.23E-94 |
| CD3D | 2.126557 | 13.2261 | 2.636796 | 4.36E-139 | 1.70E-136 |
| DOK2 | 1.344312 | 5.196202 | 1.950589 | 3.28E-136 | 1.00E-133 |
| FMO3 | 1.276255 | 3.048391 | 1.256132 | 9.92E-25 | 1.02E-23 |
| SLC7A7 | 2.361317 | 5.709081 | 1.273667 | 2.48E-121 | 2.64E-119 |
| CD38 | 0.269991 | 1.734022 | 2.68314 | 5.34E-100 | 2.65E-98 |
| TNFAIP3 | 4.929983 | 11.66342 | 1.242336 | 4.00E-93 | 1.70E-91 |
| BHLHE22 | 0.355483 | 0.941079 | 1.404534 | 3.70E-61 | 8.32E-60 |
| FLI1 | 1.606375 | 3.37395 | 1.07063 | 7.86E-100 | 3.89E-98 |
| INPP5D | 1.855891 | 4.83908 | 1.382621 | 5.20E-123 | 5.94E-121 |
| CCR2 | 0.582658 | 2.606931 | 2.161632 | 3.48E-112 | 2.51E-110 |
| GZMK | 1.075285 | 7.16543 | 2.736334 | 5.99E-121 | 6.28E-119 |
| IFI16 | 11.20446 | 22.88333 | 1.030223 | 1.10E-85 | 4.00E-84 |
| P2RY6 | 0.994378 | 2.02795 | 1.028156 | 2.51E-71 | 6.79E-70 |
| TREML2 | 0.041299 | 0.234935 | 2.508087 | 7.63E-89 | 2.94E-87 |
| TNIP3 | 0.081347 | 0.380073 | 2.224118 | 4.13E-97 | 1.89E-95 |
| SCUBE1 | 1.38598 | 0.672286 | -1.04376 | 0.022087584 | 0.031029059 |
| LAMP3 | 1.864019 | 6.389144 | 1.777206 | 5.38E-81 | 1.80E-79 |
| SIGLEC1 | 1.685051 | 4.243932 | 1.332609 | 3.50E-73 | 9.87E-72 |
| FCER2 | 0.063016 | 0.96147 | 3.931454 | 1.09E-72 | 3.04E-71 |
| NPC1L1 | 0.527618 | 0.241301 | -1.12866 | 8.89E-13 | 4.53E-12 |
| HTRA4 | 0.191085 | 0.546074 | 1.514882 | 3.30E-73 | 9.30E-72 |
| CLEC4D | 0.032643 | 0.267382 | 3.034061 | 3.52E-60 | 7.70E-59 |
| LY6D | 4.341225 | 9.407891 | 1.115769 | 5.36E-11 | 2.29E-10 |
| HLA-E | 123.6491 | 264.7741 | 1.09851 | 3.07E-109 | 1.94E-107 |
| KCNA3 | 0.196514 | 0.853038 | 2.117979 | 2.94E-89 | 1.15E-87 |
| TF | 1.834987 | 3.825659 | 1.059938 | 1.81E-08 | 5.85E-08 |
| SH2D1A | 0.419326 | 2.635546 | 2.651958 | 2.97E-127 | 3.87E-125 |
| C4BPA | 0.723373 | 1.606688 | 1.151279 | 6.24E-07 | 1.65E-06 |
| NUGGC | 0.108098 | 0.503247 | 2.218926 | 2.09E-87 | 7.85E-86 |
| CST7 | 2.673703 | 11.46467 | 2.100283 | 2.17E-132 | 4.41E-130 |
| ADGRE1 | 0.233097 | 0.526254 | 1.17483 | 4.67E-76 | 1.40E-74 |
| APOC1 | 27.84294 | 68.2367 | 1.293236 | 1.81E-65 | 4.41E-64 |
| C16orf54 | 0.666026 | 2.691408 | 2.014711 | 1.25E-130 | 2.22E-128 |
| MS4A6E | 0.026115 | 0.15098 | 2.53139 | 2.84E-12 | 1.38E-11 |
