# Supplementary material for: CCL19 has potential to be a potential prognostic biomarker and a modulator of tumor immune microenvironment (TIME) of breast cancer: a comprehensive analysis based on TCGA database
Source: Aging (Albany NY). 2022 May 12;14(9):4158–75. doi: 10.18632/aging.204081 (PMC9134962; doi:10.18632/aging.204081)
Supplement: Supplementary Table 4 [file aging-14-204081-s005.docx]

**Supplementary Table 4. DEGs shared by stromal scores and immune scores.**

| **Gene** | **logFC** |
| --- | --- |
| JAML | 1.585118 |
| SIT1 | 1.744263 |
| MMP8 | 1.242079 |
| NCKAP1L | 1.370889 |
| CMA1 | 1.330421 |
| EVI2B | 1.429197 |
| CAMK4 | 1.02805 |
| IL10RA | 1.394869 |
| EGFL6 | 1.262992 |
| JCHAIN | 1.342573 |
| ITK | 1.906701 |
| CD48 | 1.583928 |
| CD2 | 1.74073 |
| TESPA1 | 1.601648 |
| KLHL6 | 1.417691 |
| SCARA5 | 1.471126 |
| CD226 | 1.36324 |
| WDFY4 | 1.412267 |
| FCGR3B | 1.402254 |
| MAP1LC3C | 1.36539 |
| ZNF831 | 1.913639 |
| SLC22A3 | 1.04161 |
| ABI3BP | 1.251424 |
| TNFSF8 | 1.284807 |
| TLR8 | 1.620497 |
| FCN1 | 1.692817 |
| CCL23 | 1.371099 |
| PRKCB | 1.807748 |
| IL16 | 1.301162 |
| CTSE | 1.580449 |
| WIPF1 | 1.14911 |
| FABP4 | 1.579758 |
| ROS1 | 2.278373 |
| TPSD1 | 1.282994 |
| CLEC10A | 1.766004 |
| PI16 | 1.525335 |
| CPA1 | 1.401712 |
| BTLA | 1.983769 |
| MPEG1 | 1.365169 |
| CD69 | 1.552717 |
| PRDM1 | 1.039275 |
| DMBT1 | 3.339813 |
| PLEK | 1.432478 |
| EPYC | 1.460471 |
| LYZ | 1.615312 |
| FCRL3 | 2.044598 |
| ICAM3 | 1.624238 |
| TFEC | 1.329854 |
| AICDA | 3.680411 |
| SCML4 | 1.850177 |
| FYB1 | 1.413641 |
| TLR7 | 1.198157 |
| VCAM1 | 1.330475 |
| MS4A1 | 2.348796 |
| KLRB1 | 1.54842 |
| LAX1 | 1.805875 |
| FCRL5 | 1.927153 |
| FCRL1 | 2.546801 |
| LY9 | 1.856469 |
| SLFN12L | 1.622273 |
| NLRP3 | 1.200309 |
| CHRDL1 | 1.368589 |
| TNFRSF17 | 1.675682 |
| IL12B | 1.724264 |
| GPR15 | 1.652364 |
| CD5 | 1.73798 |
| EOMES | 1.848609 |
| SELP | 1.364235 |
| AGXT | 1.435679 |
| GIMAP6 | 1.050016 |
| SELE | 1.073455 |
| CNR2 | 2.084786 |
| SLAMF1 | 1.827028 |
| CD40LG | 1.753516 |
| LILRB5 | 1.327722 |
| AOAH | 1.406207 |
| FCRL6 | 1.410273 |
| PTGDS | 1.769119 |
| CD209 | 1.381283 |
| CD200R1 | 1.350372 |
| CSF2RB | 1.517237 |
| CCL19 | 1.88466 |
| SULT1B1 | 1.692685 |
| PARP15 | 1.670347 |
| GRAP2 | 1.354926 |
| IL6 | 1.312968 |
| SLAMF6 | 1.91478 |
| UBASH3A | 1.693885 |
| BLK | 2.063634 |
| GZMM | 1.745263 |
| CASS4 | 1.130833 |
| GLYAT | 1.477154 |
| PYHIN1 | 1.821125 |
| CLEC4G | 1.293507 |
| DPT | 1.471185 |
| GIMAP7 | 1.234815 |
| EVI2A | 1.239977 |
| CD300LG | 1.383905 |
| CRTAM | 1.723225 |
| DOCK2 | 1.373704 |
| CYBB | 1.350435 |
| GIMAP8 | 1.012267 |
| C1S | 1.102559 |
| SAMD3 | 1.652806 |
| CD3E | 1.802782 |
| CCR5 | 1.563764 |
| CH25H | 1.221498 |
| CR2 | 2.110766 |
| TCL1A | 2.509365 |
| XCR1 | 1.708381 |
| P2RY13 | 1.171448 |
| LILRA4 | 1.458637 |
| GPR174 | 1.977152 |
| PIK3CG | 1.416211 |
| FCRLA | 1.969542 |
| G0S2 | 1.187229 |
| IKZF1 | 1.517381 |
| IL9R | 1.372137 |
| MMP9 | 1.666925 |
| FGL2 | 1.395426 |
| DCSTAMP | 1.383058 |
| CCR4 | 1.7369 |
| MEOX1 | 1.104342 |
| LGI2 | 1.06149 |
| RGS18 | 1.26802 |
| XPNPEP2 | 1.44077 |
| CCL11 | 1.266532 |
| CR1 | 1.671046 |
| CLEC17A | 1.655844 |
| GIMAP1 | 1.149845 |
| TMEM273 | 1.152649 |
| GAPT | 1.06292 |
| GPR171 | 1.714614 |
| FPR1 | 1.171744 |
| CD28 | 1.474943 |
| PTPRC | 1.686757 |
| TRAT1 | 2.001428 |
| FOLR2 | 1.343342 |
| RGS13 | 1.118862 |
| ABCD2 | 1.361717 |
| GPR183 | 1.36447 |
| CD3G | 1.834556 |
| GAB3 | 1.187555 |
| FCAMR | 2.556299 |
| CCDC69 | 1.074152 |
| PRDM8 | 1.179282 |
| PPP1R16B | 1.21466 |
| TLR10 | 1.575074 |
| THEMIS | 1.651282 |
| CLEC9A | 1.265268 |
| CD1C | 1.533513 |
| PENK | 2.115129 |
| SLCO2B1 | 1.128596 |
| TRARG1 | 1.405487 |
| C14orf180 | 1.363713 |
| TEX11 | 1.103741 |
| PTGER4 | 1.097556 |
| VMO1 | 1.831505 |
| PNOC | 1.762363 |
| ACKR1 | 1.464473 |
| CTSG | 1.246059 |
| SLC9A9 | 1.095285 |
| CHIT1 | 2.32318 |
| CD300LB | 1.370873 |
| DNASE1L3 | 1.189165 |
| CD84 | 1.408243 |
| CD96 | 1.667415 |
| ERVFRD-1 | 1.692147 |
| TLR4 | 1.095219 |
| CD1E | 1.52194 |
| LEP | 1.826675 |
| IL21R | 1.553931 |
| IL7R | 1.84745 |
| GIMAP5 | 1.408804 |
| APBB1IP | 1.195704 |
| CLEC4C | 2.163969 |
| CD27 | 1.709607 |
| CD52 | 2.126644 |
| SPN | 1.500331 |
| PLA2G2D | 2.445248 |
| BHLHE22 | 1.398082 |
| FLI1 | 1.056643 |
| CCR2 | 1.667069 |
| GZMK | 2.017296 |
| TREML2 | 1.787356 |
| FCER2 | 2.498611 |
| CLEC4D | 2.417317 |
| KCNA3 | 1.743555 |
| SH2D1A | 1.894595 |
| NUGGC | 1.685419 |
| CST7 | 1.558331 |
| C16orf54 | 1.564657 |
| MS4A6E | 2.52935 |
| RUNDC3A | -2.11216 |
| MYT1 | -1.89968 |
| TMEM145 | -1.41735 |
| FCRLB | -1.56753 |
| KCNH6 | -2.04112 |
| PRTN3 | -1.20918 |
| C8orf86 | -1.55657 |
| CST9L | -1.13439 |
| RPL3L | -2.05756 |
| TMEM82 | -1.29803 |
| NEURL1 | -1.04134 |
| EEF1A2 | -1.09068 |
| SLC1A2 | -1.14252 |
| SLC27A2 | -1.20067 |
| PAH | -1.2904 |
| APLP1 | -1.37348 |
| TMPRSS6 | -1.05065 |
| BRINP2 | -1.61366 |
| ASCL1 | -1.66117 |
| CELF3 | -1.87817 |
| RTBDN | -1.60934 |
| CPLX2 | -3.30074 |
| SYT13 | -1.26283 |
| COL2A1 | -1.20433 |
| SLC8A2 | -1.7511 |
| MYOG | -1.46566 |
| TEX19 | -1.49349 |
| LRTM2 | -1.73375 |
| PEX5L | -1.18933 |
| FAM57B | -1.13251 |
