# Supplementary material for: CCL19 has potential to be a potential prognostic biomarker and a modulator of tumor immune microenvironment (TIME) of breast cancer: a comprehensive analysis based on TCGA database
Source: Aging (Albany NY). 2022 May 12;14(9):4158–75. doi: 10.18632/aging.204081 (PMC9134962; doi:10.18632/aging.204081)
Supplement: Supplementary Tables 5 and 6 [file aging-14-204081-s006.pdf]

Supplementary Table 5. The most significant DEGs regarding the survival of breast cancer patients through univariate COX regression analysis.

Gene	KM	HR	HR.95L	HR.95H	pvalue
JAML	0.00844	0.819776	0.699638	9.61E-01	1.40E-02
SIT1	0.017771	0.924622	0.861224	9.93E-01	3.06E-02
CAMK4	0.003356	0.543579	0.303225	9.74E-01	4.07E-02
ITK	0.013786	0.834153	0.701476	9.92E-01	4.02E-02
CD48	0.002759	0.95801	0.925341	9.92E-01	1.54E-02
CD2	0.00258	0.982164	0.967314	9.97E-01	2.06E-02
TESPA1	0.00079	0.761623	0.602446	9.63E-01	2.28E-02
CD226	0.004438	0.453861	0.221812	9.29E-01	3.06E-02
CLEC10A	0.013528	0.922159	0.856394	9.93E-01	3.18E-02
SCML4	0.007672	0.543369	0.298932	9.88E-01	4.54E-02
KLRB1	0.000172	0.815782	0.72541	9.17E-01	6.77E-04
IL12B	0.001517	0.230377	0.073881	7.18E-01	1.14E-02
CD5	0.002018	0.937312	0.885128	9.93E-01	2.68E-02
CD40LG	0.0025	0.823188	0.693335	9.77E-01	2.63E-02
CCL19	0.00907	0.994301	0.989772	9.99E-01	1.41E-02
SLAMF6	0.047425	0.936356	0.880764	9.95E-01	3.52E-02
UBASH3A	0.007546	0.765376	0.612384	9.57E-01	1.88E-02
GZMM	0.020157	0.906528	0.835051	9.84E-01	1.92E-02
CRTAM	0.023505	0.742985	0.565605	9.76E-01	3.28E-02
C1S	0.015332	0.99602	0.992255	1.00E+00	3.91E-02
CD3E	0.000502	0.970539	0.948205	9.93E-01	1.18E-02
XCR1	0.02717	0.593692	0.355016	9.93E-01	4.69E-02
GPR171	0.002284	0.816966	0.698459	9.56E-01	1.15E-02
TRAT1	0.001064	0.828631	0.688185	9.98E-01	4.73E-02
CLEC9A	0.030918	0.358115	0.154166	8.32E-01	1.69E-02
CD1E	0.033671	0.872044	0.770953	9.86E-01	2.94E-02
IL7R	0.014608	0.9684	0.939735	9.98E-01	3.62E-02
CD27	0.016712	0.953271	0.916732	9.91E-01	1.64E-02
CD52	5.64E-05	0.988215	0.980241	9.96E-01	4.13E-03
SPN	0.022025	0.892886	0.807906	9.87E-01	2.64E-02
SH2D1A	0.002603	0.900706	0.819404	9.90E-01	3.03E-02
CST7	0.0074	0.958468	0.931483	9.86E-01	3.60E-03

Supplementary Table 6. The difference test and correlation test between the expression of CCL19 and TICs.

TICs	Correlation test (p-value)	Difference test (p-value)
Macrophages M2	<0.001	<0.001
T cells CD8	<0.001	<0.001
Macrophages M1	<0.001	<0.001
Macrophages M0	<0.001	<0.001
T cells gamma delta	<0.001	<0.001
T cells CD4 memory activated	<0.001	<0.001
B cells naive	<0.001	<0.001
T cells CD4 memory resting	<0.001	<0.001
Dendritic cells resting	<0.001	<0.001
NK cells resting	<0.001	<0.001
B cells memory	<0.001	<0.001
Mast cells activated	<0.001	<0.001
T cells regulatory (Tregs)	<0.001	>0.05
T cells follicular helper	0.001	<0.001
Dendritic cells activated	0.012	<0.001
Neutrophils	<0.001	<0.001
